# A large-scale *in vivo* RNAi screen to identify genes involved in Notch-mediated follicle cell differentiation and cell cycle switches

**DOI:** 10.1038/srep12328

**Published:** 2015-07-24

**Authors:** Dongyu Jia, Muhammed Soylemez, Gabriel Calvin, Randy Bornmann, Jamal Bryant, Cameron Hanna, Yi-Chun Huang, Wu-Min Deng

**Affiliations:** 1 Department of Biological Science, Florida State University, Tallahassee, FL 32306-4370, USA

## Abstract

During *Drosophila* oogenesis, follicle cells sequentially undergo three distinct cell-cycle programs: the mitotic cycle, endocycle, and gene amplification. Notch signaling plays a central role in regulating follicle-cell differentiation and cell-cycle switches; its activation is essential for the mitotic cycle/endocycle (M/E) switch. Cut, a linker between Notch signaling and cell-cycle regulators, is specifically downregulated by Notch during the endocycle stage. To determine how signaling pathways coordinate during the M/E switch and to identify novel genes involved in follicle cell differentiation, we performed an *in vivo* RNAi screen through induced knockdown of gene expression and examination of Cut expression in follicle cells. We screened 2205 RNAi lines and found 33 genes regulating Cut expression during the M/E switch. These genes were confirmed with the staining of two other Notch signaling downstream factors, Hindsight and Broad, and validated with multiple independent RNAi lines. We applied gene ontology software to find enriched biological meaning and compared our results with other publications to find conserved genes across tissues. Specifically, we found earlier endocycle entry in anterior follicle cells than those in the posterior, identified that the insulin-PI3K pathway participates in the precise M/E switch, and suggested Nejire as a cofactor of Notch signaling during oogenesis.

The *Drosophila* egg chamber, the developmental unit of oogenesis, consists of sixteen germ-line cells: one oocyte and fifteen nurse cells, which are covered by a single layer of somatic follicle cells. The follicular epithelium is an excellent model system for the study of cell cycle regulation and cell differentiation in development. During oogenesis, follicle cells sequentially transition between three distinct cell cycle programs: the mitotic cycle (early oogenesis, stages 1–6), endocycle (midoogenesis, stages 7–10A) and gene amplification (late oogenesis, stages 10B–13). Previous studies have identified multiple signaling pathways, such as Notch, Hedgehog, EGFR, Wingless, JAK/STAT, Hippo and JNK, as being involved in spatiotemporal regulation of follicle cell differentiation during different stages of oogenesis^1^. Among these pathways, Notch signaling is crucial. Its activation and inactivation in follicle cells is essential for the mitotic cycle/endocycle (M/E) and the endocycle/gene amplification switches, respectively^1^.

Just before the onset of the endocycle, at stage 5 of *Drosophila* oogenesis, ligand Delta is highly upregulated in the germline. Germline Delta then binds to the Notch receptor in the somatic follicle cells to initiate signaling. Notch is a single transmembrane protein, which undergoes cleavage after its extracellular domain interacts with ligand Delta. The Notch intercellular domain (NICD) migrates into the nucleus upon being cleaved by the gamma-secretase complex^2^,^3^. Inside the nucleus, NICD, together with co-activator Mastermind (Mam), interacts with the CBF1/Suppressor of Hairless/LAG-1 (CSL) transcription repressor (Suppressor of Hairless [Su(H)] in *Drosophila*), changing Su(H) from a repressor to an activator by forming the NICD/Mam/Su(H) trimeric complex, and thus inducing transcription of downstream genes^4^. After Notch signaling activation, the follicle cells undergo the M/E switch and cell differentiation^2^,^3^.

Cut is a homeodomain-containing transcription factor that was previously identified as a downstream target negatively regulated by Notch signaling during the M/E switch, In this process, Cut serves as a linker between Notch signaling and cell cycle regulators. The expression of Cut is observed in the mitotic cycle (Fig. 1A), which is then specifically downregulated by Notch signaling during the endocycle in the mainbody follicle cells^5^. This downregulation of Cut by Notch is critical for proper follicle cell differentiation from immature cell fate to mature cell fate, and its continued expression beyond stage 6 in mainbody follicle cells can be used as a marker for failed M/E transition^5^.

RNA interference (RNAi) utilizes short double-stranded RNA (dsRNA) molecules and acts through the RNA-induced silencing complex (RISC) for gene silencing. The RNAi machinery recognizes its RNA targets through dsRNA in a sequence-specific manner, and can efficiently knock down endogenous or exogenously introduced dsRNAs^6^. The RNAi technique has been well developed and widely used to study gene function, and has become a popular and reliable tool to greatly enhance understanding of molecular and genetic mechanisms of human diseases, leading to promising therapeutic application^7^,^8^,^9^. By using genome-wide RNAi libraries, almost any gene can be selectively silenced, and the process can be accomplished in a high throughput and unbiased manner. In the past several years, a number of genome-scale RNAi high-throughput screens have been done in cultured cells and tissues of both *Drosophila* and mammals to study different biological processes including signal transduction^10^, cancer biology^11^, epithelial development^12^, stem cell identity^13^, and host cell responses to infection^14^,^15^. From these screens, scientists have identified many new components of these biological processes and have gained more insight into the complexity of biological systems.

In this study, we applied RNAi-induced gene-specific silencing to perform a large-scale *in vivo* RNAi screen to identify genes involved in follicle cell differentiation and cell cycle switches by analyzing Cut expression during *Drosophila* oogenesis. Our screen identified 33 genes out of 2205 RNAi lines, 9 of which had heretofore not been linked to Notch signaling. In addition, we found the anterior follicle cells enter endocycle earlier than their posterior counterparts, identified that the Insulin-PI3K pathway participates in the precise M/E switch, and suggested the involvement of a cofactor Nej in Notch signaling during *Drosophila* oogenesis.

## Results

### A large-scale *in vivo* RNAi screen to identify genes involved in follicle cell differentiation and cell cycle switching by analyzing Cut

**Figure 1:**
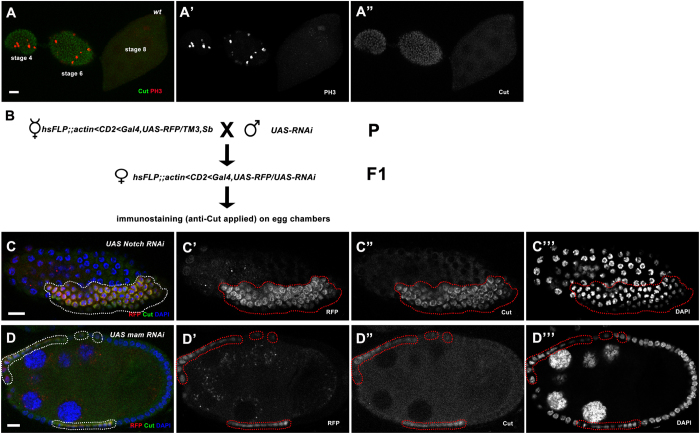
The strategy of our *in vivo* RNAi screen to identify genes involved in follicle cell differentiation and cell cycle switches. (**A**-**A”**) In wild-type follicle cells, Cut expression is present during early oogenesis, but disappears in mainbody follicle cells during mid-oogenesis. PH3 staining in follicle cells was used to indicate early-stage (stages 1–6) egg chambers. (**B**) Crossing scheme for the *in vivo* RNAi screen. (**C**-**C”’**) Follicle cells expressing *Notch* RNAi in a stage-7 egg chamber showed upregulated Cut expression and small nuclei. (**D**-**D”’**) Follicle cells expressing *mam* RNAi in a stage-8 egg chamber showed upregulated Cut expression and small nuclei. DAPI staining marks cell nuclei. Anterior is to the left. Clone region marked by presence of RFP and outlined with dotted lines. Bars, 10 μm.

To understand how the Notch signaling pathway actively participates during the M/E switch and to identify novel genes involved in follicle cell differentiation, we planned a genome-wide *in vivo* RNAi screen through induced knockdown of gene expression by flip-out GAL4-UAS RNAi system and examination of Cut expression in follicle cells (Fig. 1B). RNAi lines were obtained from collection of the Transgenic RNAi Project (TRiP) in the Bloomington *Drosophila* Stock Center.

Before executing a large scale *in vivo* RNAi screen, we tested the efficacy of the flip-out GAL4-UAS RNAi screening strategy. Several RNAi lines targeting Notch pathway components were applied, and defects in the M/E switch as well as prolonged Cut expression in follicle cells were observed after induced RNAi expression. For example, during midoogenesis (stages 7–10A), knockdown of Notch induced Cut upregulation and the small nuclei phenotype (Fig. 1C). As a co-activator of Notch and key component of NICD/Mam/Su(H) trimeric complex^4^, Mam knockdown showed a similar phenotype as Notch knockdown (Fig. 1D). These results indicate the flip-out GAL4-UAS RNAi system could efficiently reduce Notch components and inhibit proper endocycle entry in follicle cells, thus demonstrating the large-scale *in vivo* screen strategy was technically feasible and that it could help us identify additional genes involved in follicle cell differentiation and cell cycle switches. We then performed a double-blind screen, during which 2205 RNAi lines (Supplemental Table 1) were randomly chosen and only Bloomington Stock Numbers (BL#) were labeled during the screen to avoid any bias, and found 33 lines showing Cut expression defects during M/E switch (Table 1).

### Genes validated via Hindsight (Hnt) and Broad (Br) expression

**Figure 2:**
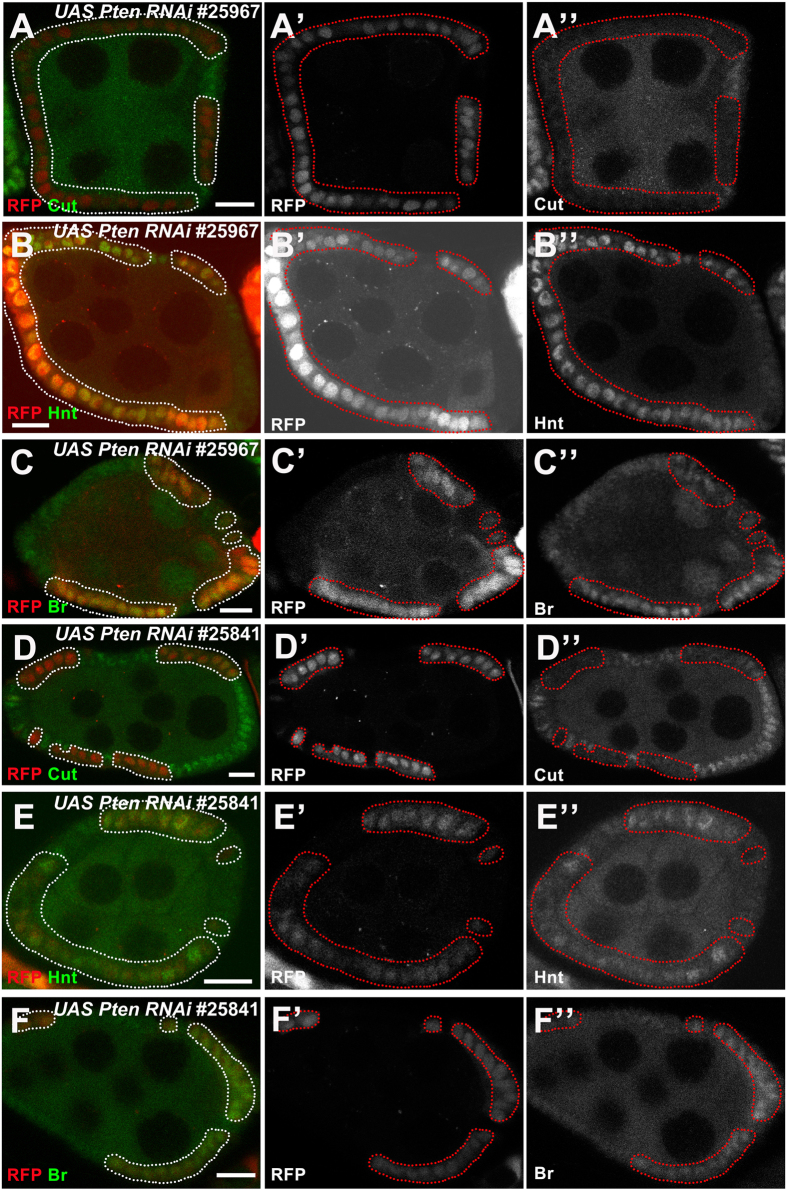
Validation of identified genes from the screen. (**A**-**A”**) Candidate genes were identified by Cut expression. Downregulation of Cut (green in **A**, white in **A”**) was observed in follicle cells expressing *Pten* RNAi (BL#25967). (**B**-**C**) Hnt and Cut stainings were applied to validate identified genes. (**B**-**B”**) Upregulation of Hnt (green in **B**, white in **B”**) was observed in *Pten* RNAi (BL#25967) follicle cells. (**C**-**C”**) Upregulation of Br (green in **C**, white in **C”**) was observed in *Pten* RNAi (BL#25967) follicle cells. (**D**-**F”**) Different RNAi lines were examined to validate identified genes. Downregulation of Cut (green in **D**, white in **D”**), upregulation of Hnt (green in **E**, white in **E”**) and Br (green in **F**, white in **F”**) were observed in follicle cells expressing *Pten* RNAi (BL#25841). Anterior is to the left. Clone region marked by presence of RFP and outlined with dotted lines. Bars, 10 μm.

In addition to Cut, Hnt (also known as Pebbled), a C2H2 Zinc finger transcription factor, is involved in Notch signaling during *Drosophila* oogenesis. Its expression is specifically induced by Notch during the endocycle stage to mediate Notch-dependent downregulation of Cut, String and Hedgehog signaling in follicle cells during midoogenesis^16^. Both Cut and Hnt have been implied to be regulated directly by Notch via Su(H)^17^. Br, another C2H2 Zinc finger transcription factor, is upregulated in the mainbody follicle cells during the M/E switch directly by Notch signaling via Su(H) binding to its *brE* enhancer region^18^. Both Hnt and Br expression were observed in order to validate all genes in Table 1. For example, knockdown of Pten led to downregulation of Cut in early oogenesis (Fig. 2A), concomitant with upregulation of Hnt and Br (Fig. 2B,C). This effect of Pten knockdown on the M/E switch was not very strong; a phenotype was only detectable around stages 5/6.

### Multiple lines were examined to validate genes

To reduce the chance of off-target effects, we either tested multiple RNAi lines for the same gene, from Bloomington Stock Center or NIG-FLY, or mutant lines in order to confirm the phenotype (Table 1). For example, knockdown of Pten by one RNAi line (BL#25967) induced early entry into the endocycle, as indicated by precocious downregulation of Cut and upregulation of Hnt and Br in early oogenesis (Fig. 2A–C). We further tested another *Pten* RNAi line (BL#25841), which showed consistent phenotypes (Fig. 2D–F).

### Analysis of identified genes from the screen

**Figure 3:**
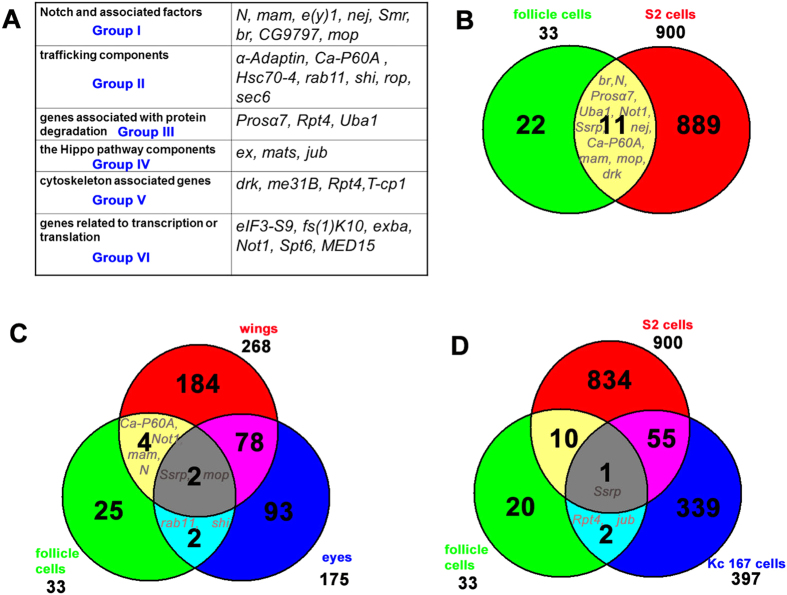
Six categories of identified genes, and Venn diagrams depicting the overlap between screen data from follicle cells and other experimental data from S2 cells, *Drosophila* wings and eyes. (**A**) Identified genes are grouped into six categories. (**B**) In follicle cells and S2 cells, the 11 genes, *br*, *Prosα7*, *nej*, *Uba1*, *Not1*, *Ca-P60A*, *N*, *Ssrp*, *drk*, *mam* and *mop* were shared. (**C**) Two genes, *Ssrp* and *mop*, are shared by the follicle cells, wings and eyes. Between the follicle cells and wings are four common genes, *N*, *mam*, *Not1* and *Ca-P60A*. The follicle cells and eyes share two common genes, *Rab11* and *shi*. (**D**) *Ssrp* is the only gene to overlap among the follicle cells, Kc167 cells and S2 cells. *Jub* and *Rpt4* were shared by follicle cells and Kc167 cells.

In order to investigate potential general features, we analyzed the frequency of gene ontology (GO) annotations of the 33 identified genes against the *Drosophila* genome background. GO annotations include three categories: biological process, molecular function and cellular component. The top 20 enriched terms were selected in each category (Supplemental Table 2). The top four enriched terms of biological process are general terms, including regulation of cellular progress, regulation of biological progress, biological regulation and regulation of gene expression. Regulation of metabolic process and cell cycle comes next, suggesting metabolic pathways and cell cycle regulators are utilized by Notch signaling to regulate follicle cell differentiation and cell cycle switches. The top five enriched terms of molecular function all involve binding, including protein binding, binding, ion binding, translation initiation factor binding and nucleotide binding, indicating the majority of the identified Notch signaling components (25/33) either form protein-protein complexes to initiate/regulate translation, or directly/indirectly bind to nucleotides to control gene expression, or act through ion channels to regulate signaling transduction. The top four enriched terms of cellular component are cell, cell part, intracellular part and intracellular, confirming Notch signaling activities mainly happen within cells. The fifth enriched term is cytoskeleton, implying Notch relies on cytoskeleton proteins for proper signaling transduction. Due to the small sample size (33 genes), GO annotations give us very broad description. By extracting the detailed information of the 33 identified genes from FlyBase, we assigned most of these genes into six categories, including Notch and associated factors, trafficking components, genes associated with protein degradation, the Hippo pathway components, cytoskeleton associated genes, and genes related to transcription or translation (Fig. 3A, Table 1).

The Merdes Lab also carried out an extensive RNAi screen by treating *Drosophila* S2 cells with dsRNA and identified 900 Notch regulator candidates^19^. Further *in vivo* experiments in the wing and eye confirmed 333 of 501 tested candidates as Notch regulators. We extracted and organized their raw data, including the 900 candidate genes tested in S2 cells, 268 in wing disc and adult wing, 175 in eye disc and adult eye (Supplemental Table 3). Venn diagrams (Fig. 3B) show 11 of 33 genes from our screen were shared by follicle cells and S2 cells, including *br*, *Prosα7*, *nej*, *Uba1*, *Not1*, *Ca-P60A*, *N*, *Ssrp*, *drk*, *mam* and *mop*. We further compared candidate genes between the follicle cells, wing and eye (Fig. 3C). Only two genes, *Ssrp* and *mop*,were shared by all three tissues (Fig. 3C). The only shared genes between the follicle cells and wing were *N*, *mam*, *Not1* and *Ca-P60A* (Fig. 3C). It should be noted that the raw data in the eye added a few extra positive genes and only tested ectopic upregulation of Notch^19^, suggesting we might be able to consider *Ssrp*, *mop*, *N*, *mam*, *Not1* and *Ca-P60A* as common Notch signaling associated genes across tissues. The two conserved genes in the list, *N* and *mam*, further justified our speculation. There were also two genes shared by the follicle cells and eye, *Rab11* and *shi* (Fig. 3C). *shi* has been known to control vesicular trafficking via Rab11-positive endosomes^20^, indicating the endocytic trafficking pathway is critical for Notch signaling in both follicular epithelium and eye development.

In the DeDecker Lab, another independent genome-wide RNAi screen was done to identify Notch modulators^21^. *Drosophila* Kc167 cells were treated with dsRNA and 399 putative Notch modifiers were identified. After removing two redundant genes, we organized their raw data and extracted 397 candidate genes tested in Kc167 cells (Supplemental Table 3). Mourikis *et al.* tested *N*, *mam*, and *Su(H)* as positive controls, though they did not include them in the list of candidate genes. This partially explains the poor overlap percentage between our results, Kc167 cell data and S2 cell data. Only *Ssrp* overlaps in the three sets of data. *Jub* and *Rpt4* were shared by the follicle cells and Kc167 cells (Fig. 3D).

In addition, the Knoblich Lab applied *in vivo* genome-wide RNAi screen combined with a tissue-specific Gal4 driver to knock down genes, and examined the effects in external sensory organ morphology^22^. From their results and others, 177 candidate genes involved in Notch signaling were proposed to regulate external sensory organ development (Supplemental Table 3). We compared our 33 identified genes with the 177 candidate genes, and found 6 genes in common, including *N, mam,α-Adaptin, cdc2, sec6,* and *Smr.*

In most *in vivo* genome-wide RNAi screen projects, scientists always use tissue-specific Gal4 driver to knock down the expression of genes, and examine the effect in adult flies. The advantages of this strategy include fast screening and easy identification. However, this process is limited in that knockdown of some essential genes might cause lethality of adult flies, or that subtle effects might go unnoticed. In our study, we applied flip-out system to control the timing of induction of RNAi effect, and compare RNAi knockdown clonal cells with neighboring wildtype cells to identify these subtle changes. For instance, the genes *Ca-P60A, br, eIF3-S9, exba, MED15, mop, nej, Not1, Rab11, Rop, Rpt4, shi, Spt6, Ssrp, T-cp1* that we found regulate Notch signaling could not be analyzed by Mummery-Widmer *et al.* studies because of lethality at the pupal stage^22^. This example emphasizes the importance of spatiotemporal inactivation of genes, and studies like ours could complement previous *in vivo* genome-wide RNAi screen projects to identify novel genes involved in Notch signaling.

### Anterior follicle cells undergo the M/E switch earlier than posterior follicle cells

Throughout the screen project, we observed some other interesting phenomena, such as earlier endocycling in anterior follicle cells as opposed to that of posterior follicle cells. During the M/E switch, wild-type anterior follicle cells showed decreased Cut expression, while the same posterior follicle cells still possessed strong expression (Fig. 2D”). Furthermore, during the change to endocycle and in accordance with Cut patterns, anterior wild-type follicle cells showed weak Hnt and Br expression first, while posterior follicle cells had no expression (Fig. 2B”). Consistent with our findings, further analysis of wild-type egg chambers showed early Cut downregulation, Hnt and Br upregulation in the anterior follicle cells (Supplemental Fig. 1), suggesting anterior follicle cells undergo the M/E switch earlier.

### The insulin-PI3K pathway interacts with Notch signaling during the M/E switch

**Figure 4:**
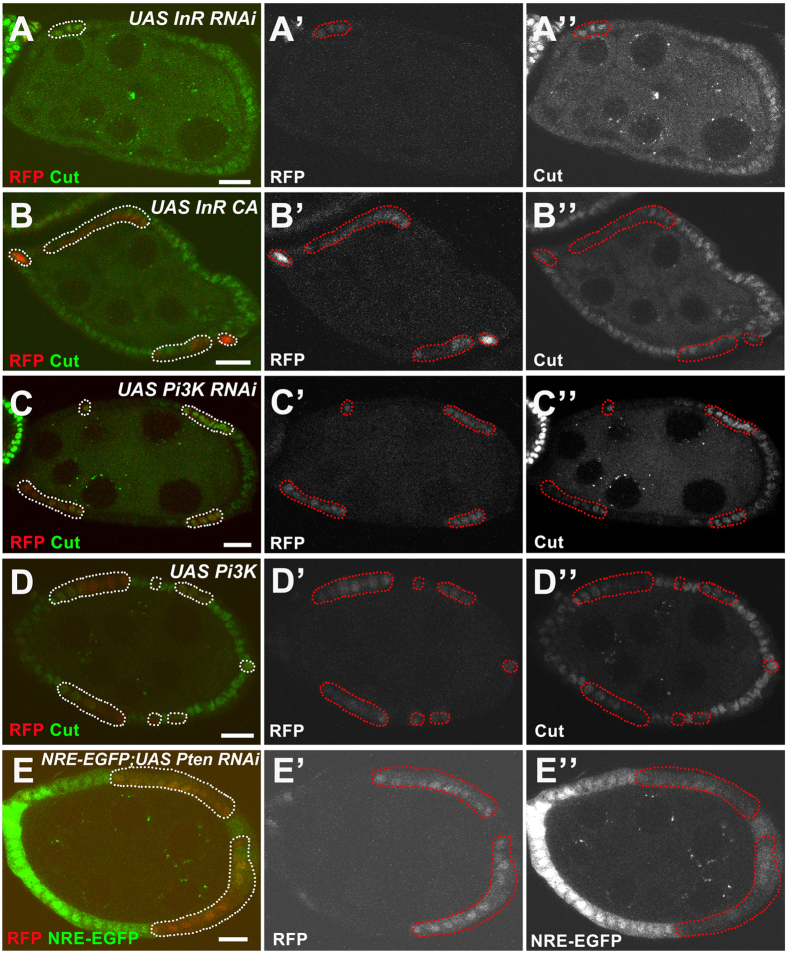
The insulin-PI3K pathway interacts with Notch signaling. (**A**-**A”**) *InR* RNAi follicle cells (marked by presence of RFP and outlined with dotted lines) showed upregulated Cut expression (green in **A**, white in **A”**). (**B**-**B”**) Overexpressing the constitutively active form of InR (*InR CA*) in follicle cells suppressed Cut expression (green in **B**, white in **B”**). (**C**-**C”**) *PI3K* RNAi expressing follicle cells showed upregulated Cut expression (green in **C**, white in **C”**). (**D**-**D”**) Overexpressing wild-type PI3K in follicle cells reduced Cut expression (green in **D**, white in **D”**). (**E**-**E”**) *Pten* RNAi expressing follicle cells showed reduced NRE-EGFP level (green in **E**, white in **E”**). Anterior is to the left. Clone regions marked by presence of RFP and outlined with dotted lines. Bars, 10 μm.

From the screen, we identified *Pten*, which encodes *Drosophila* PTEN (phosphatase and tensin homolog). *Pten*, along with *exba*, were the only two identified genes to display premature endocycle entry when their gene expression was knocked down. Pten is well known as a negative regulator of the insulin-PI3K pathway^23^. The insulin-PI3K pathway is highly conserved across species and is important for nutrition-dependent growth and cell size regulation^24^. In *Drosophila*, when the insulin receptor (InR) is bound by insulin-like peptides, its substrate, Chico, will be phosphorylated and activated, thus stimulating phosphoinositide 3-kinase (PI3K) to convert phosphatidylinositol-3,4-bis-phosphate (PIP2) lipids into the phosphatidylinositol-3,4,5-triphosphate (PIP3). This PIP2-PIP3 conversion is reversible by the lipid PTEN^25^. We wondered whether the whole insulin-PI3K pathway participates in regulating Cut expression during the M/E switch. As such, we tested two important genes in the insulin-PI3K pathway, *InR* and *PI3K*. In contrast to Pten, both InR and PI3K are positive regulators of the insulin-PI3K pathway. During the M/E switch, knockdown of InR led to upregulated Cut, while overexpression of the constitutively active form of InR suppressed Cut (Fig. 4A,B). Similarly, PI3K reduction led to upregulated Cut, whereas increased wild-type PI3K resulted in lower levels of Cut (Fig. 4C,D). These phenotypes (Figs 2) were only observed near the M/E switch, indicating the insulin-PI3K pathway is important for the precise transition into the endocycle and follicle cell differentiation.

A recent report describes how Cut, Hnt and the Notch activity reporter, Notch response element (NRE), briefly overlap between stage 6 and 7, which is termed the M/E switch (MES) stage^26^. During this short but special MES stage, Cut may act in a feedback loop to positively regulate Notch. Starvation was able to pause the MES stage. Knockdown of InR had been shown to elevate NRE-lacZ expression^26^. Here we further report that knockdown of the negative regulator Pten repressed the NRE-EGFP reporter^19^ (Fig. 4E), suggesting the insulin-PI3K pathway is essential for the transient MES through Cut-maintained Notch activity. We studied multiple key components of insulin-PI3K signaling, and linked the signaling with Notch during the normal M/E transitional stages.

### Nejire is involved in Notch signaling to regulate the M/E switch

Cofactors affect signal transduction cascades through modulation of transcriptional repression/activation. Many cofactors are involved in remodeling of chromatin through histone modification, which involves packing/unpacking DNA into higher-order chromatin structure. Cofactors also play critical roles in maintaining or altering the chromatin structure around the enhancer/promoter regions to regulate gene expression^27^. Here, we focused on the coactivator called Nej, also known as dCBP, which was identified from the screen. In *Drosophila,* Nej contains multiple domains as a member of the CBP/p300 family, one of which is the histone acetyltransferase domain. CBP/p300 family members can serve as a protein scaffold to recruit different sequence-specific transcription factors to form a multicomponent transcriptional regulatory complex. They can acetylate nucleosomal histones to remodel chromatin structure and nonhistone proteins, such as p53 tumor suppressor^28^. Nej has been identified as a transcriptional coactivator involved in a number of signaling pathways, such as the Wingless, Hedgehog, and Dpp pathways^29^,^30^,^31^,^32^,^33^, although its involvement in the Notch pathway is not well understood. An *in vitro* assay showed MAML1, a mammalian Mam homolog, can potentiate NICD-mediated transcription on naked DNA templates, but not on chromatin templates. With the help of p300, a mammalian Nej homolog that can interact with NICD, MAML1 can potentiate transcription on chromatin templates^34^, suggesting a potential role of Nej in the Notch pathway. Nej was identified from our screen, indicating a role as a coactivator functioning in the follicle cells to regulate the M/E switch.

**Figure 5:**
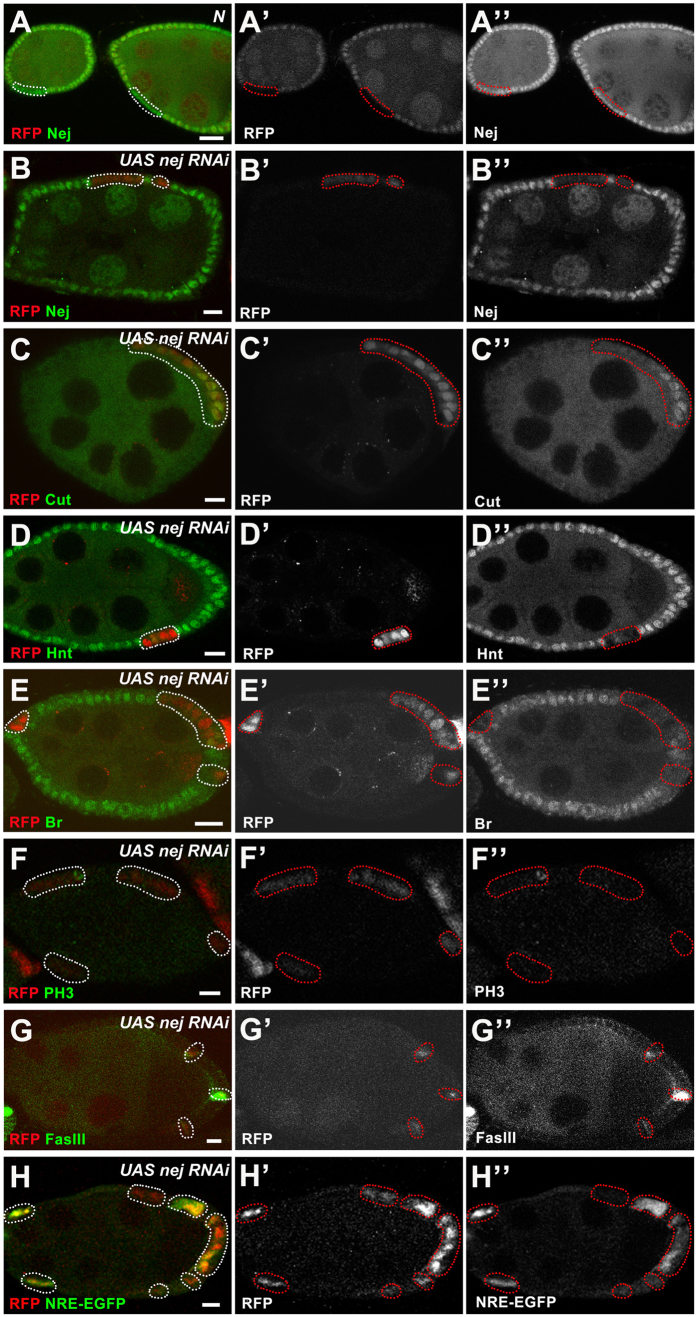
Nejire interacts with Notch signaling to regulate the ME switch and maintain MES. (**A**-**A”**) Nej was ubiquitously expressed in follicle cells during oogenesis. *N*^*55e11*^ follicle-cell FLP/FRT clones (marked by the absence of RFP, red in **A**, white in **A’**; outlined) still retained expression of Nej (green in **A**, white in **A”**). (**B**-**H**) Follicle cells expressing *nej* RNAi. Clone region marked by presence of RFP and outlined with dotted lines. (**B**-**B”**) *nej* RNAi follicle cells in a stage-7 egg chamber successfully abolished Nej expression (green in **B**, white in **B”**). (**C**-**E”**) Upregulation of Cut (green in **C**, white in **C”**), and downregulation of Hnt (green in **D**, white in **D”**) and Br (green in **E**, white in **E”**) were also observed in *nej* RNAi follicle cells. (**F**-**F”**) Follicle cells expressing *nej* RNAi showed sporadic PH3 staining (green in **F**, white in **F”**). (**G**-**G”**) *nej* RNAi expressing follicle cells retained FasIII expression (green in **G**, white in **G”**) during midoogenesis. (**H**-**H”**) *nej* RNAi expressing follicle cells showed increased NRE-EGFP level (green in **H**, white in **H”**). Anterior is to the left. Bars, 10 μm.

In this study, we used *Drosophila* follicle cells as a model system to explore the relationship between Nej and Notch signaling *in vivo*. Nej was detected ubiquitously in follicle cells during oogenesis and its expression was still present in *N*^*55e11*^ (loss-of-function allele of *N*) follicle cell clones (Fig. 5A), further suggesting Nej is not a Notch downstream target, rather a ubiquitously present coactivator for proper developmental events. *nej* RNAi successfully abolishes Nej expression (Fig. 5B), and thus serves as a reliable tool to study loss-of-function during *Drosophila* oogenesis. Because *nej* RNAi lines (BL#27724, #31728, #37489) showed consistent phenotypes, one *nej* RNAi line (BL#37489) was randomly selected for use in future experiments. As described in Table 1, when *nej* RNAi was introduced, the expression of Cut was upregulated during midoogenesis (Fig. 5C), while Hnt and Br were downregulated during midoogenesis (Fig. 5D, E). Phospho-histone 3 (PH3), a mitotic marker, is present in the wild-type follicle cells in an oscillating pattern during the mitotic cycle, then disappears after the follicle cells enter the endocycle^2^. In Nej knockdown egg chambers, we were still able to detect that 14% of stage 7 mosaic egg chambers showed PH3-positive follicle cells (n = 43; Fig. 5F), indicating *nej* RNAi expressing follicle cells fail to properly transition from the mitotic cycle into the endocycle. In addition, mainbody follicle cells are not fully differentiated in early oogenesis and can be labeled by immature cell fate marker Fasciclin III (FasIII). FasIII is undetectable in endocycling (“mature”) mainbody follicle cells beyond stage 7^5^,^16^, however, *nej* RNAi expressing follicle cells still retained FasIII expression during midoogenesis (Fig. 5G), implying these cells maintain an undifferentiated cell fate. All these results (Fig. 5C–G) confirmed Nej is required for proper follicle cell differentiation and M/E switching. We further tested whether *nej* is involved in Notch signaling, and intriguingly, knockdown of *nej* does not lead to suppressed Notch activity reporters NRE-EGFP. Instead, NRE-EGFP was upregulated (Fig. 5H), whereby we speculate that the upregulation of NRE-EGFP reporter is due to the upregulated Cut at the MES stage. Meanwhile, we did not rule out other possibilities. For example, *nej* might be required by other genes or signaling pathways, which indirectly regulate the M/E switch. Our results for the first time link Notch and *nej in vivo* in *Drosophila*.

## Discussion

Notch signaling in humans has a variety of important roles in cell differentiation and stem cell formation, and has been associated with genetic diseases and cancers^35^,^36^. To better understand the contribution of Notch signaling to normal development and to be able to treat the diseases caused by aberrant Notch activity, a systematic identification and characterization of its components and connections to other pathways is necessary and informative.

From this large-scale *in vivo* RNAi screen, we were able to identify genes involved in follicle cell differentiation and cell cycle switches mediated by Notch signaling. Identifying each of these genes is tremendously important to gain insight into the proliferation and differentiation of cells, and further understand how they are related to diseases, including cancers. Among the 33 identified genes, *N* and *mam* are well known as conserved components of the Notch pathway^37^,^38^. Our analysis (Fig. 3C) highlights the genes *Ssrp*, *mop*, *Not1* and *Ca-P60A* as potential candidates associated with Notch signaling across tissues. *Ssrp* as yet has not been linked to Notch signaling, however, it is implicated in two previously mentioned RNAi screen papers and by our results as well (Fig. 3B–D). *Ssrp* shows nucleosomal DNA binding ability^39^,^40^, indicating that it could be a critical component of chromatin remodeling complexes regulating Notch signaling. Our finding that Ssrp regulates epithelial follicle cell differentiation and cell cycle transition during *Drosophila* oogenesis is consistent with a previous report showing that Ssrp1a, the zebrafish Ssrp homolog, controls development of tissues like the liver and eye by promoting cell proliferation and differentiation^41^. *mop* is a very interesting gene, encoding a protein-tyrosine phosphatase that contains BRO1 domain and ALIX V-shaped domain. Mop physically interacts with multiple signaling pathway components, including the endocytic pathway components Hrs^42^, Rab4, Rab5^43^ and Vps28^44^, the Notch pathway component Su(dx)^44^, the Hippo pathway component Yki^45^, and Drk from our screen^44^. *Not1* is a key component of the CCR4-NOT complex, which is responsible for catalyzing mRNA deadenylation^46^. *Ca-P60A* positively regulates calcium-transporting ATPase activity for proper intracellular trafficking of the Notch receptor^47^. The commonality of these genes across tissues suggests that chromatin remodeling, the endocytic pathway, and gene silencing all play important roles in regulating Notch signaling.

We reference and summarize detailed information of all the identified genes in Table 1. In general, most of these genes fall into six categories (Fig. 3A). Group I includes Notch and associated factors, containing Notch signaling conserved components *N* and *mam*, NICD-Su(H)-Mam complex-associated factor *e(y)1*^48^, cofactors *nej*^34^ and *Smr*^49^, and the Notch downstream target *br*^18^. We also speculate zinc finger protein CG9797 might be a downstream target of Notch, similar to Cut, Hnt, and Br. Mop is physically involved with Su(dx), acting as a potential Notch-associated factor. Group II contains trafficking components. The endocytic pathway includes *α-Adaptin*^50^, *Ca-P60A*^47^, *Hsc70-4*^51^, *rab11* and *shi*^20^. Binding of α-Adaptin by Numb is required for targeting Notch for degradation^50^. *Ca-P60A* and *Hsc70-4* regulate proper intracellular trafficking of the Notch receptor^47^,^51^. *rab11* and *shi* control Notch trafficking via endosomes^20^. Exocytosis requires *rop*^52^and *sec6*^53^. Specifically, *sec6* is a key component of exocysts^53^. Group III are genes associated with protein degradation, composed of proteasome subunit *Prosα7,* proteasome-associated regulatory complex subunit *Rpt4*^54^, and E1 ubiquitin-activating enzyme *Uba1*^55^. Group IV is related to the Hippo pathway, which promotes Notch signaling in the posterior follicle cells during the M/E switch^56^, and contains *ex*, *mats* and *jub*^57^. The cytoskeleton and its structure has profound impact on signaling transduction^58^, and its associated genes are in Group V, including *drk*^59^, *me31B*, *Rpt4* and *T-cp1*^60^. Group VI generally includes genes related to transcription or translation, such as *eIF3-S9*^61^, *fs(1)K10*^62^, *exba*^63^, *Not1*^46^*,Spt6*^64^, and *MED15*^65^. *eIF3-S9* and *exba* are eukaryotic translation initiation factor subunits^61^,^63^. *fs(1)K10* negatively regulates translation^62^. *Not1* is responsible for catalyzing mRNA deadenylation^46^. *Spt6* is a transcription elongation factor^64^*. MED15* is a subunit of the Mediator complex, which is required for regulating RNA polymerase II (pol II) transcripts^65^. Other subunits, including *MED6, MED7, MED8, MED11, MED14, MED17, MED20, MED23, MED25, MED26, MED27, MED30, MED31*, have been identified as Notch modulators in previous screen projects, highlighting the importance of the Mediator complex in regulating Notch signaling^66^. While *Ssrp* and *cdc2* are not grouped into the six categories, they are equally important. *Ssrp* and *cdc2* indicate the critical roles of chromatin remodeling and cell cycle regulation in modulating Notch, respectively^39^,^40^.

This screen advances our knowledge of the M/E switch and Notch signaling network. Previously, we generally considered follicle cells to be uniform until the M/E switch^67^. However, our findings suggest the anterior follicle cells enter endocycle earlier, indicating that a symmetry-breaking process happens before the M/E switch. Through the gene validation process, we discovered that both the insulin-PI3K pathway and *nej* participate in the proper M/E switch, and are especially important for the MES. During the MES, Cut, Hnt, and Notch activity reporters all coexist, with Cut enhancing Notch signaling instead of suppressing it. However, the exact method whereby this transient stage is maintained and its biological importance are still open questions and await further investigation. Our screen findings connect the whole insulin-PI3K pathway and a cofactor *nej* with the MES stage, opening the gate to further elucidation of its molecular mechanisms and biological implications.

## Materials and Methods

### Fly Stocks and Genetics

The following fly lines were used: RNAi flies used for the screen were from the *Drosophila*RNAi Screening Center, distributed by Bloomington *Drosophila* Stock Center (Supplemental 1), *UAS-InRRNAi*(BL#31594), *UAS-InR CA* (BL#8248), *UAS-PI3K RNAi*(BL#27690), *UAS- PI3K* (BL#8286), *NRE-EGFP*(BL#30727^19^), *N*^*55e11*^ (amorphic allele^5^,^16^), *br*^*npr3*^(amorphic allele^18^), *PBac{SAstopDsRed}LL08100* (Kyoto DGRC#142194), *uba1*^*s3484*^ (Kyoto DGRC#114337), *ex*^*e1*^ (amorphic allele^56^)and *w*^*1118*^ was used as a wild-type control.

To generate mosaic egg chambers expressing UAS constructs, the flip-out Gal4^68^ stock *hsFLP; actin*<*CD2<Gal4,UAS-RFP/TM3,Sb* was applied. Occasionally, *hsFLP; actin<CD2<Gal4,UAS-GFP* was used as well. *hsFLP; actin<CD2<Gal4,UAS-RFP/TM3,Sb* virgin female flies were selected to cross with *UAS-RNAi* males. After two weeks culture under 25 °C, F1 generation adult female flies were collected to undergo 30 minutes heat shock at 37 °C for two consecutive days, in order to create follicle cell clones. After heat shock, all flies were maintained in fresh food vials with wet yeast paste for two days before dissection. For FLP/FRT clone induction^69^,^70^, previously described procedures were followed^18^.

### Immunohistochemistry and Image Analysis

Immunohistochemistry and image acquisition were carried out as previously described (Sun and Deng, 2005). The following primary antibodies were used: mouse anti-Cut (2B10) 1:15, mouse anti-Br-Core (25E9) 1:30, mouse anti-Hnt (1G9) 1:15, mouse anti-FasIII (7G10) 1:15 (Development Studies Hybridoma Bank, USA), rabbit anti-PH3 1:200 (Upstate Biotechnology, NY, USA), guinea pig anti-dCBP (Nej) 1:1000 (provided by Mattias Mannervik, Stockholm University, Stockholm, Sweden)^31^. Corresponding Alexa Fluor secondary antibodies (1:400; Invitrogen) were selected according to primary antibodies. DAPI (Invitrogen) was applied for nuclei staining. Images were acquired with a Zeiss LSM 510 confocal microscope and processed in Photoshop and Image J.

### Gene Ontology (GO) term enrichment analysis and Venn diagram generation

All identified genes from the RNAi screen were analyzed for an enrichment of GO terms, including biological process, molecular function and cellular component, via the GO tool AmiGO2 (http://amigo.geneontology.org/amigo). Venn diagrams were initially created by the tool Venn diagram generator (http://www.bioinformatics.lu/venn.php), then reproduced and processed in Microsoft Paint and Photoshop. Detailed gene information was extracted from FlyBase (http://flybase.org).

## Additional Information

**How to cite this article**: Jia, D. *et al.* A large-scale *in vivo* RNAi screen to identify genes involved in Notch-mediated follicle cell differentiation and cell cycle switches. *Sci. Rep.* **5**, 12328; doi: 10.1038/srep12328 (2015).

## Supplementary Material

Supplementary Information

## Figures and Tables

**Table 1:** List of identified genes from the RNAi screen.

Stock	CG#	Gene Name	Screen phenotype	Functions related to Notch	Group (Fig. 3A)	References linked to Notch
BL#32866	CG4260	*alpha-Adaptin (α-Adaptin)*	Cut Up in midoogenesis	endocytic pathway component	II	50
BL#27272, *br*^*npr3*^	CG11491	*broad (br)*	Cut Up in midoogenesis	Notch target	I	18
BL#25928, NIG#3725R-2	CG3725	*Calcium ATPase at 60A (Ca-P60A)*	Cut Up in midoogenesis	calcium-transporting ATPase activity for Notch endocytosis	II	19,47
BL#28368, NIG#HMS01531	CG5363	*cdc2*	Cut Up in midoogenesis	Cyclin-dependent kinase	–	22
BL#27563, BL#31494	CG6033	*downstream of receptor kinase (drk)*	Cut Up in midoogenesis	actin filament organization	V	19
BL#32345, NIG#6474R-1	CG6474	*enhancer of yellow 1 (e(y)1)*	Cut Up in midoogenesis	NICD-Su(H)-Mam complex-associated factor	I	48
BL#32880, NIG#4878R-3	CG4878	*eIF3-S9*	Cut Up in midoogenesis	translation initiation factor	VI	*
BL#28703, *ex*^*e1*^	CG4114	*expanded (ex)*	Cut Up in midoogenesis	Hippo pathway component	IV	56
BL#33008, BL#33630	CG3218	*female sterile (1) K10 (fs(1)K10)*	Cut Up in midoogenesis	EGFR signaling; negative regulation of translation	VI	*
BL#28709, BL#34836	CG4264	*Heat shock protein cognate 4 (Hsc70-4)*	Cut Up in midoogenesis	Notch receptor subcellular trafficking	II	51
BL#32923, BL#41938	CG11063	*Ajuba LIM protein (jub)*	Cut Up in midoogenesis	Hippo pathway- associated	IV	19, 56
BL#27248, NIG#2922R-2	CG2922	*exba*	Cut Down in early oogenesis	translation initiation factor binding	VI	*
BL#32858, BL#41937	CG9797	*CG9797*	Cut Up in midoogenesis	C2H2 Zinc finger protein	I	*
BL#28046	CG8118	*mastermind (mam)*	Cut Up in midoogenesis	Notch pathway component	I	38
BL#29567, BL#34959	CG13852	*mob as tumor suppressor (mats)*	Cut Up in midoogenesis	Hippo pathway component	IV	56
BL#28566, BL#33675	CG4916	*maternal expression at 31B (me31B)*	Cut Up in midoogenesis	microtubule associated complex; gene silencing by miRNA	V	*
BL#32517, NIG#4184R-3	CG4184	*Mediator complex subunit 15 (MED15)*	Cut Up in midoogenesis	Mediator (MED) complex	VI	*
BL#32916, BL#34085	CG9311	*myopic (mop)*	Cut Up in midoogenesis	the endocytic pathway, Notch pathway, Hippo pathway	I	19, 44
BL#33611, BL#31180, BL#27988, BL#28981	CG3936	*Notch (N)*	Cut Up in midoogenesis	Notch pathway component	I	37,38
BL#27724, BL#31728, BL#37489	CG15319	*nejire (nej)*	Cut Up in midoogenesis	Notch coactivator	I	34
BL#32836, *PBac{SAstopDsRed}LL08100*	CG34407	*Not1*	Cut Up in midoogenesis	CCR4-NOT complex is the major enzyme catalyzing mRNA deadenylation with Caf1	VI	19
BL#33660, NIG#1519R-2	CG1519	*Proteasome α7 subunit (Prosα7)*	Cut Up in midoogenesis	Proteasome subunit for protein degradation	III	*
BL#25967, BL#25841, BL#33643	CG5671	*Pten*	Cut Down in early oogenesis	insulin-PI3K pathway component	II	71
BL#27730,NIG#5771R-2	CG5771	*Rab11*	Cut Up in midoogenesis	endocytic pathway component	II	20
BL#28929	CG15811	*Ras opposite (Rop)*	Cut Up in midoogenesis	exocytosis	II	*
BL#32874	CG3455	*Regulatory particle triple-A ATPase 4 (Rpt4)*	Cut Up in midoogenesis	microtubule associated complex, proteasome-mediated ubiquitin-dependent protein catabolic process	III	21
BL#27314, NIG#HMJ21305	CG5341	*sec6*	Cut Up in midoogenesis	positive regulation of exocytosis	II	53
BL#28513, BL#36921	CG18102	*shi*	Cut Up in midoogenesis	endocytic pathway component	II	19, 20
BL#27068	CG4013	*Smr*	Cut Up in midoogenesis	Notch cofactor	I	49
BL#32373, NIG#12225R-1	CG12225	*Spt6*	Cut Up in midoogenesis	positive regulation of transcription elongation	VI	72
BL#26222, NIG#4817R-2	CG4817	*Ssrp*	Cut Up in midoogenesis	chromatin remodeling	–	19, 21
BL#32854, NIG#5374R-2	CG5374	*T-cp1*	Cut Up in midoogenesis	ATP binding, microtubule	V	*
BL#25957, *uba1*^*s3484*^	CG1782	*Uba1*	Cut Up in midoogenesis	E1 ubiquitin-activating enzyme for protein degradation	III	55

RNAi flies are either from Bloomington (BL) or NIG stock center. Nine lines with (*) denote novel links identified in the screen.
