# Supplementary material for: A large-scale in vivo RNAi screen to identify genes involved in Notch-mediated follicle cell differentiation and cell cycle switches
Source: Sci Rep. 2015 Jul 24;5:12328. doi: 10.1038/srep12328 (PMC4513280; doi:10.1038/srep12328)
Supplement: Supplementary Information [file srep12328-s1.pdf]

A large-scale *in vivo* RNAi screen to identify genes involved in Notch-mediated follicle cell differentiation and cell cycle switches

Dongyu Jia, Muhammed Soylemez,Gabriel Calvin, Randy Bornmann,Jamal Bryant, Cameron Hanna ,Yi-Chun Huang, Wu-Min Deng

Supplemental Figure 1. Early M/E switch entry in anterior follicle cells. Wild-type egg chambers showed early Cut downregulation (A), Hnt (B) and Br (C) upregulation in the anterior follicle cells. Anterior is to the left. Bars, 10 µm.

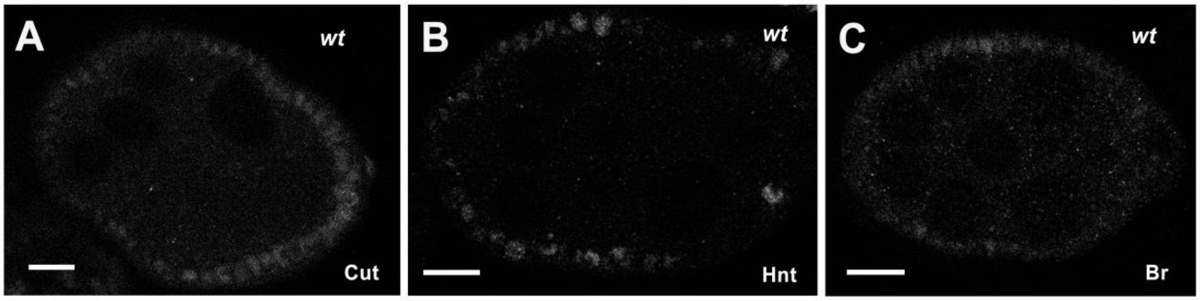

Supplemental Table 1. List of genes tested by the RNAi screen.

BL#	CG#	Gene Name	Vector
26763	CG17172	(A+T)-stretch binding protein	VALIUM10
28327	CG17870	14-3-3zeta	VALIUM10
32887	CG8947	26-29kD-proteinase	VALIUM20
27737	CG33323	48 related 1	VALIUM10
28697	CG5952	48 related 2	VALIUM10
25974	CG6913	48 related 3	VALIUM10
33418	CG15113	5-hydroxytryptamine (serotonin) receptor 1B	VALIUM20
32471	CG12073	5-hydroxytryptamine (serotonin) receptor 7	VALIUM20
29620	CG31110	5PtaseI	VALIUM10
27989	CG1168	7B2	VALIUM10
26741	CG11405	A3-3	VALIUM10
28577	CG3705	aay	VALIUM10
28739	CG10325	abdominal A	VALIUM10
26746	CG11648	Abdominal B	VALIUM10

28325	CG4032	Abl tyrosine kinase	VALIUM10
29335	CG9151	abnormal chemosensory jump 6	VALIUM10
28741	CG6875	abnormal spindle	VALIUM10
33712	CG2210	abnormal wing discs	VALIUM20
29407	CG4807	abrupt	VALIUM10
32378	CG43860	abrupt	VALIUM20
33705	CG8887	absent, small, or homeotic discs 1	VALIUM20
28626	CG1506	Ac3	VALIUM10
25998	CG17673	Accessory gland peptide 70A	VALIUM10
25958	CG17907	Acetylcholine esterase	VALIUM10
29586	CG3796	achaete	VALIUM10
27652	CG14992	Ack	VALIUM10
27705	CG9901	Actin-related protein 14D	VALIUM10
32921	CG7558	Actin-related protein 3	VALIUM20
33009	CG7940	Actin-related protein 5	VALIUM20
26210	CG30420	Activating transcription factor-2	VALIUM10
29597	CG11062	Activin-beta	VALIUM10
32866	CG4260	Adaptor Protein complex 2, α subunit	VALIUM20
26015	CG11994	Adenosine deaminase	VALIUM10
28311	CG12598	Adenosine deaminase acting on RNA	VALIUM10
27536	CG9753	Adenosine receptor	VALIUM10
28680	CG15845	Adh transcription factor 1	VALIUM10
27031	CG1171	Adipokinetic hormone-like	VALIUM10
27268	CG11027	ADP ribosylation factor 102F	VALIUM10
27261	CG8156	ADP ribosylation factor 51F	VALIUM10
29538	CG8385	ADP ribosylation factor 79F	VALIUM10
29588	CG7435	ADP ribosylation factor 84F	VALIUM10
32923	CG11063	Ajuba LIM protein	VALIUM20
28532	CG13388	Akap200	VALIUM10
28627	CG3481	Alcohol dehydrogenase	VALIUM10
26301	CG6058	Aldolase	VALIUM10
33417	CG12876	ALG-2 interacting protein X	VALIUM20
33429	CG8057	alicorn	VALIUM20
28908	CG9556	alien	VALIUM10
27518	CG8250	Alk	VALIUM10
28740	CG1462	Alkaline phosphatase 4	VALIUM10
33673	CG1462	Alkaline phosphatase 4	VALIUM20
25866	CG13633	Allatostatin	VALIUM10
25868	CG14919	Allatostatin C	VALIUM10
27506	CG7285	allatostatin C receptor 1	VALIUM10
25940	CG13702	allatostatin C receptor 2	VALIUM10
27280	CG2872	Allatostatin Receptor	VALIUM10
25935	CG10001	Allatostatin Receptor 2	VALIUM10
28535	CG16827	alphaPS4	VALIUM10
28578	CG8561	Als	VALIUM10
33416	CG2198	Amalgam	VALIUM20
25797	CG11937	amnesiac	VALIUM10
28583	CG6438	amon	VALIUM10

28048	CG8604	Amphiphysin	VALIUM10
27515	CG8084	anachronism	VALIUM10
29438	CG34416	Ank2	VALIUM10
33414	CG42734	Ankyrin 2	VALIUM20
27675	CG1028	Antennapedia	VALIUM10
26759	CG3166	anterior open	VALIUM10
28972	CG12276	Aos1	VALIUM10
27533	CG9113	AP-1γ	VALIUM10
27322	CG6056	AP-2σ	VALIUM10
27534	CG9388	AP-47	VALIUM10
28040	CG7057	AP-50	VALIUM10
28582	CG1451	Apc	VALIUM10
28585	CG6193	Apc2	VALIUM10
26236	CG5393	apontic	VALIUM10
26024	CG1200	APP-like protein interacting protein 1	VALIUM10
26748	CG8376	apterous	VALIUM10
27060	CG10571	araucan	VALIUM10
25954	CG12505	Arc1	VALIUM10
28011	CG10954	Arc-p34	VALIUM10
27052	CG6025	Arflike at 72A	VALIUM10
33727	CG6671	Argonaute-1	VALIUM20
28383	CG4531	argos	VALIUM10
29416	CG5659	ariadne	VALIUM10
26747	CG3935	aristaless	VALIUM10
27044	CG4560	Arpc3A	VALIUM10
27528	CG8936	Arpc3B	VALIUM10
32844	CG7843	Ars2	VALIUM20
28317	CG10687	Asparaginyl-tRNA synthetase	VALIUM10
32890	CG10414	ATAC complex component 2	VALIUM20
26316	CG7508	atonal	VALIUM10
28062	CG8189	ATP synthase, subunit b	VALIUM10
33740	CG6030	ATP synthase, subunit D	VALIUM20
28056	CG11154	ATP synthase-beta	VALIUM10
28723	CG7610	ATP synthase-gamma chain	VALIUM10
25946	CG10261	atypical protein kinase C	VALIUM10
33728	CG6137	aubergine	VALIUM20
26731	CG10967	Autophagy-specific gene 1	VALIUM10
27552	CG10861	Autophagy-specific gene 12	VALIUM10
28061	CG7986	Autophagy-specific gene 18	VALIUM10
27706	CG1241	Autophagy-specific gene 2	VALIUM10
28367	CG4428	Autophagy-specific gene 4	VALIUM10
27551	CG1643	Autophagy-specific gene 5	VALIUM10
28060	CG5429	Autophagy-specific gene 6	VALIUM10
27707	CG5489	Autophagy-specific gene 7	VALIUM10
28989	CG32672	Autophagy-specific gene 8a	VALIUM10
27554	CG12334	Autophagy-specific gene 8b	VALIUM10
28055	CG3615	Autophagy-specific gene 9	VALIUM10
28509	CG1107	auxillin	VALIUM10

26230	CG1605	az2	VALIUM10
25933	CG8224	baboon	VALIUM10
33631	CG10422	bag of marbles	VALIUM20
27061	CG7902	bagpipe	VALIUM10
32977	CG5680	basket	VALIUM20
28586	CG1034	bcd	VALIUM10
29454	CG6500	Beadex	VALIUM10
28072	CG14334	beaten path IIa	VALIUM10
29607	CG15138	beat-IIIc	VALIUM10
28758	CG31298	beat-Vb	VALIUM10
28373	CG15148	beethoven	VALIUM10
28049	CG9748	belle	VALIUM10
28059	CG3612	bellwether	VALIUM10
28721	CG18319	bendless	VALIUM10
28328	CG12532	Beta Adaptin	VALIUM10
28043	CG7727	beta amyloid protein precursor-like	VALIUM10
28601	CG1762	betaInt-nu	VALIUM10
28571	CG6605	BicD	VALIUM10
28341	CG3578	bifid	VALIUM10
28372	CG1822	bifocal	VALIUM10
27691	CG4722	big brain	VALIUM10
26213	CG7959	Big brother	VALIUM10
29325	CG3350	bigmax	VALIUM10
27511	CG7811	black	VALIUM10
33667	CG10630	blanks	VALIUM20
26755	CG3411	blistered	VALIUM10
26215	CG1414	bobby sox	VALIUM10
28029	CG30498	boca	VALIUM10
27047	CG5206	bonus	VALIUM10
26308	CG3274	Brahma associated protein 170kD	VALIUM10
32503	CG4303	Brahma associated protein 60kD	VALIUM20
29409	CG13969	brain washing	VALIUM10
29336	CG10604	brain-specific homeobox	VALIUM10
28590	CG10719	brat	VALIUM10
28019	CG10542	Bre1	VALIUM10
29321	CG31256	Brf	VALIUM10
27272	CG11491	broad	VALIUM10
27074	CG10021	brother of odd with entrails limited	VALIUM10
25891	CG42344	bruchpilot	VALIUM10
25926	CG5295	brummer	VALIUM10
33421	CG31132	BRWD3	VALIUM20
27651	CG11494	BTB-protein-VII	VALIUM10
28912	CG11494	BTB-protein-VII	VALIUM10
27075	CG2932	Bteb2	VALIUM10
25791	CG8049	Btk family kinase at 29A	VALIUM10
32989	CG7581	Bub3	VALIUM20
28639	CG4501	bubblegum	VALIUM10
29608	CG8238	Buffy	VALIUM10

28322	CG5461	bunched	VALIUM10
26719	CG13419	bursicon	VALIUM10
29453	CG12653	buttonhead	VALIUM10
27649	CG7937	C15	VALIUM10
25830	CG4894	Ca2+-channel protein α1 subunit D	VALIUM10
33413	CG4894	Ca2+-channel protein α1 subunit D	VALIUM20
29575	CG6320	Ca2+-channel-protein-beta-subunit	VALIUM10
32990	CG3606	cabeza	VALIUM20
27244	CG1522	cacophony	VALIUM10
27485	CG6445	Cad74A	VALIUM10
27295	CG4509	Cad86C	VALIUM10
28716	CG6977	Cad87A	VALIUM10
29303	CG3389	Cad88C	VALIUM10
26287	CG14900	Cad89D	VALIUM10
27266	CG10244	Cad96Ca	VALIUM10
25860	CG13664	Cad96Cb	VALIUM10
27510	CG31009	Cad99C	VALIUM10
27711	CG31009	Cad99C	VALIUM10
27503	CG7100	Cadherin-N	VALIUM10
27508	CG7527	CadN2	VALIUM10
28302	CG6702	Calbindin 53E	VALIUM10
25850	CG1455	Calcineurin A1	VALIUM10
27307	CG4209	Calcineurin B	VALIUM10
27270	CG11217	Calcineurin B2	VALIUM10
25928	CG3725	Calcium ATPase at 60A	VALIUM10
27556	CG6703	Calcium/calmodulin-dependent protein kinase	VALIUM10
26726	CG1495	Calcium/calmodulin-dependent protein kinase I	VALIUM10
29401	CG18069	Calcium/calmodulin-dependent protein kinase II	VALIUM10
33594	CG12737	Calmodulin-binding protein related to a Rab3 GDP/GTP exchange protein	VALIUM20
27062	CG42332	Calmodulin-binding transcription activator	VALIUM10
29455	CG7563	Calpain-A	VALIUM10
25963	CG8107	Calpain-B	VALIUM10
25839	CG11059	calsyntenin-1	VALIUM10
27569	CG6117	cAMP-dependent protein kinase 3	VALIUM10
27308	CG42341	cAMP-dependent protein kinase R1	VALIUM10
27708	CG42341	cAMP-dependent protein kinase R1	VALIUM10
27680	CG15862	cAMP-dependent protein kinase R2	VALIUM10
33367	CG42312	canoe	VALIUM20
33648	CG7035	cap binding protein 80	VALIUM20
27275	CG14575	capa receptor	VALIUM10
28345	CG15520	capability	VALIUM10
25995	CG5067	capicua	VALIUM10
25984	CG17894	cap-n-collar	VALIUM10
32863	CG43286	cap-n-collar	VALIUM20
26298	CG17158	capping protein beta	VALIUM10
32922	CG3399	cappuccino	VALIUM20
28020	CG11282	capricious	VALIUM10
33010	CG33979	capulet	VALIUM20

29569	CG4910	Cardioacceleratory peptide	VALIUM10
27282	CG3035	carmine	VALIUM10
28337	CG13281	CAS/CSE1 segregation protein	VALIUM10
25786	CG2028	Casein kinase Iα	VALIUM10
32857	CG6703	CASK ortholog	VALIUM20
26310	CG2102	castor	VALIUM10
28978	CG1548	cathD	VALIUM10
26251	CG15899	Ca-α1T	VALIUM10
27500	CG7037	Cbl	VALIUM10
27494	CG42301	CCK-like receptor at 17D1	VALIUM10
28333	CG32540	CCK-like receptor at 17D3	VALIUM10
28368	CG5363	cdc2	VALIUM10
28952	CG10498	cdc2c	VALIUM10
28756	CG12019	Cdc37	VALIUM10
28021	CG12530	Cdc42	VALIUM10
33749	CG1458	CDGSH iron sulfur domain 2	VALIUM20
27048	CG5387	Cdk5 activator-like protein	VALIUM10
28075	CG7962	CDP diglyceride synthetase	VALIUM10
28556	CG5336	Ced-12	VALIUM10
26311	CG31258	Cenp-C	VALIUM10
28369	CG6027	center divider	VALIUM10
32388	CG10077	CG10077	VALIUM20
32981	CG10077	CG10077	VALIUM20
32431	CG10082	CG10082	VALIUM20
33717	CG10082	CG10082	VALIUM20
33701	CG10133	CG10133	VALIUM20
28730	CG10137	CG10137	VALIUM10
28304	CG10151	CG10151	VALIUM10
32959	CG10163	CG10163	VALIUM20
28377	CG10164	CG10164	VALIUM10
29536	CG1017	CG1017	VALIUM10
25945	CG10177	CG10177	VALIUM10
33047	CG10188	CG10188	VALIUM20
28759	CG10200	CG10200	VALIUM10
28640	CG10205	CG10205	VALIUM10
33432	CG10249	CG10249	VALIUM20
29360	CG10267	CG10267	VALIUM10
26239	CG10274	CG10274	VALIUM10
26764	CG10321	CG10321	VALIUM10
29517	CG10327	CG10327	VALIUM10
27076	CG10348	CG10348	VALIUM10
26765	CG10366	CG10366	VALIUM10
25846	CG10440	CG10440	VALIUM10
26002	CG10465	CG10465	VALIUM10
28379	CG10566	CG10566	VALIUM10
28001	CG10631	CG10631	VALIUM10
27998	CG10654	CG10654	VALIUM10
32958	CG10672	CG10672	VALIUM20

28580	CG10738	CG10738	VALIUM10
29599	CG10804	CG10804	VALIUM10
28557	CG10814	CG10814	VALIUM10
25848	CG10830	CG10830	VALIUM10
27239	CG10858	CG10858	VALIUM10
25878	CG10864	CG10864	VALIUM10
25806	CG1090	CG1090	VALIUM10
26231	CG10949	CG10949	VALIUM10
29361	CG10959	CG10959	VALIUM10
29362	CG10979	CG10979	VALIUM10
27267	CG10981	CG10981	VALIUM10
28602	CG10992	CG10992	VALIUM10
26766	CG11071	CG11071	VALIUM10
26020	CG11105	CG11105	VALIUM10
28637	CG11134	CG11134	VALIUM10
28893	CG11136	CG11136	VALIUM10
28896	CG11148	CG11148	VALIUM10
28638	CG11163	CG11163	VALIUM10
28629	CG11180	CG11180	VALIUM10
28301	CG11206	CG11206	VALIUM10
29603	CG11221	CG11221	VALIUM10
28615	CG1129	CG1129	VALIUM10
28641	CG11294	CG11294	VALIUM10
28065	CG11317	CG11317	VALIUM10
26003	CG11340	CG11340	VALIUM10
28005	CG11376	CG11376	VALIUM10
28309	CG11448	CG11448	VALIUM10
26240	CG11456	CG11456	VALIUM10
28312	CG11534	CG11534	VALIUM10
29609	CG11550	CG11550	VALIUM10
29531	CG11576	CG11576	VALIUM10
33337	CG11585	CG11585	VALIUM20
32924	CG11593	CG11593	VALIUM20
27999	CG11696	CG11696	VALIUM10
32933	CG11700	CG11700	VALIUM20
28567	CG11722	CG11722	VALIUM10
29349	CG11723	CG11723	VALIUM10
32505	CG11873	CG11873	VALIUM20
26021	CG11891	CG11891	VALIUM10
29363	CG11902	CG11902	VALIUM10
26767	CG11906	CG11906	VALIUM10
28624	CG11910	CG11910	VALIUM10
28933	CG11949	CG11949	VALIUM10
32507	CG11984	CG11984	VALIUM20
28943	CG11992	CG11992	VALIUM10
28382	CG12038	CG12038	VALIUM10
28592	CG12050	CG12050	VALIUM10
26248	CG12061	CG12061	VALIUM10

27677	CG12069	CG12069	VALIUM10
26768	CG12071	CG12071	VALIUM10
28968	CG12117	CG12117	VALIUM10
26232	CG12155	CG12155	VALIUM10
28000	CG12219	CG12219	VALIUM10
28727	CG12239	CG12239	VALIUM10
33729	CG12241	CG12241	VALIUM20
32436	CG12262	CG12262	VALIUM20
32437	CG12262	CG12262	VALIUM20
32877	CG12264	CG12264	VALIUM20
29412	CG12301	CG12301	VALIUM10
26250	CG12344	CG12344	VALIUM10
29610	CG12370	CG12370	VALIUM10
32919	CG12393	CG12393	VALIUM20
33428	CG12393	CG12393	VALIUM20
28754	CG12432	CG12432	VALIUM10
25882	CG12455	CG12455	VALIUM10
29611	CG12594	CG12594	VALIUM10
29364	CG12605	CG12605	VALIUM10
28318	CG12645	CG12645	VALIUM10
26769	CG12769	CG12769	VALIUM10
27678	CG12783	CG12783	VALIUM10
29612	CG12796	CG12796	VALIUM10
29418	CG12858	CG12858	VALIUM10
27077	CG12942	CG12942	VALIUM10
26284	CG1299	CG1299	VALIUM10
28632	CG13114	CG13114	VALIUM10
27240	CG13120	CG13120	VALIUM10
25883	CG13121	CG13121	VALIUM10
29545	CG13123	CG13123	VALIUM10
28755	CG13139	CG13139	VALIUM10
29419	CG13229	CG13229	VALIUM10
28761	CG13248	CG13248	VALIUM10
28642	CG13253	CG13253	VALIUM10
28643	CG13532	CG13532	VALIUM10
27241	CG13568	CG13568	VALIUM10
25827	CG13575	CG13575	VALIUM10
28644	CG13579	CG13579	VALIUM10
28597	CG13604	CG13604	VALIUM10
25983	CG13624	CG13624	VALIUM10
33341	CG13692	CG13692	VALIUM20
33342	CG13720	CG13720	VALIUM20
28057	CG13779	CG13779	VALIUM10
28058	CG13784	CG13784	VALIUM10
32917	CG13887	CG13887	VALIUM20
32918	CG13887	CG13887	VALIUM20
27243	CG13894	CG13894	VALIUM10
29613	CG13897	CG13897	VALIUM10

33731	CG13900	CG13900	VALIUM20
33702	CG13902	CG13902	VALIUM20
28645	CG13908	CG13908	VALIUM10
28646	CG13928	CG13928	VALIUM10
26733	CG13995	CG13995	VALIUM10
29456	CG14014	CG14014	VALIUM10
33732	CG14034	CG14034	VALIUM20
27073	CG14050	CG14050	VALIUM10
27555	CG14119	CG14119	VALIUM10
33343	CG14137	CG14137	VALIUM20
28762	CG1416	CG1416	VALIUM10
29420	CG14186	CG14186	VALIUM10
28012	CG14239	CG14239	VALIUM10
28763	CG14274	CG14274	VALIUM10
29614	CG14313	CG14313	VALIUM10
28528	CG14351	CG14351	VALIUM10
33344	CG14377	CG14377	VALIUM20
28647	CG14419	CG14419	VALIUM10
32487	CG14446	CG14446	VALIUM20
28530	CG14485	CG14485	VALIUM10
33345	CG14507	CG14507	VALIUM20
28764	CG14509	CG14509	VALIUM10
33045	CG14563	CG14563	VALIUM20
25855	CG14593	CG14593	VALIUM10
27032	CG14647	CG14647	VALIUM10
26770	CG14655	CG14655	VALIUM10
29365	CG14667	CG14667	VALIUM10
26771	CG14710	CG14710	VALIUM10
29615	CG14762	CG14762	VALIUM10
32384	CG14945	CG14945	VALIUM20
32905	CG14945	CG14945	VALIUM20
26772	CG14962	CG14962	VALIUM10
28765	CG15021	CG15021	VALIUM10
33713	CG15022	CG15022	VALIUM20
28517	CG1504	CG1504	VALIUM10
28366	CG15072	CG15072	VALIUM10
29366	CG15073	CG15073	VALIUM10
33693	CG15117	CG15117	VALIUM20
28649	CG15236	CG15236	VALIUM10
28650	CG15270	CG15270	VALIUM10
29530	CG15309	CG15309	VALIUM10
28631	CG15423	CG15423	VALIUM10
28766	CG15522	CG15522	VALIUM10
28767	CG15537	CG15537	VALIUM10
28016	CG15553	CG15553	VALIUM10
26006	CG15555	CG15555	VALIUM10
29616	CG15651	CG15651	VALIUM10
28976	CG15658	CG15658	VALIUM10

26773	CG15710	CG15710	VALIUM10
28516	CG15744	CG15744	VALIUM10
29421	CG15765	CG15765	VALIUM10
28910	CG15797	CG15797	VALIUM10
28356	CG15817	CG15817	VALIUM10
28768	CG15890	CG15890	VALIUM10
27063	CG1603	CG1603	VALIUM10
29350	CG1621	CG1621	VALIUM10
32508	CG1636	CG1636	VALIUM20
33733	CG1637	CG1637	VALIUM20
27078	CG1663	CG1663	VALIUM10
28956	CG16757	CG16757	VALIUM10
29617	CG16868	CG16868	VALIUM10
25809	CG1688	CG1688	VALIUM10
26774	CG16899	CG16899	VALIUM10
33051	CG17002	CG17002	VALIUM20
26007	CG17139	CG17139	VALIUM10
29358	CG17153	CG17153	VALIUM10
26775	CG17181	CG17181	VALIUM10
27079	CG17186	CG17186	VALIUM10
28769	CG17219	CG17219	VALIUM10
28895	CG17255	CG17255	VALIUM10
27028	CG17257	CG17257	VALIUM10
28553	CG17319	CG17319	VALIUM10
29422	CG1732	CG1732	VALIUM10
29618	CG17321	CG17321	VALIUM10
28898	CG17328	CG17328	VALIUM10
32390	CG17331	CG17331	VALIUM20
26776	CG17359	CG17359	VALIUM10
33734	CG17385	CG17385	VALIUM20
25956	CG17386	CG17386	VALIUM10
32509	CG17494	CG17494	VALIUM20
26292	CG17528	CG17528	VALIUM10
27033	CG1756	CG1756	VALIUM10
28344	CG17593	CG17593	VALIUM10
26008	CG17646	CG17646	VALIUM10
25924	CG17664	CG17664	VALIUM10
28581	CG17667	CG17667	VALIUM10
28541	CG1768	CG1768	VALIUM10
28770	CG17739	CG17739	VALIUM10
33347	CG17746	CG17746	VALIUM20
29367	CG17803	CG17803	VALIUM10
28023	CG17843	CG17843	VALIUM10
27996	CG17912	CG17912	VALIUM10
26709	CG1792	CG1792	VALIUM10
26009	CG17922	CG17922	VALIUM10
25810	CG18110	CG18110	VALIUM10
32927	CG18178	CG18178	VALIUM20

28521	CG18249	CG18249	VALIUM10
33348	CG18262	CG18262	VALIUM20
28560	CG18265	CG18265	VALIUM10
28520	CG18278	CG18278	VALIUM10
27080	CG1832	CG1832	VALIUM10
28942	CG1832	CG1832	VALIUM10
33735	CG18446	CG18446	VALIUM20
27081	CG1845	CG1845	VALIUM10
26710	CG18476	CG18476	VALIUM10
28529	CG18480	CG18480	VALIUM10
32957	CG18616	CG18616	VALIUM20
29542	CG18619	CG18619	VALIUM10
28024	CG1909	CG1909	VALIUM10
28573	CG1970	CG1970	VALIUM10
28771	CG2016	CG2016	VALIUM10
28579	CG2021	CG2021	VALIUM10
26711	CG2052	CG2052	VALIUM10
33692	CG2064	CG2064	VALIUM20
26712	CG2116	CG2116	VALIUM10
33750	CG2186	CG2186	VALIUM20
27082	CG2199	CG2199	VALIUM10
26241	CG2202	CG2202	VALIUM10
29619	CG2225	CG2225	VALIUM10
33736	CG2469	CG2469	VALIUM20
33649	CG2577	CG2577	VALIUM20
28753	CG2641	CG2641	VALIUM10
28630	CG2678	CG2678	VALIUM10
29322	CG2747	CG2747	VALIUM10
29516	CG2762	CG2762	VALIUM10
28965	CG2807	CG2807	VALIUM10
33650	CG2807	CG2807	VALIUM20
29333	CG2875	CG2875	VALIUM10
25851	CG2893	CG2893	VALIUM10
28319	CG2962	CG2962	VALIUM10
33596	CG2982	CG2982	VALIUM20
29368	CG30020	CG30020	VALIUM10
27281	CG30044	CG30044	VALIUM10
28651	CG30046	CG30046	VALIUM10
28607	CG30059	CG30059	VALIUM10
28555	CG30060	CG30060	VALIUM10
28321	CG30089	CG30089	VALIUM10
27669	CG30106	CG30106	VALIUM10
29423	CG30172	CG30172	VALIUM10
28772	CG30203	CG30203	VALIUM10
28652	CG30340	CG30340	VALIUM10
32493	CG30356	CG30356	VALIUM20
32494	CG30356	CG30356	VALIUM20
28988	CG30379	CG30379	VALIUM10

27557	CG30382	CG30382	VALIUM10
27687	CG30414	CG30414	VALIUM10
28653	CG31044	CG31044	VALIUM10
29581	CG3105	CG3105	VALIUM10
28891	CG31057	CG31057	VALIUM10
27087	CG31065	CG31065	VALIUM10
28359	CG31103	CG31103	VALIUM10
28706	CG31105	CG31105	VALIUM10
29582	CG31106	CG31106	VALIUM10
32495	CG31156	CG31156	VALIUM20
32982	CG31156	CG31156	VALIUM20
28604	CG31183	CG31183	VALIUM10
28773	CG31235	CG31235	VALIUM10
28030	CG31272	CG31272	VALIUM10
29312	CG31302	CG31302	VALIUM10
28774	CG31324	CG31324	VALIUM10
26312	CG31460	CG31460	VALIUM10
27550	CG31469	CG31469	VALIUM10
29404	CG3162	CG3162	VALIUM10
28654	CG31646	CG31646	VALIUM10
29457	CG31665	CG31665	VALIUM10
26778	CG31670	CG31670	VALIUM10
29301	CG3168	CG3168	VALIUM10
28775	CG31716	CG31716	VALIUM10
28017	CG31720	CG31720	VALIUM10
29424	CG31809	CG31809	VALIUM10
27286	CG32062	CG32062	VALIUM10
32970	CG32109	CG32109	VALIUM20
32447	CG32138	CG32138	VALIUM20
28692	CG32164	CG32164	VALIUM10
28308	CG32204	CG32204	VALIUM10
32974	CG32251	CG32251	VALIUM20
32875	CG3226	CG3226	VALIUM20
32988	CG3226	CG3226	VALIUM20
28340	CG32264	CG32264	VALIUM10
29527	CG32372	CG32372	VALIUM10
29621	CG32432	CG32432	VALIUM10
28776	CG32506	CG32506	VALIUM10
33351	CG3253	CG3253	VALIUM20
26750	CG32532	CG32532	VALIUM10
28621	CG32547	CG32547	VALIUM10
28611	CG3257	CG3257	VALIUM10
28074	CG12047	CG32599	VALIUM10
25962	CG32626	CG32626	VALIUM10
29449	CG32666	CG32666	VALIUM10
28702	CG32702	CG32702	VALIUM10
25888	CG32770	CG32770	VALIUM10
26715	CG32778	CG32778	VALIUM10

25814	CG32792	CG32792	VALIUM10
28822	CG32809	CG32809	VALIUM10
25889	CG32810	CG32810	VALIUM10
25925	CG32843	CG32843	VALIUM10
27296	CG3294	CG3294	VALIUM10
33352	CG33095	CG33095	VALIUM20
33751	CG33129	CG33129	VALIUM20
26306	CG33159	CG33159	VALIUM10
28299	CG33171	CG33171	VALIUM10
29458	CG33213	CG33213	VALIUM10
28914	CG33231	CG33231	VALIUM10
25816	CG33289	CG33289	VALIUM10
28540	CG33470	CG33470	VALIUM10
25838	CG33523	CG33523	VALIUM10
28609	CG3353	CG3353	VALIUM10
28614	CG33639	CG33639	VALIUM10
28051	CG33673	CG33673	VALIUM10
28380	CG33696	CG33696	VALIUM10
28296	CG3371	CG3371	VALIUM10
28932	CG33960	CG33960	VALIUM10
28683	CG12600	CG33967	VALIUM10
25818	CG3397	CG3397	VALIUM10
28907	CG3413	CG3413	VALIUM10
32387	CG34207	CG34207	VALIUM20
29324	CG42611, CG34352	CG34352	VALIUM10
27301	CG34354	CG34354	VALIUM10
33674	CG34354	CG34354	VALIUM20
28524	CG9783	CG34357	VALIUM10
29459	CG34361	CG34361	VALIUM10
25819	CG34366	CG34366	VALIUM10
25892	CG34369	CG34369	VALIUM10
28729	CG34370	CG34370	VALIUM10
29371	CG34376	CG34376	VALIUM10
26011	CG34396	CG34396	VALIUM10
26016	CG34402	CG34402	VALIUM10
28655	CG34411	CG34411	VALIUM10
27056	CG3494	CG3494	VALIUM10
32898	CG3508	CG3508	VALIUM20
28930	CG3523	CG3523	VALIUM10
25864	CG3530	CG3530	VALIUM10
33711	CG3542	CG3542	VALIUM20
33651	CG3605	CG3605	VALIUM20
28303	CG3618	CG3618	VALIUM10
26184	CG3654	CG3654	VALIUM10
32883	CG3689	CG3689	VALIUM20
27688	CG3690	CG3690	VALIUM10
29596	CG3703	CG3703	VALIUM10
33422	CG3711	CG3711	VALIUM20

29451	CG3814	CG3814	VALIUM10
33354	CG3814	CG3814	VALIUM20
25852	CG3822	CG3822	VALIUM10
25991	CG3891	CG3891	VALIUM10
33355	CG3919	CG3919	VALIUM20
28777	CG3967	CG3967	VALIUM10
27997	CG3995	CG3995	VALIUM10
27245	CG17167	CG40146	VALIUM10
32973	CG4078	CG4078	VALIUM20
28370	CG4133	CG4133	VALIUM10
28736	CG4168	CG4168	VALIUM10
25877	CG42237	CG42237	VALIUM10
26723	CG42260	CG42260	VALIUM10
29411	CG42271, CG33249	CG42271	VALIUM10
28558	CG42321	CG42321	VALIUM10
33356	CG42327	CG42327	VALIUM20
27293	CG42333	CG42333	VALIUM10
27257	CG42340	CG42340	VALIUM10
28648	CG14889	CG42342	VALIUM10
28958	CG42346	CG42346	VALIUM10
26735	CG42347	CG42347	VALIUM10
28937	CG42355	CG42355	VALIUM10
32440	CG42358	CG42358	VALIUM20
32980	CG42358	CG42358	VALIUM20
27294	CG42366	CG42366	VALIUM10
33677	CG42517,CG42518	CG42518	VALIUM20
29408	CG42575, CG7628	CG42575	VALIUM10
29593	CG42594, CG32771	CG42594, CG32771	VALIUM10
33665	CG42666	CG42666	VALIUM20
32332	CG4267	CG4267	VALIUM20
33737	CG42724	CG42724	VALIUM20
26313	CG4282	CG4282	VALIUM10
29305	CG4288	CG4288	VALIUM10
33357	CG4294	CG4294	VALIUM20
32960	CG4313	CG4313	VALIUM20
27987	CG4328	CG4328	VALIUM10
33349	CG43340	CG43340	VALIUM20
33006	CG43355,CG4922	CG43355 | spalt-adjacent	VALIUM20
32341	CG43658	CG43658	VALIUM20
29623	CG4395	CG4395	VALIUM10
33350	CG44153	CG44153	VALIUM20
28536	CG4547	CG4547	VALIUM10
32842	CG4562	CG4562	VALIUM20
32893	CG4565	CG4565	VALIUM20
25893	CG4587	CG4587	VALIUM10
28617	CG4596	CG4596	VALIUM10

25993	CG4617	CG4617	VALIUM10
28531	CG4618	CG4618	VALIUM10
27310	CG4629	CG4629	VALIUM10
27045	CG4655	CG4655	VALIUM10
29307	CG4670	CG4670	VALIUM10
25873	CG4681	CG4681	VALIUM10
33696	CG4747	CG4747	VALIUM20
25894	CG4822	CG4822	VALIUM10
27046	CG4839	CG4839	VALIUM10
28738	CG4872	CG4872	VALIUM10
32392	CG5001	CG5001	VALIUM20
33359	CG5022	CG5022	VALIUM20
28305	CG5027	CG5027	VALIUM10
28323	CG5053	CG5053	VALIUM10
27313	CG5098	CG5098	VALIUM10
32393	CG5144	CG5144	VALIUM20
27660	CG5160	CG5160	VALIUM10
32881	CG5174	CG5174	VALIUM20
32987	CG5174	CG5174	VALIUM20
32888	CG5181	CG5181	VALIUM20
28069	CG5226	CG5226	VALIUM10
27694	CG5235	CG5235	VALIUM10
27034	CG5284	CG5284	VALIUM10
32499	CG5292	CG5292	VALIUM20
32500	CG5292	CG5292	VALIUM20
32333	CG5337	CG5337	VALIUM20
28909	CG5370	CG5370	VALIUM10
32928	CG5514	CG5514	VALIUM20
28962	CG5567	CG5567	VALIUM10
32334	CG5589	CG5589	VALIUM20
25994	CG5591	CG5591	VALIUM10
32876	CG5599	CG5599	VALIUM20
27318	CG5618	CG5618	VALIUM10
25822	CG5621	CG5621	VALIUM10
28778	CG5629	CG5629	VALIUM10
28619	CG5644	CG5644	VALIUM10
33360	CG5646	CG5646	VALIUM20
28564	CG5656	CG5656	VALIUM10
32335	CG5726	CG5726	VALIUM20
28315	CG5733	CG5733	VALIUM10
32394	CG5745	CG5745	VALIUM20
27668	CG5853	CG5853	VALIUM10
27321	CG5890	CG5890	VALIUM10
28625	CG6024	CG6024	VALIUM10
28610	CG6044	CG6044	VALIUM10
28538	CG6134	CG6134	VALIUM10
27323	CG6151	CG6151	VALIUM10
28917	CG6178	CG6178	VALIUM10

28386	CG6218	CG6218	VALIUM10
33652	CG6272	CG6272	VALIUM20
29331	CG6272	CG6272	VALIUM10
27064	CG6276	CG6276	VALIUM10
28297	CG6329	CG6329	VALIUM10
28745	CG6356	CG6356	VALIUM10
32441	CG6418	CG6418	VALIUM20
32442	CG6418	CG6418	VALIUM20
29452	CG6495	CG6495	VALIUM10
27698	CG6498	CG6498	VALIUM10
27488	CG6560	CG6560	VALIUM10
26233	CG6683	CG6683	VALIUM10
28364	CG6690	CG6690	VALIUM10
28751	CG6697	CG6697	VALIUM10
27560	CG6701	CG6701	VALIUM10
28316	CG6707	CG6707	VALIUM10
29425	CG6723	CG6723	VALIUM10
25803	CG6737	CG6737	VALIUM10
27561	CG6770	CG6770	VALIUM10
32395	CG6791	CG6791	VALIUM20
29514	CG6794	CG6794	VALIUM10
32380	CG6805	CG6805	VALIUM20
29369	CG6813	CG6813	VALIUM10
28950	CG6831	CG6831	VALIUM10
33423	CG6903	CG6903	VALIUM20
27716	CG6907	CG6907	VALIUM10
32336	CG6914	CG6914	VALIUM20
32448	CG6923	CG6923	VALIUM20
25895	CG6927	CG6927	VALIUM10
27501	CG7039	CG7039	VALIUM10
28656	CG7045	CG7045	VALIUM10
28657	CG7046	CG7046	VALIUM10
32449	CG7065	CG7065	VALIUM20
28533	CG7158	CG7158	VALIUM10
29450	CG7197	CG7197	VALIUM10
32485	CG7200	CG7200	VALIUM20
27505	CG7236	CG7236	VALIUM10
28658	CG7299	CG7299	VALIUM10
32929	CG7324	CG7324	VALIUM20
32450	CG7362	CG7362	VALIUM20
28063	CG7369	CG7369	VALIUM10
32510	CG7420	CG7420	VALIUM20
25857	CG7431	CG7431	VALIUM10
28634	CG7456	CG7456	VALIUM10
32511	CG7556	CG7556	VALIUM20
27090	CG7589	CG7589	VALIUM10
32337	CG7627	CG7627	VALIUM20
32338	CG7692	CG7692	VALIUM20

28613	CG7708	CG7708	VALIUM10
26234	CG7745	CG7745	VALIUM10
27717	CG7757	CG7757	VALIUM10
32843	CG7787	CG7787	VALIUM20
28922	CG7800	CG7800	VALIUM10
25992	CG7839	CG7839	VALIUM10
32379	CG7840	CG7840	VALIUM20
28752	CG7841	CG7841	VALIUM10
28565	CG7896	CG7896	VALIUM10
28779	CG7945	CG7945	VALIUM10
25874	CG8007	CG8007	VALIUM10
32339	CG8021	CG8021	VALIUM20
32340	CG8034	CG8034	VALIUM20
32884	CG8036	CG8036	VALIUM20
25971	CG8092	CG8092	VALIUM10
27562	CG8108	CG8108	VALIUM10
32930	CG8108	CG8108	VALIUM20
33012	CG8128	CG8128	VALIUM20
27069	CG8216	CG8216	VALIUM10
29426	CG8245	CG8245	VALIUM10
33361	CG8289	CG8289	VALIUM20
27065	CG8290	CG8290	VALIUM10
27066	CG8359	CG8359	VALIUM10
32931	CG8368	CG8368	VALIUM20
25825	CG8546	CG8546	VALIUM10
25826	CG8594	CG8594	VALIUM10
28576	CG8680	CG8680	VALIUM10
25853	CG8713	CG8713	VALIUM10
29447	CG8765	CG8765	VALIUM10
32343	CG8765	CG8765	VALIUM20
29624	CG8784	CG8784	VALIUM10
28781	CG8795	CG8795	VALIUM10
29515	CG8852	CG8852	VALIUM10
29427	CG8861	CG8861	VALIUM10
28636	CG8907	CG8907	VALIUM10
32908	CG8915	CG8915	VALIUM20
25854	CG8916	CG8916	VALIUM10
29348	CG8924	CG8924	VALIUM10
33362	CG9005	CG9005	VALIUM20
29357	CG9139	CG9139	VALIUM10
29534	CG9140	CG9140	VALIUM10
25921	CG9194	CG9194	VALIUM10
28522	CG9311	CG9311	VALIUM10
29532	CG9344	CG9344	VALIUM10
28292	CG9368	CG9368	VALIUM10
33364	CG9386	CG9386	VALIUM20
28320	CG9411	CG9411	VALIUM10
26216	CG9418	CG9418	VALIUM10

26754	CG9437	CG9437	VALIUM10
28737	CG9454	CG9454	VALIUM10
26724	CG9467	CG9467	VALIUM10
32344	CG9548	CG9548	VALIUM20
28527	CG9588	CG9588	VALIUM10
28070	CG9636	CG9636	VALIUM10
26713	CG9650	CG9650	VALIUM10
28384	CG9657	CG9657	VALIUM10
28052	CG9722	CG9722	VALIUM10
26762	CG9727	CG9727	VALIUM10
32396	CG9775	CG9775	VALIUM20
32858	CG9797	CG9797	VALIUM20
29606	CG9911	CG9911	VALIUM10
27539	CG9918	CG9918	VALIUM10
27540	CG9934	CG9934	VALIUM10
28506	CG9935	CG9935	VALIUM10
28523	CG9977	CG9977	VALIUM10
27686	CG3324	cGMP-dependent protein kinase 21D	VALIUM10
27027	CG5229	chameau	VALIUM10
32484	CG5229	chameau	VALIUM20
26779	CG11798	charlatan	VALIUM10
33420	CG9594	Chd3	VALIUM20
28348	CG33320	CheB38a	VALIUM10
28014	CG33321	CheB38b	VALIUM10
27255	CG33351	CheB42b	VALIUM10
27089	CG33350	CheB42c	VALIUM10
26307	CG3937	cheerio	VALIUM10
28329	CG5686	chico	VALIUM10
33365	CG5813	chiffon	VALIUM20
28906	CG4108	Chmp1	VALIUM10
25856	CG12345	Choline acetyltransferase	VALIUM10
26777	CG31666	Chronologically inappropriate morphogenesis	VALIUM10
33638	CG31666	Chronologically inappropriate morphogenesis	VALIUM20
28033	CG3870	chrowded	VALIUM10
28003	CG4944	ciboulot	VALIUM10
28027	CG2945	cin	VALIUM10
28949	CG2945	cinnamon	VALIUM10
32425	CG2945,CG42376	cinnamon | CG42376	VALIUM20
33435	CG42574	circadian trip	VALIUM20
27524	CG8639	Cirl	VALIUM10
28927	CG7392	Cka	VALIUM10
27530	CG9012	Clathrin heavy chain	VALIUM10
27496	CG6948	Clathrin light chain	VALIUM10
26318	CG17100	clockwork orange	VALIUM10
27736	CG17100	clockwork orange	VALIUM10
28351	CG8681	clumsy	VALIUM10
28684	CG9176	CNG channel-like	VALIUM10
33741	CG6223	Coat Protein (coatomer) β	VALIUM20

27054	CG9613	Coenzyme Q biosynthesis protein 2	VALIUM10
33368	CG8710	coilin	VALIUM20
28381	CG17943	commissureless	VALIUM10
28967	CG7503	Con	VALIUM10
28923	CG1084	Cont	VALIUM10
27303	CG3889	COP9 complex homolog subunit 1 b	VALIUM10
28732	CG14884	COP9 complex homolog subunit 5	VALIUM10
33369	CG18332	COP9 signalosome subunit 3	VALIUM20
33663	CG2038	COP9 signalosome subunit 7	VALIUM20
33370	CG42522	COP9 signalosome subunit 8	VALIUM20
25999	CG3302	Corazonin	VALIUM10
33619	CG3954	corkscrew	VALIUM20
32871	CG3637	Cortactin	VALIUM20
28360	CG31243	couch potato	VALIUM10
26317	CG7760	cousin of atonal	VALIUM10
25875	CG10460	crammer	VALIUM10
29535	CG3193	crn	VALIUM10
27071	CG5069	crocodile	VALIUM10
27697	CG6383	crumbs	VALIUM10
25985	CG8669	cryptocephal	VALIUM10
25859	CG3772	cryptochrome	VALIUM10
32889	CG7583	C-terminal Binding Protein	VALIUM20
32968	CG13350	Ctf4	VALIUM20
28984	CG2125	cubitus interruptus	VALIUM10
26732	CG11181	cup	VALIUM10
29400	CG8727	cycle	VALIUM10
27648	CG7450	Cyclic-AMP response element binding protein A	VALIUM10
29332	CG6103	Cyclic-AMP response element binding protein B at 17A	VALIUM10
26014	CG7779	Cyclic-nucleotide-gated ion channel protein	VALIUM10
29313	CG5940	Cyclin A	VALIUM10
27718	CG9096	Cyclin D	VALIUM10
33653	CG9096	Cyclin D	VALIUM20
29314	CG3938	Cyclin E	VALIUM10
33654	CG3938	Cyclin E	VALIUM20
29315	CG11525	Cyclin G	VALIUM10
32976	CG6292	Cyclin T	VALIUM20
27714	CG5072	Cyclin-dependent kinase 4	VALIUM10
27517	CG8203	Cyclin-dependent kinase 5	VALIUM10
33001	CG9916	Cyclophilin 1	VALIUM20
28077	CG11715	Cyp4g15	VALIUM10
32932	CG6692	Cysteine proteinase-1	VALIUM20
33645	CG32206	Cysteine string protein	VALIUM20
27548	CG14724	Cytochrome c oxidase subunit Va	VALIUM10
28616	CG9745	D1 chromosomal protein	VALIUM10
33655	CG9745	D1 chromosomal protein	VALIUM20
33371	CG10269	D19A	VALIUM20
28623	CG2682	d4	VALIUM10

27664	CG10595	dachs	VALIUM10
26758	CG4952	dachshund	VALIUM10
28008	CG17941	dachsous	VALIUM10
32964	CG17941	dachsous	VALIUM20
26218	CG7055	dalao	VALIUM10
26319	CG5102	daughterless	VALIUM10
29326	CG5102	daughterless	VALIUM10
26235	CG5201	Daughters against dpp	VALIUM10
28598	CG4792	Dcr-1	VALIUM10
27531	CG9054	Dead-box-1	VALIUM10
32375	CG9252	deadlock	VALIUM20
26320	CG8704	deadpan	VALIUM10
27083	CG33134	death executioner Bcl-2 homologue	VALIUM10
25782	CG9885	decapentaplegic	VALIUM10
33618	CG9885	decapentaplegic	VALIUM20
33628	CG9885	decapentaplegic	VALIUM20
29524	CG1385	Def	VALIUM10
28743	CG12390	defective in the avoidance of repellents	VALIUM10
26225	CG5799	defective proventriculus	VALIUM10
25812	CG31623	defective transmitter release	VALIUM10
27720	CG18176	deflated	VALIUM10
26751	CG2189	Deformed	VALIUM10
32512	CG8567	Deformed epidermal autoregulatory factor-1	VALIUM20
28696	CG5935	Dek	VALIUM10
25973	CG5441	delilah	VALIUM10
28032	CG3619	Delta	VALIUM10
27041	CG3929	deltex	VALIUM10
29602	CG17348	derailed	VALIUM10
25961	CG3915	Derailed 2	VALIUM10
26734	CG16792	Developmental embryonic B	VALIUM10
33424	CG1768	diaphanous	VALIUM20
27486	CG6493	Dicer-2	VALIUM10
33656	CG6493	Dicer-2	VALIUM20
26217	CG5893	Dichaete	VALIUM10
28905	CG7098	dik	VALIUM10
32397	CG42799	dikar	VALIUM20
28620	CG2146	dilute class unconventional myosin	VALIUM10
25783	CG10798	diminutive	VALIUM10
25784	CG10798	diminutive	VALIUM10
28659	CG9908	disconnected	VALIUM10
25780	CG1725	discs large 1	VALIUM10
33620	CG1725	discs large 1	VALIUM20
27288	CG32315	discs lost	VALIUM10
27719	CG2048	discs overgrown	VALIUM10
32451	CG7098	diskette	VALIUM20
27247	CG2019	dispatched	VALIUM10
29373	CG9019	dissatisfaction	VALIUM10
28378	CG13651	distal antenna-related	VALIUM10

29337	CG3629	Distal-less	VALIUM10
25804	CG8348	Diuretic hormone	VALIUM10
28780	CG8422	Diuretic hormone 44 receptor 1	VALIUM10
28747	CG4974	division abnormally delayed	VALIUM10
28596	CG8269	Dmn	VALIUM10
28628	CG8364	DNA fragmentation factor-related protein 3	VALIUM10
32899	CG10578	DnaJ-like-1	VALIUM20
32978	CG10578	DnaJ-like-1	VALIUM20
33410	CG7143	DNApol-η	VALIUM20
32872	CG5170	Dodeca-satellite-binding protein 1	VALIUM20
28983	CG14226	domeless	VALIUM10
32860	CG14226	domeless	VALIUM20
27030	CG10697	Dopa decarboxylase	VALIUM10
26001	CG33517	Dopamine 2-like receptor	VALIUM10
26243	CG3318	Dopamine N acetyltransferase	VALIUM10
26018	CG18741	Dopamine receptor 2	VALIUM10
27650	CG6667	dorsal	VALIUM10
32934	CG6667	dorsal	VALIUM20
27265	CG9771	Dorsal interacting protein 2	VALIUM10
27067	CG12767	Dorsal interacting protein 3	VALIUM10
26716	CG11094	doublesex	VALIUM10
27657	CG5737	doublesex-Mab related 93B	VALIUM10
29628	CG17800	Down syndrome cell adhesion molecule	VALIUM10
28685	CG15793	Downstream of raf1	VALIUM10
32920	CG15793	Downstream of raf1	VALIUM20
33639	CG15793	Downstream of raf1	VALIUM20
27563	CG6033	downstream of receptor kinase	VALIUM10
33372	CG4654	DP transcription factor	VALIUM20
27991	CG32057	dpr10	VALIUM10
28782	CG34385	dpr12	VALIUM10
29626	CG10946	dpr14	VALIUM10
29604	CG14948	dpr18	VALIUM10
28293	CG12191	dpr20	VALIUM10
29627	CG5308	dpr5	VALIUM10
28744	CG32600	dpr8	VALIUM10
33409	CG33485	dpr9	VALIUM20
28975	CG10794	DptB	VALIUM10
27728	CG3727	dreadlocks	VALIUM10
26245	CG6440	Dromyosuppressin	VALIUM10
27529	CG8985	Dromyosuppressin receptor 1	VALIUM10
25832	CG13803	Dromyosuppressin receptor 2	VALIUM10
26224	CG1897	Drop	VALIUM10
27704	CG8730	drosha	VALIUM10
33657	CG8730	drosha	VALIUM20
33549	CG32627	Drosophila Nna1 ortholog	VALIUM20
25869	CG18090	Drosulfakinin	VALIUM10
32903	CG3131	Dual oxidase	VALIUM20
27250	CG32498	dunce	VALIUM10

25879	CG1099	Dynamin associated protein 160	VALIUM10
27682	CG3210	Dynamin related protein 1	VALIUM10
28749	CG7507	Dynein heavy chain 64C	VALIUM10
26321	CG32474	dysfusion	VALIUM10
32935	CG8529	Dystrobrevin-like	VALIUM20
25977	CG8346	E(spl) region transcript m3	VALIUM10
29378	CG6099	E(spl) region transcript m4	VALIUM10
26201	CG6096	E(spl) region transcript m5	VALIUM10
29327	CG8361	E(spl) region transcript m7	VALIUM10
26202	CG14548	E(spl) region transcript mβ	VALIUM10
25978	CG8333	E(spl) region transcript mγ	VALIUM10
26203	CG8328	E(spl) region transcript mδ	VALIUM10
27564	CG6376	E2F transcription factor	VALIUM10
27995	CG1071	E2F transcription factor 2	VALIUM10
29629	CG7383	eagle	VALIUM10
25821	CG5076	eag-like K+ channel	VALIUM10
26252	CG3327	Early gene at 23	VALIUM10
25863	CG6124	eater	VALIUM10
28605	CG3265	Eb1	VALIUM10
28612	CG3331	ebony	VALIUM10
26242	CG18105	Ecdysis triggering hormone	VALIUM10
29374	CG1765	Ecdysone receptor	VALIUM10
28901	CG10579	Ecdysone-induced protein 63E	VALIUM10
29353	CG32180	Ecdysone-induced protein 74EF	VALIUM10
29525	CG8127	Ecdysone-induced protein 75B	VALIUM10
33640	CG10160	Ecdysone-inducible gene L3	VALIUM20
26244	CG5400	Eclosion hormone	VALIUM10
28584	CG6311	Edc3	VALIUM10
29605	CG4912	eEF1delta	VALIUM10
28687	CG9659	egghead	VALIUM10
32445	CG12196	eggless	VALIUM20
28969	CG4051	egl	VALIUM10
27565	CG9805	eIF3-S10	VALIUM10
32880	CG4878	eIF3-S9	VALIUM20
32444	CG7483	eIF4AIII	VALIUM20
32907	CG7483	eIF4AIII	VALIUM20
26717	CG8127	Eip75B	VALIUM10
26718	CG18023	Eip78C	VALIUM10
32868	CG6050	Elongation factor Tu mitochondrial	VALIUM20
28371	CG4262	embryonic lethal abnormal vision	VALIUM10
28726	CG2988	empty spiracles	VALIUM10
27679	CG14296	endophilin A	VALIUM10
27537	CG9834	endophilin B	VALIUM10
26752	CG9015	engrailed	VALIUM10
33715	CG9015	engrailed	VALIUM20
33658	CG32346	Enhancer of bithorax	VALIUM20
28686	CG7776	Enhancer of Polycomb	VALIUM10
26322	CG8365	Enhancer of split	VALIUM10

32345	CG6474	enhancer of yellow 1	VALIUM20
32346	CG12238	enhancer of yellow 3	VALIUM20
27993	CG6502	Enhancer of zeste	VALIUM10
33659	CG6502	Enhancer of zeste	VALIUM20
28068	CG4913	ENL/AF9-related	VALIUM10
29518	CG11290	enok	VALIUM10
26300	CG17654	Enolase	VALIUM10
29317	CG34392	Epac	VALIUM10
28511	CG1511	Eph receptor tyrosine kinase	VALIUM10
27039	CG1862	Ephrin	VALIUM10
25781	CG10079	Epidermal growth factor receptor	VALIUM10
29578	CG16932	Epidermal growth factor receptor pathway substrate clone 15	VALIUM10
28890	CG9543	epsilonCOP	VALIUM10
28987	CG42250	epsin-like	VALIUM10
28514	CG3758	escargot	VALIUM10
27085	CG7404	estrogen-related receptor	VALIUM10
28783	CG5911	ETHR	VALIUM10
25795	CG6338	Ets at 97D	VALIUM10
28700	CG5583	Ets at 98B	VALIUM10
29316	CG8053	Eukaryotic initiation factor 1A	VALIUM10
33049	CG10811	eukaryotic translation initiation factor 4G	VALIUM20
28734	CG2328	even skipped	VALIUM10
28041	CG7127	exo70	VALIUM10
28712	CG6095	exo84	VALIUM10
28703	CG4114	expanded	VALIUM10
32347	CG3923	Exportin 6	VALIUM20
26738	CG1007	extra macrochaetae	VALIUM10
29338	CG8933	extradenticle	VALIUM10
28902	CG8994	exu	VALIUM10
26226	CG10488	eyegone	VALIUM10
29339	CG1464	eyeless	VALIUM10
32486	CG1464	eyeless	VALIUM20
28733	CG9554	eyes absent	VALIUM10
33687	CG4609	failed axon connections	VALIUM20
28694	CG3969	Fak-like tyrosine kinase	VALIUM10
33374	CG9351	falafel	VALIUM20
33050	CG12812	Fancl	VALIUM20
32972	CG13745	Fanconi anemia complementation group I homologue	VALIUM20
28044	CG7919	farinelli	VALIUM10
28990	CG3665	Fasciclin 2	VALIUM10
29566	CG3352	fat	VALIUM10
32925	CG12252	Fcp1	VALIUM20
33008	CG3218	female sterile (1) K10	VALIUM20
33630	CG3218	female sterile (1) K10	VALIUM20
27567	CG2637	Female sterile (2) Ketel	VALIUM10
32452	CG10528	female sterile (2) ltoPP43	VALIUM20
28926	CG11207	feo	VALIUM10

33375	CG8874	FER ortholog (H. sapiens)	VALIUM20
25966	CG14991	Fermitin 1	VALIUM10
28568	CG34368	Fili	VALIUM10
32936	CG8649	Fimbrin	VALIUM20
32348	CG6226	FK506-binding protein 1	VALIUM20
28349	CG4535	FK506-binding protein FKBP59	VALIUM10
27566	CG1484	flightless I	VALIUM10
27484	CG6203	Fmr1	VALIUM10
25858	CG2114	Fmrf Receptor	VALIUM10
25870	CG2346	FMRFamide-related	VALIUM10
29323	CG10023	Focal Adhesion Kinase	VALIUM10
33617	CG10023	Focal adhesion kinase	VALIUM20
27072	CG10002	fork head	VALIUM10
25997	CG3143	forkhead box, sub-group O	VALIUM10
32427	CG3143	forkhead box, sub-group O	VALIUM20
32993	CG3143	forkhead box, sub-group O	VALIUM20
26761	CG11922	forkhead domain 96Cb	VALIUM10
32398	CG33556	formin 3	VALIUM20
28784	CG4396	found in neurons	VALIUM10
29396	CG7004	four wheel drive	VALIUM10
28009	CG10917	four-jointed	VALIUM10
27696	CG5744	Frequenin 1	VALIUM10
28711	CG5907	Frequenin 2	VALIUM10
25947	CG10580	fringe	VALIUM10
27568	CG9739	frizzled 2	VALIUM10
33376	CG9434	Frost	VALIUM20
27509	CG7665	Fsh-Tsh-like receptor	VALIUM10
27659	CG4059	ftz transcription factor 1	VALIUM10
33625	CG4059	ftz transcription factor 1	VALIUM20
28554	CG9233	fu2	VALIUM10
25837	CG10772	Furin 1	VALIUM10
25959	CG18734	Furin 2	VALIUM10
28307	CG1500	furrowed	VALIUM10
28298	CG8824	fused lobes	VALIUM10
28507	CG8770	G protein beta-subunit 76C	VALIUM10
28010	CG2204	G protein oalpha 47A	VALIUM10
29576	CG2835	G protein salpha 60A	VALIUM10
25930	CG12232	G protein α 73B	VALIUM10
25934	CG8261	G protein γ 1	VALIUM10
25932	CG3694	G protein γ30A	VALIUM10
28354	CG40129	G protein-coupled receptor kinase 1	VALIUM10
29541	CG2995	G9a	VALIUM10
33011	CG3825	Gadd34	VALIUM20
28889	CG1528	gammaCop	VALIUM10
28707	CG3988	gamma-soluble NSF attachment protein	VALIUM10
32513	CG17566	gamma-Tubulin at 37C	VALIUM20
33548	CG3917	Gamma-tubulin ring protein 84	VALIUM20
33747	CG5034	GATAd	VALIUM20

33748	CG10278	GATAe	VALIUM20
28310	CG10763	Gbeta5	VALIUM10
28913	CG12245	gcm	VALIUM10
28904	CG3858	gcm2	VALIUM10
27309	CG4422	GDP dissociation inhibitor	VALIUM10
32349	CG6181	Ge-1	VALIUM20
28928	CG9491	Gef26	VALIUM10
33408	CG6539	Gemin3	VALIUM20
26214	CG30011	gemini	VALIUM10
26323	CG6211	germ cell-expressed bHLH-PAS	VALIUM10
32492	CG8411	germ cell-less	VALIUM20
28899	CG11861	gft	VALIUM10
26742	CG7952	giant	VALIUM10
27251	CG32538	giant fibre A	VALIUM10
28066	CG6963	gilgamesh	VALIUM10
26780	CG7672	glass	VALIUM10
33668	CG6946	glorund	VALIUM20
27721	CG9206	Glued	VALIUM10
32350	CG11397	gluon	VALIUM20
28334	CG14447	Glutamate receptor binding protein	VALIUM10
27521	CG8442	Glutamate receptor I	VALIUM10
27673	CG4481	Glutamate receptor IB	VALIUM10
27497	CG6992	Glutamate receptor IIA	VALIUM10
28718	CG7234	Glutamate receptor IIB	VALIUM10
25836	CG4226	Glutamate receptor IIC	VALIUM10
25942	CG31201	Glutamate receptor IIE	VALIUM10
28079	CG14994	Glutamic acid decarboxylase 1	VALIUM10
26302	CG8893	Glyceraldehyde 3 phosphate dehydrogenase 2	VALIUM10
29589	CG7446	Glycine receptor	VALIUM10
33634	CG7254	Glycogen phosphorylase	VALIUM20
28785	CG2679	goliath	VALIUM10
29577	CG11325	Gonadotropin-releasing hormone receptor	VALIUM10
29600	CG3388	gooseberry	VALIUM10
28078	CG2692	gooseberry-neuro	VALIUM10
32400	CG5820	Gp150	VALIUM20
27658	CG9656	grain	VALIUM10
33746	CG9656	grain	VALIUM20
28820	CG42311	grainy head	VALIUM10
27277	CG17161	grapes	VALIUM10
26017	CG10698	GRHRII	VALIUM10
32961	CG6964	Grunge	VALIUM20
32399	CG33546	GST-containing FLYWCH zinc-finger protein	VALIUM20
28885	CG8938	GstS1	VALIUM10
28748	CG1912	Guanylyl cyclase alpha-subunit at 99B	VALIUM10
28660	CG8742	Guanylyl cyclase at 76C	VALIUM10
28786	CG1470	Guanylyl cyclase beta-subunit at 100B	VALIUM10
25815	CG33151	Gustatory receptor 59e	VALIUM10
28608	CG4154	Gyc88E	VALIUM10

29529	CG14886	Gyc-89Db	VALIUM10
27315	CG5460	Hairless	VALIUM10
27738	CG6494	hairy	VALIUM10
26728	CG31753	hamlet	VALIUM10
32470	CG31753	hamlet	VALIUM20
28977	CG18144	Hand	VALIUM10
28549	CG1770	HDAC4	VALIUM10
27070	CG5748	Heat shock factor	VALIUM10
33007	CG4466	Heat shock protein 27	VALIUM20
32996	CG1242	Heat shock protein 83	VALIUM20
32997	CG7756,CG31449, CG31359,CG5834, CG6489	Heat shock protein cognate 2 | Heat-shock-protein-70Ba | Heat-shock-protein-70Bb | Hsp70Bbb | Heat-shock-protein-70Bc	VALIUM20
32402	CG4147	Heat shock protein cognate 3	VALIUM20
28709	CG4264	Heat shock protein cognate 4	VALIUM10
25794	CG4637	hedgehog	VALIUM10
32489	CG4637	hedgehog	VALIUM20
28991	CG1666	Helicase	VALIUM10
32895	CG4261	Helicase 89B	VALIUM20
33666	CG7269	Helicase at 25E	VALIUM20
33666	CG7269	Helicase at 25E	VALIUM20
25975	CG8522	Helix loop helix protein 106	VALIUM10
26324	CG2655	Helix loop helix protein 3B	VALIUM10
25976	CG3052	Helix loop helix protein 4C	VALIUM10
28710	CG4353	hemipterous	VALIUM10
29406	CG5837	HEM-protein	VALIUM10
33385	CG12367	Hen1	VALIUM20
29540	CG7399	Henna	VALIUM10
28618	CG33147	Heparan sulfate 3-O sulfotransferase-A	VALIUM10
28026	CG2903	Hepatocyte growth factor regulated tyrosine kinase substrate	VALIUM10
28964	CG2903	Hepatocyte growth factor regulated tyrosine kinase substrate	VALIUM10
29375	CG9310	Hepatocyte nuclear factor 4	VALIUM10
27040	CG31000	hephaestus	VALIUM10
27654	CG5927	HES-related	VALIUM10
32401	CG7041	Heterochromatin Protein 1b	VALIUM20
33716	CG10377	Heterogeneous nuclear ribonucleoprotein at 27C	VALIUM20
32351	CG9983	Heterogeneous nuclear ribonucleoprotein at 98DE	VALIUM20
28561	CG3095	hfw	VALIUM10
29382	CG13475	HGTX	VALIUM10
28031	CG32592	highwire	VALIUM10
33378	CG9854	hiiragi	VALIUM20
28376	CG2040	hikaru genki	VALIUM10
27661	CG11228	hippo	VALIUM10
33614	CG11228	hippo	VALIUM20
28966	CG5499	His2Av	VALIUM10
28013	CG14723	Histamine-gated chloride channel subunit 1	VALIUM10

26000	CG3454	Histidine decarboxylase	VALIUM10
32480	CG31119	Histone deacetylase X	VALIUM20
32892	CG13363	Histone methyltransferase 4-20	VALIUM20
28698	CG5005	HLH54F	VALIUM10
26219	CG17921	HMG protein Z	VALIUM10
33377	CG12787	hoepel1	VALIUM20
28661	CG15624	hoepel2	VALIUM10
27271	CG11324	homer	VALIUM10
32466	CG4879	homolog of RecQ	VALIUM20
27655	CG17117	homothorax	VALIUM10
32966	CG1594	hopscotch	VALIUM20
29376	CG1864	Hormone receptor-like in 38	VALIUM10
29377	CG1864	Hormone receptor-like in 38	VALIUM10
27086	CG8676	Hormone receptor-like in 39	VALIUM10
33624	CG8676	Hormone receptor-like in 39	VALIUM20
27253	CG33183	Hormone receptor-like in 46	VALIUM10
27254	CG33183	Hormone receptor-like in 46	VALIUM10
27992	CG11783	Hormone receptor-like in 96	VALIUM10
32453	CG1710	Host cell factor	VALIUM20
28603	CG9854	hrg	VALIUM10
32900	CG2947	Hsc/Hsp70-interacting protein related	VALIUM20
33742	CG6603	Hsc70Cb	VALIUM20
32979	CG2720	Hsp70/Hsp90 organizing protein homolog	VALIUM20
28787	CG5834	Hsp70Bbb	VALIUM10
33000	CG5834	Hsp70Bbb	VALIUM20
28544	CG8464	HtrA2	VALIUM10
28705	CG6371	hugin	VALIUM10
29630	CG9786	hunchback	VALIUM10
32504	CG10971	Huntingtin interacting protein 1	VALIUM20
28330	CG32688	Hyperkinetic	VALIUM10
32352	CG9484	hyperplastic discs	VALIUM20
29574	CG8585	I[[h]] channel	VALIUM10
33672	CG31795	IA-2 ortholog	VALIUM20
32403	CG7788	Ice	VALIUM20
29428	CG33527	IFamide	VALIUM10
29598	CG18285	igloo	VALIUM10
28518	CG18279	IM10	VALIUM10
32845	CG8625	Imitation SWI	VALIUM20
28788	CG18106	Immune induced molecule 2	VALIUM10
25865	CG4536	inactive	VALIUM10
32514	CG9078	infertile crescent	VALIUM20
27544	CG9623	inflated	VALIUM10
28915	CG4590	innexin 2	VALIUM10
26297	CG2977	innexin 7	VALIUM10
33708	CG31212	Ino80	VALIUM20
25937	CG1063	Inositol 1,4,5,-tris-phosphate receptor	VALIUM10
28028	CG3028	Inositol polyphosphate 1-phosphatase	VALIUM10
32861	CG14173	Insulin-like peptide 1	VALIUM20

32475	CG8167	Insulin-like peptide 2	VALIUM20
33681	CG14167	Insulin-like peptide 3	VALIUM20
33682	CG6736	Insulin-like peptide 4	VALIUM20
33683	CG33273	Insulin-like peptide 5	VALIUM20
33684	CG14049	Insulin-like peptide 6	VALIUM20
32862	CG13317	Insulin-like peptide 7	VALIUM20
33358	CG4785	Integrator 14	VALIUM20
33048	CG5859	Integrator 8	VALIUM20
28662	CG11551	intermediate neuroblasts defective	VALIUM10
32411	CG10228	Inverse regulator a	VALIUM20
29594	CG5247	Inverted repeat-binding protein	VALIUM10
25823	CG6747	Inwardly rectifying potassium channel	VALIUM10
25820	CG4370	Inwardly rectifying potassium channel 2	VALIUM10
26720	CG10369	Inwardly rectifying potassium channel 3	VALIUM10
29306	CG4590	inx2	VALIUM10
28042	CG7537	inx5	VALIUM10
25799	CG13586	ion transport peptide	VALIUM10
29539	CG15627	Ionotropic receptor 25a	VALIUM10
25813	CG32704	Ionotropic receptor 8a	VALIUM10
28691	CG6620	IplI-aurora-like kinase	VALIUM10
28064	CG5695	jaguar	VALIUM10
27024	CG9397	jing	VALIUM10
32975	CG8165	JmjC domain-containing histone demethylase 2	VALIUM20
32891	CG3654	Jumonji, AT rich interactive domain 2	VALIUM20
26010	CG18039	KaiRIA	VALIUM10
27523	CG8548	karyopherin α1	VALIUM10
27535	CG9423	karyopherin α3	VALIUM10
28375	CG10229	katanin 60	VALIUM10
32506	CG1193	katanin p60-like 1	VALIUM20
27722	CG33956	kayak	VALIUM10
33379	CG33956	kayak	VALIUM20
27252	CG33135	KCNQ potassium channel	VALIUM10
32937	CG2899	kinase suppressor of ras	VALIUM20
25898	CG7765	Kinesin heavy chain	VALIUM10
33685	CG9191	Kinesin-like protein at 61F	VALIUM20
27549	CG10923	Kinesin-like protein at 67A	VALIUM10
29410	CG7293	Kinesin-like protein at 68D	VALIUM10
28313	CG17046	klarsicht	VALIUM10
28746	CG6669	klingon	VALIUM10
28731	CG12296	klumpfuss	VALIUM10
27259	CG4717	knirps	VALIUM10
27248	CG2922	exba	VALIUM10
27666	CG3340	Kruppel	VALIUM10
29523	CG1487	krz	VALIUM10
27710	CG18801	Ku80	VALIUM10
28750	CG5175	kugelkern	VALIUM10
28594	CG5504	l(2)tid	VALIUM10
28979	CG4195	l(3)73Ah	VALIUM10

32353	CG12794	la costa	VALIUM20
26753	CG1264	labial	VALIUM10
28940	CG12369	Lac	VALIUM10
28374	CG6545	ladybird early	VALIUM10
28071	CG10236	Laminin A	VALIUM10
27036	CG10255	Lap1	VALIUM10
27703	CG8597	lark	VALIUM10
32455	CG2044	Larval cuticle protein 4	VALIUM20
27042	CG4178	Larval serum protein 1 β	VALIUM10
26305	CG3849	Lasp	VALIUM10
27278	CG2374	late bloomer	VALIUM10
25876	CG4088	latheo	VALIUM10
32515	CG11440	lazaro	VALIUM20
28903	CG8434	lbk	VALIUM10
27317	CG5481	leak	VALIUM10
33380	CG1639	lethal (1) 10Bb	VALIUM20
33380	CG1639	lethal (1) 10Bb	VALIUM20
32910	CG2221	lethal (1) G0289	VALIUM20
33690	CG2221	lethal (1) G0289	VALIUM20
32404	CG10691	lethal (2) 37Cc	VALIUM20
32912	CG10691	lethal (2) 37Cc	VALIUM20
27729	CG8732	lethal (2) 44DEa	VALIUM10
27311	CG4713	lethal (2) giant discs 1	VALIUM10
33002	CG8426	lethal (2) NC136	VALIUM20
32938	CG4084	lethal (2) neighbor of tid	VALIUM20
32479	CG4195	lethal (3) 73Ah	VALIUM20
32454	CG7832	lethal (3) L1231	VALIUM20
32962	CG12755	lethal (3) malignant blood neoplasm	VALIUM20
28076	CG5954	lethal (3) malignant brain tumor	VALIUM10
27058	CG3839	lethal of scute	VALIUM10
32457	CG5483	Leucine-rich repeat kinase	VALIUM20
25798	CG13480	Leucokinin	VALIUM10
25936	CG10626	Leucokinin receptor	VALIUM10
28789	CG31096	Lgr3	VALIUM10
28002	CG8024	lightoid	VALIUM10
28358	CG2520	like-AP180	VALIUM10
26314	CG8817	lilliputian	VALIUM10
29341	CG11354	Lim1	VALIUM10
26227	CG10699	Lim3	VALIUM10
26294	CG1848	LIMK1	VALIUM10
28948	CG1848	LIM-kinase1	VALIUM10
28790	CG32171	Limpet	VALIUM10
29520	CG1877	lin19	VALIUM10
29564	CG17334	lin-28	VALIUM10
28925	CG6113	Lip4	VALIUM10
32846	CG9057	Lipid storage droplet-2	VALIUM20
27522	CG8532	liquid facets	VALIUM10
28663	CG8440	Lissencephaly-1	VALIUM10

27532	CG9088	little imaginal discs	VALIUM10
28944	CG9088	little imaginal discs	VALIUM10
28357	CG17342	Lk6	VALIUM10
32456	CG5248	locomotion defects	VALIUM20
29340	CG3576	Longevity assurance gene 1	VALIUM10
26714	CG12052	longitudinals lacking	VALIUM10
28963	CG6866	loquacious	VALIUM10
32955	CG6866	loquacious	VALIUM20
33407	CG6866	loquacious	VALIUM20
33427	CG6866	loquacious	VALIUM20
27985	CG1689	lozenge	VALIUM10
27249	CG31094	LpR1	VALIUM10
28959	CG6098	Lrr47	VALIUM10
28953	CG33677	LSm-4	VALIUM10
29537	CG33677(CG17768)	LSm-4	VALIUM10
27084	CG33473	luna	VALIUM10
33699	CG11033	Lysine (K)-specific demethylase 2	VALIUM20
32967	CG12141	Lysyl-tRNA synthetase	VALIUM20
25986	CG9954	maf-S	VALIUM10
25792	CG30388	Magi	VALIUM10
33411	CG30388	Magi	VALIUM20
28931	CG9401	mago	VALIUM10
33698	CG3025	males absent on the first	VALIUM20
32849	CG3354	Male-specific transcript 77F	VALIUM20
32376	CG30361	mangetout	VALIUM20
33709	CG3753	Marcal1	VALIUM20
28046	CG8118	mastermind	VALIUM10
33675	CG4916	maternal expression at 31B	VALIUM20
29328	CG9648	Max	VALIUM10
27029	CG10042	MBD-R2	VALIUM10
28550	CG4143	mbf1	VALIUM10
28566	CG4916	me31B	VALIUM10
28572	CG1793	MED26	VALIUM10
32517	CG4184	Mediator complex subunit 15	VALIUM20
27559	CG5546	Mediator complex subunit 19	VALIUM10
33710	CG5546	Mediator complex subunit 19	VALIUM20
33595	CG12254	Mediator complex subunit 25	VALIUM20
32459	CG5121	Mediator complex subunit 28	VALIUM20
33743	CG9473	Mediator complex subunit 6	VALIUM20
33678	CG42517,CG42518	Mediator complex subunit 9, CG42518	VALIUM20
32941	CG8274	Megator	VALIUM20
28587	CG7717	Mekk1	VALIUM10
33676	CG42245,CG1244	MEP-1	VALIUM20
28007	CG14228	Merlin	VALIUM10
32460	CG11100	Mes2	VALIUM20
28353	CG15274	metabotropic GABA-B receptor subtype 1	VALIUM10
27699	CG6706	metabotropic GABA-B receptor subtype 2	VALIUM10

26729	CG3022	metabotropic GABA-B receptor subtype 3	VALIUM10
25938	CG11144	metabotropic glutamate receptor	VALIUM10
33381	CG3743	Metal response element-binding Transcription Factor-1	VALIUM20
33381	CG3743	Metal response element-binding Transcription Factor-1	VALIUM20
26205	CG1705	Methoprene-tolerant	VALIUM10
27495	CG6936	methuselah	VALIUM10
33419	CG8103	Mi-2 ortholog	VALIUM20
28595	CG11259	MICAL-like	VALIUM10
27723	CG7109	microtubule star	VALIUM10
32939	CG1483	Microtubule-associated protein 205	VALIUM20
32458	CG1825	Microtubule-associated protein 60	VALIUM20
28622	CG5588	Mig-2-like	VALIUM10
28385	CG13777	milton	VALIUM10
27320	CG5841	mind bomb 1	VALIUM10
32867	CG4978	Minichromosome maintenance 7	VALIUM20
29415	CG2674	Minute (2) 21AB	VALIUM10
28067	CG1221	miple	VALIUM10
32356	CG12249	miranda	VALIUM20
29601	CG1424	misato	VALIUM10
29380	CG15669	Misexpression suppressor of KSR 2	VALIUM10
28791	CG16973	misshapen	VALIUM10
28294	CG6851	Mitochondrial carrier homolog 1	VALIUM10
27695	CG5410	Mitochondrial Rho	VALIUM10
26744	CG4217	mitochondrial transcription factor A	VALIUM10
28054	CG7319	Mitochondrial Transcription Factor B1	VALIUM10
27055	CG3910	mitochondrial transcription factor B2	VALIUM10
32873	CG4389	Mitochondrial trifunctional protein alpha subunit	VALIUM20
32940	CG11482	Mlh1	VALIUM20
27059	CG18362	Mlx interactor	VALIUM10
28888	CG42273	mnb	VALIUM10
27994	CG11799	Mnf	VALIUM10
29329	CG13316	Mnt	VALIUM10
29567	CG13852	mob as tumor suppressor	VALIUM10
32995	CG32491	modifier of mdg4	VALIUM20
28314	CG2050	modulo	VALIUM10
29429	CG4482	moladietz	VALIUM10
27572	CG7935	moleskin	VALIUM10
33626	CG7935	moleskin	VALIUM20
27316	CG5475	Mpk2	VALIUM10
25881	CG1216	mrityu	VALIUM10
32847	CG1810	mRNA-capping-enzyme	VALIUM20
29460	CG10145	M-spondin	VALIUM10
28526	CG1149	MstProx	VALIUM10
33745	CG2244	MTA1-like	VALIUM20
29528	CG9160	mtacp1	VALIUM10
28546	CG8175	Mtk	VALIUM10

32405	CG3879	Multi drug resistance 49	VALIUM20
25970	CG12124	multi sex combs	VALIUM10
32446	CG12124	multi sex combs	VALIUM20
28664	CG10181	Multiple drug resistance 65	VALIUM10
27543	CG1771	multiple edematous wings	VALIUM10
33046	CG31873	Multi-substrate lipid kinase	VALIUM20
27571	CG4356	muscarinic Acetylcholine Receptor 60C	VALIUM10
29381	CG33149	Muscle LIM protein at 60A	VALIUM10
29585	CG33197	muscleblind	VALIUM10
32377	CG42768	Muscle-specific protein 300	VALIUM20
32848	CG42768	Muscle-specific protein 300	VALIUM20
29379	CG18582	mushroom bodies tiny	VALIUM10
33043	CG9193	mutagen-sensitive 209	VALIUM20
26237	CG9045	Myb oncogene-like	VALIUM10
32461	CG6061	Myb-interacting protein 120	VALIUM20
32462	CG3480	Myb-interacting protein 130	VALIUM20
32834	CG15119	Myb-interacting protein 40	VALIUM20
32355	CG10379	myoblast city	VALIUM20
33722	CG10379	myoblast city	VALIUM20
28699	CG1429	Myocyte enhancing factor 2	VALIUM10
26246	CG6456	Myoinhibiting peptide precursor	VALIUM10
32916	CG9311	myopic	VALIUM20
27547	CG5596	Myosin alkali light chain 1	VALIUM10
32516	CG32156	Myosin binding subunit	VALIUM20
26299	CG17927	Myosin heavy chain	VALIUM10
27735	CG1560	myospheroid	VALIUM10
33642	CG1560	myospheroid	VALIUM20
26012	CG34405	Na channel protein 60E	VALIUM10
28073	CG5670	Na pump alpha subunit	VALIUM10
32913	CG5670	Na pump α subunit	VALIUM20
33646	CG5670	Na pump α subunit	VALIUM20
28306	CG5685	Na/Ca-exchange protein	VALIUM10
27262	CG8178	Nach	VALIUM10
32998	CG6343	NADH dehydrogenase (ubiquinone) 42 kDa subunit	VALIUM20
28894	CG6343	NADH:ubiquinone reductase 42kD subunit precursor	VALIUM10
27739	CG2286	NADH:ubiquinone reductase 75kD subunit precursor	VALIUM10
32433	CG34399	NADPH oxidase	VALIUM20
32902	CG34399	NADPH oxidase	VALIUM20
28300	CG5637	nanos	VALIUM10
32985	CG5637	nanos	VALIUM20
25808	CG1517	narrow abdomen	VALIUM10
27302	CG3845	NAT1	VALIUM10
32357	CG3845	NAT1	VALIUM20
27246	CG18660	Nckx30C	VALIUM10
28897	CG10718	neb	VALIUM10
32963	CG8091	Nedd2-like caspase	VALIUM20

32897	CG5874	Negative elongation factor A	VALIUM20
32835	CG5994	Negative elongation factor E	VALIUM20
27693	CG5125	neither inactivation nor afterpotential C	VALIUM10
27724	CG15319	nejire	VALIUM10
28600	CG17256	Nek2	VALIUM10
25793	CG7892	nemo	VALIUM10
27685	CG33101	NEM-sensitive fusion protein 2	VALIUM10
28551	CG12809	nerfin-2	VALIUM10
28666	CG9261	nervana 2	VALIUM10
29431	CG8663	nervana 3	VALIUM10
28324	CG13906	nervous fingers 1	VALIUM10
27713	CG4684	nervous wreck	VALIUM10
29413	CG3385	nervy	VALIUM10
26204	CG11450	net	VALIUM10
25861	CG10521	Netrin-B	VALIUM10
33686	CG11661	Neural conserved at 73EF	VALIUM20
26023	CG11988	neuralized	VALIUM10
27502	CG7050	Neurexin 1	VALIUM10
32408	CG7050	Neurexin 1	VALIUM20
28715	CG6827	Neurexin IV	VALIUM10
32424	CG6827	Neurexin IV	VALIUM20
29461	CG7641	Neurocalcin	VALIUM10
25845	CG8318	Neurofibromin 1	VALIUM10
28724	CG1634	Neuroglian	VALIUM10
28331	CG13772	neuroligin	VALIUM10
27237	CG10342	neuropeptide F	VALIUM10
25939	CG1147	neuropeptide F receptor	VALIUM10
27507	CG7395	Neuropeptide F-like Receptor 76F	VALIUM10
25944	CG5811	Neuropeptide Y receptor-like	VALIUM10
25872	CG3441	Neuropeptide-like precursor 1	VALIUM10
29430	CG11051	Neuropeptide-like precursor 2	VALIUM10
28760	CG13061	Neuropeptide-like precursor 3	VALIUM10
28793	CG15361	Neuropeptide-like precursor 4	VALIUM10
28742	CG9704	Neurotactin	VALIUM10
28589	CG12178	Nhe1	VALIUM10
27498	CG7012	nicastrin	VALIUM10
29402	CG13645	Nicotinamide mononucleotide adenylytransferase	VALIUM10
27671	CG2302	nicotinic Acetylcholine Receptor alpha 7E	VALIUM10
28688	CG5610	nicotinic Acetylcholine Receptor alpha 96Aa	VALIUM10
27493	CG6844	nicotinic Acetylcholine Receptor alpha 96Ab	VALIUM10
25927	CG11822	nicotinic acetylcholine receptor beta 21C	VALIUM10
28038	CG6798	nicotinic Acetylcholine Receptor beta 96A	VALIUM10
25835	CG4128	nicotinic Acetylcholine Receptor α 30D	VALIUM10
25943	CG32975	nicotinic Acetylcholine Receptor α 34E	VALIUM10
25787	CG8942	nimrod C1	VALIUM10
25960	CG18146	nimrod C2	VALIUM10
28961	CG17704	Nipped-B	VALIUM10
32406	CG17704	Nipped-B	VALIUM20

28792	CG6713	Nitric oxide synthase	VALIUM10
25941	CG2902	NMDA receptor 1	VALIUM10
26019	CG33513	NMDA receptor 2	VALIUM10
28361	CG3798	N-methyl-D-aspartate receptor-associated protein	VALIUM10
27990	CG13207	no mechanoreceptor potential A	VALIUM10
28665	CG12548	no mechanoreceptor potential B	VALIUM10
29370	CG4491	no ocelli	VALIUM10
32407	CG32172	noe	VALIUM20
28725	CG4166	non-stop	VALIUM10
33694	CG7421	Nopp140	VALIUM20
32836	CG34407	Not1	VALIUM20
27988	CG3936	Notch	VALIUM10
28981	CG3936	Notch	VALIUM10
33611	CG3936	Notch	VALIUM20
28037	CG6698	NtR	VALIUM10
28338	CG6246	nubbin	VALIUM10
27573	CG33991	nuclear fallout	VALIUM10
28633	CG1740	Nuclear transport factor-2	VALIUM10
27553	CG10174	Nuclear transport factor-2-related	VALIUM10
32501	CG18271	nuclease slx1	VALIUM20
33688	CG7917	Nucleoplasmin	VALIUM20
32837	CG4453	Nucleoporin 153	VALIUM20
32391	CG4738	Nucleoporin 160	VALIUM20
33003	CG11856	Nucleoporin 358	VALIUM20
32942	CG8722	Nucleoporin 44A	VALIUM20
29622	CG3983	nucleostemin 1	VALIUM10
33382	CG9710	nudC	VALIUM20
28562	CG10198	Nup98	VALIUM10
25923	CG17390	O/E-associated zinc finger protein	VALIUM10
28794	CG2297	Obp44a	VALIUM10
29342	CG12154	ocelliless	VALIUM10
28295	CG3851	odd skipped	VALIUM10
28050	CG9880	Odorant receptor 23a	VALIUM10
29302	CG3206	Odorant receptor 2a	VALIUM10
26295	CG1854	Odorant receptor 43a	VALIUM10
27274	CG13206	Odorant receptor 47b	VALIUM10
27683	CG32401	Odorant receptor 65a	VALIUM10
27289	CG32402	Odorant receptor 65b	VALIUM10
29584	CG32403	Odorant receptor 65c	VALIUM10
27276	CG15581	Odorant receptor 83c	VALIUM10
27038	CG14360	Odorant receptor 88a	VALIUM10
25790	CG8967	off-track	VALIUM10
28916	CG8967	off-track	VALIUM10
33707	CG3736	okra	VALIUM20
29547	CG12673	olf413	VALIUM10
25979	CG5545	Olig family	VALIUM10
29343	CG1922	onecut	VALIUM10
25843	CG10868	oo18 RNA-binding protein	VALIUM10

25885	CG1615	Open rectifier K+ channel 1	VALIUM10
32358	CG8479	optic atrophy 1-like	VALIUM20
27283	CG3039	optic ganglion reduced	VALIUM10
25824	CG7411	ora transientless	VALIUM10
28347	CG3029	orange	VALIUM10
27050	CG5735	orb2	VALIUM10
32409	CG2917	Origin recognition complex subunit 4	VALIUM20
25844	CG10901	oskar	VALIUM10
28722	CG5993	outstretched	VALIUM10
33680	CG5993	outstretched	VALIUM20
28720	CG9881	p16-ARC	VALIUM10
29405	CG7393	p38b	VALIUM10
29351	CG33336	p53	VALIUM10
28945	CG10295	Pak	VALIUM10
25796	CG10118	pale	VALIUM10
25952	CG12108	Palmitoyl-protein thioesterase 1	VALIUM10
28362	CG4851	Palmitoyl-protein Thioesterase 2	VALIUM10
26743	CG34403	pangolin	VALIUM10
33697	CG3978	pannier	VALIUM20
33744	CG3978	pannier	VALIUM20
32410	CG8201	par-1	VALIUM20
26212	CG17888	PAR-domain protein 1	VALIUM10
26293	CG1800	partner of drosha	VALIUM10
27516	CG16765	pasilla	VALIUM10
33426	CG42670	pasilla	VALIUM20
28795	CG2411	patched	VALIUM10
26282	CG12021	Patj	VALIUM10
28695	CG31794	Paxillin	VALIUM10
32886	CG31453	pch2	VALIUM20
28552	CG3443	pcx	VALIUM10
26285	CG13758	PDF receptor	VALIUM10
29592	CG8844	Pdsw	VALIUM10
27712	CG8705	peanut	VALIUM10
32838	CG8241	peanuts	VALIUM20
28343	CG8114	pebble	VALIUM10
28735	CG12212	pebbled	VALIUM10
28735	CG12212	pebbled	VALIUM20
27692	CG4799	Pendulin	VALIUM10
32943	CG13374	pepsinogen-like	VALIUM20
32992	CG13374	pepsinogen-like	VALIUM20
33383	CG4432	PEPTİDOGLİKON RECOGNATİON PROTEİN	VALIUM20
32382	CG7660	Peroxinectin-like	VALIUM20
32435	CG7660	Peroxinectin-like	VALIUM20
32497	CG12405	Peroxiredoxin 2540-1	VALIUM20
29432	CG1668	Pheromone-binding protein-related protein 2	VALIUM10
25968	CG12013	PHGPx	VALIUM10
29383	CG9245	Phosphatidylinositol synthase	VALIUM10
33700	CG5383	phosphatidylserine receptor	VALIUM20

28728	CG14940	Phosphodiesterase 1c	VALIUM10
25828	CG8279	Phosphodiesterase 6	VALIUM10
32412	CG42276	Phosphodiesterase 9	VALIUM20
28053	CG3127	Phosphoglycerate kinase	VALIUM10
33632	CG3127	Phosphoglycerate kinase	VALIUM20
33633	CG3127	Phosphoglycerate kinase	VALIUM20
33346	CG14816	Phosphoglycerate mutase 5	VALIUM20
26303	CG1721	Phosphoglyceromutase	VALIUM10
32438	CG4574	Phospholipase C at 21C	VALIUM20
33719	CG4574	Phospholipase C at 21C	VALIUM20
32839	CG12110	Phospholipase D	VALIUM20
33384	CG5373	Phosphotidylinositol 3 kinase 59F	VALIUM20
27676	CG11205	photorepair	VALIUM10
29433	CG10108	phyllopod	VALIUM10
27690	CG4141	Pi3K92E	VALIUM10
29571	CG3478	pickpocket	VALIUM10
27256	CG34042	pickpocket 10	VALIUM10
26253	CG34058	pickpocket 11	VALIUM10
25817	CG33508	pickpocket 13	VALIUM10
27091	CG9501	pickpocket 14	VALIUM10
25890	CG34059	pickpocket 16	VALIUM10
25887	CG18287	pickpocket 19	VALIUM10
25897	CG7577	pickpocket 20	VALIUM10
25849	CG12048	pickpocket 21	VALIUM10
28350	CG8527	pickpocket 23	VALIUM10
27088	CG33349	pickpocket 25	VALIUM10
25880	CG11209	pickpocket 6	VALIUM10
25922	CG9499	pickpocket 7	VALIUM10
25920	CG8098	Picot	VALIUM10
25802	CG6496	Pigment-dispersing factor	VALIUM10
28693	CG2368	pipsqueak	VALIUM10
33724	CG6122	piwi	VALIUM20
28911	CG17245	plexB	VALIUM10
28936	CR33938	pncr002:3R	VALIUM10
28957	CR31696	pncr003:2L	VALIUM10
28547	CR33939	pncr004:X	VALIUM10
29526	CR33946	pncr008:3L	VALIUM10
28970	CR33947	pncr011:3L	VALIUM10
28591	CG13303	pncr013:4	VALIUM10
28941	CR33948	pncr015:3L	VALIUM10
28593	CR33945	pncr017:3R	VALIUM10
28935	CG3978	pnr	VALIUM10
32386	CG32885	polar granule component	VALIUM20
33720	CG32885	polar granule component	VALIUM20
33042	CG12306	polo	VALIUM20
25951	CG12085	poly U binding factor 68kD	VALIUM10
28821	CG5119	polyA-binding protein	VALIUM10
32840	CG11375	polybromo	VALIUM20

33386	CG43140	polychaetoid	VALIUM20
33622	CG32443	Polycomb	VALIUM20
33669	CG18412	polyhomeotic proximal	VALIUM20
32463	CG2103	polypeptide GalNAc transferase 6	VALIUM20
29572	CG6647	porin	VALIUM10
25886	CG17137	Porin2	VALIUM10
26749	CG11641	pou domain motif 3	VALIUM10
29543	CG12287	POU domain protein 2	VALIUM10
26757	CG9610	Pox meso	VALIUM10
26238	CG8246	Pox neuro	VALIUM10
27681	CG18803	Presenilin	VALIUM10
27298	CG33198	presenilin enhancer	VALIUM10
32413	CG11084	prickle	VALIUM20
29595	CG31481	proboscipedia	VALIUM10
29570	CG7105	Proctolin	VALIUM10
29414	CG6986	Proctolin receptor	VALIUM10
33695	CG31022	prolyl-4-hydroxylase-alpha EFB	VALIUM20
27284	CG30483	Prosap	VALIUM10
26745	CG17228	prospero	VALIUM10
29385	CG10149	Proteasome p44.5 subunit	VALIUM10
33660	CG1519	Proteasome α7 subunit	VALIUM20
27491	CG6622	Protein C kinase 53E	VALIUM10
29311	CG1954	Protein C kinase 98E	VALIUM10
28039	CG6988	Protein disulfide isomerase	VALIUM10
27725	CG1210	Protein kinase 61C	VALIUM10
32464	CG4720	Protein kinase at 92B	VALIUM20
28355	CG10524	Protein kinase C delta	VALIUM10
28717	CG7125	Protein Kinase D	VALIUM10
28335	CG2049	Protein kinase related to protein kinase N	VALIUM10
28757	CG7001	Protein kinase-like 17E	VALIUM10
32944	CG6143	Protein on ecdysone puffs	VALIUM20
32465	CG9156	Protein phosphatase 1 at 13C	VALIUM20
32414	CG5650	Protein phosphatase 1 at 87B	VALIUM20
27726	CG32505	Protein phosphatase 19C	VALIUM10
29384	CG17291	Protein phosphatase 2A at 29B	VALIUM10
25929	CG9842	Protein phosphatase 2B at 14D	VALIUM10
26296	CG2890	Protein phosphatase 4 regulatory subunit 2-related protein	VALIUM10
32426	CG9181	Protein tyrosine phosphatase 61F	VALIUM20
29462	CG10975	Protein tyrosine phosphatase 69D	VALIUM10
25840	CG11516	Protein tyrosine phosphatase 99A	VALIUM10
32359	CG9856	Protein tyrosine phosphatase-ERK/Enhancer of Ras1	VALIUM20
32971	CG2152	Protein-L-isoaspartate (D-aspartate) O-methyltransferase	VALIUM20
33433	CG43947	Protostome-specific GEF	VALIUM20
32865	CG5519	Prp19	VALIUM20
25841	CG5671	Pten	VALIUM10
25967	CG5671	Pten	VALIUM10

33643	CG5671	Pten	VALIUM20
28973	CG11212	Ptr	VALIUM10
26725	CG9755	pumilio	VALIUM10
27700	CG7758	pumpless	VALIUM10
27514	CG7904	punt	VALIUM10
32945	CG14472	purity of essence	VALIUM20
33689	CG6148	Putative Achaete Scute Target 1	VALIUM20
28934	CG7660	pxt	VALIUM10
28920	CG31349	pyd	VALIUM10
28635	CG8808	Pyruvate dehydrogenase kinase	VALIUM10
29356	CG17521	Qm	VALIUM10
28667	CG14039	quick-to-court	VALIUM10
28047	CG8432	Rab escort protein	VALIUM10
29403	CG17515	Rab21	VALIUM10
28025	CG2108	Rab23	VALIUM10
28342	CG9575	Rab35	VALIUM10
25953	CG12156	Rab39	VALIUM10
28954	CG5627	rab3-GEF	VALIUM10
29320	CG5627	rab3-GEF	VALIUM10
29579	CG1900	Rab40	VALIUM10
25950	CG11556	Rabphilin	VALIUM10
27299	CG3320	Rab-protein 1	VALIUM10
26289	CG17060	Rab-protein 10	VALIUM10
27730	CG5771	Rab-protein 11	VALIUM10
28708	CG4212	Rab-protein 14	VALIUM10
28701	CG3269	Rab-protein 2	VALIUM10
27490	CG6601	Rab-protein 6	VALIUM10
27051	CG5915	Rab-protein 7	VALIUM10
27519	CG8287	Rab-protein 8	VALIUM10
27665	CG3129	Rab-related protein 4	VALIUM10
32360	CG2885	RabX2	VALIUM20
28704	CG31118	RabX4	VALIUM10
28045	CG7980	RabX5	VALIUM10
26281	CG12015	RabX6	VALIUM10
28985	CG2248	Rac1	VALIUM10
32882	CG9862	Rae1	VALIUM20
32342	CG8707	RagC	VALIUM20
33389	CG8865	Ral guanine nucleotide dissociation stimulator-like ortholog (M. musculus)	VALIUM20
29565	CG9999	Ran GTPase activating protein	VALIUM10
33004	CG5252	Ranbp9	VALIUM20
33005	CG5252	Ranbp9	VALIUM20
27512	CG7815	ran-like	VALIUM10
27663	CG4656	Ras association family member	VALIUM10
29318	CG1167	Ras oncogene at 64B	VALIUM10
29319	CG9375	Ras oncogene at 85D	VALIUM10
27520	CG8418	Ras which interacts with Calmodulin	VALIUM10
29568	CG3204	Ras-associated protein 2-like	VALIUM10
28921	CG11495	rasp	VALIUM10

33392	CG9412	rasputin	VALIUM20
29580	CG2849	Ras-related protein	VALIUM10
32354	CG9528,CR43498	real-time | CR43498	VALIUM20
33662	CG18174	Regulatory particle non-ATPase 11	VALIUM20
32874	CG3455	Regulatory particle triple-A ATPase 4	VALIUM20
33661	CG11992	Relish	VALIUM20
32415	CG9750	reptin	VALIUM20
25948	CG14396	Ret oncogene	VALIUM10
26309	CG5403	retained	VALIUM10
29435	CG34344	retinal degeneration A	VALIUM10
28796	CG11111	retinal degeneration B	VALIUM10
28674	CG10052	Retinal Homeobox	VALIUM10
33388	CG11064	Retinoid- and fatty acid-binding glycoprotein	VALIUM20
28339	CG31240	reversed polarity	VALIUM10
28946	CG11064	Rfabg	VALIUM10
29355	CG6312	Rfx	VALIUM10
28938	CG8865	Rgl	VALIUM10
32999	CG6831	rhea	VALIUM20
33425	CG42316	Rho GTPase activating protein at 102A	VALIUM20
33425	CG42316	Rho GTPase activating protein at 102A	VALIUM20
33390	CG40494	Rho GTPase activating protein at 1A	VALIUM20
33391	CG4755	Rho GTPase activating protein at 92B	VALIUM20
27727	CG8416	Rho1	VALIUM10
32383	CG8416	Rho1	VALIUM20
32416	CG5701	RhoBTB	VALIUM20
32946	CG1976	RhoGAP100F	VALIUM20
32361	CG1412	RhoGAP19D	VALIUM20
32417	CG32149	RhoGAP71E	VALIUM20
28797	CG9774	Rho-kinase	VALIUM10
32841	CG9366	rho-like	VALIUM20
33723	CG9366	Rho-like	VALIUM20
28690	CG1004	rhomboid	VALIUM10
32947	CG10043	rho-type guanine exchange factor	VALIUM20
33738	CG34104	RhoU	VALIUM20
32864	CG8975	Ribonucleoside diphosphate reductase small subunit	VALIUM20
33730	CG1381	Ribosomal protein LP0-like	VALIUM20
33393	CG10305	Ribosomal protein S26	VALIUM20
33393	CG10305	Ribosomal protein S26	VALIUM20
32850	CG2998	Ribosomal protein S28b	VALIUM20
32851	CG15697	Ribosomal protein S30	VALIUM20
32418	CG10944	Ribosomal protein S6	VALIUM20
33394	CG3395	Ribosomal protein S9	VALIUM20
27300	CG33547	Rim	VALIUM10
25847	CG1058	ripped pocket	VALIUM10
27734	CR32777	RNA on the X 1	VALIUM10
28986	CR32665	RNA on the X 2	VALIUM10
28669	CG3151	RNA-binding protein 9	VALIUM10
29398	CG5423	robo3	VALIUM10

32362	CG16982	Roc1a	VALIUM20
28929	CG15811	Rop	VALIUM10
28671	CG6348	rough	VALIUM10
29434	CG1956	Roughened	VALIUM10
28672	CG4125	roughest	VALIUM10
32467	CG4336	roughex	VALIUM20
28035	CG5422	Rox8	VALIUM10
32472	CG5422	Rox8	VALIUM20
33725	CG7471	Rpd3	VALIUM20
27731	CG17596	RPS6-protein kinase-II	VALIUM10
32363	CG2173	Rs1	VALIUM20
28574	CG9108	RSG7	VALIUM10
28668	CG11427	ruby	VALIUM10
32477	CG11427	ruby	VALIUM20
26727	CG31152	rumi	VALIUM10
28673	CG1849	runt	VALIUM10
33353	CG34145	RunxA	VALIUM20
27035	CG9533	rutabaga	VALIUM10
29445	CG10844	Ryanodine receptor 44F	VALIUM10
28919	CG10844	Rya-r44F	VALIUM10
33387	CG10998	ryder cup	VALIUM20
29388	CG8717	saliva	VALIUM10
25980	CG12952	salivary gland-expressed bHLH	VALIUM10
28006	CG33193	salvador	VALIUM10
32965	CG33193	salvador	VALIUM20
32364	CG7073	sar1	VALIUM20
27260	CG6072	sarah	VALIUM10
29551	CG5657	Sarcoglycan beta	VALIUM10
25964	CG14808	Sarcoglycan δ	VALIUM10
28924	CG1664	sbr	VALIUM10
27545	CG8095	scab	VALIUM10
28675	CG17579	scabrous	VALIUM10
29352	CG8544	scalloped	VALIUM10
29387	CG17594	scarecrow	VALIUM10
29386	CG11066	scarface	VALIUM10
29550	CG12789	scavenger receptor acting in neural tissue and majority of rhodopsin is absent	VALIUM10
28677	CG16975	Scm-related gene containing four mbt domains	VALIUM10
32473	CG16975	Scm-related gene containing four mbt domains	VALIUM20
27025	CG1130	scratch	VALIUM10
29552	CG5462	scribbled	VALIUM10
27049	CG5580	scribbler	VALIUM10
29437	CG5186	scruin like at the midline	VALIUM10
26206	CG3827	scute	VALIUM10
27483	CG6159	sec10	VALIUM10
32468	CG6773	sec13	VALIUM20
27499	CG7034	sec15	VALIUM10
32365	CG1250	sec23	VALIUM20
32878	CG8266	sec31	VALIUM20

27526	CG8843	sec5	VALIUM10
27314	CG5341	sec6	VALIUM10
32366	CG7578	sec71	VALIUM20
28352	CG32451	Secretory Pathway Calcium atpase	VALIUM10
29553	CG8553	Selenide,water dikinase	VALIUM10
29554	CG18405	Sema-1a	VALIUM10
28588	CG6446	Sema-1b	VALIUM10
29519	CG4700	Sema-2a	VALIUM10
29436	CG5661	Semaphorin-5c	VALIUM10
27287	CG32120	senseless	VALIUM10
27285	CG31632	senseless-2	VALIUM10
33691	CG9188	septin interacting protein 2	VALIUM20
27709	CG1403	Septin-1	VALIUM10
28004	CG4173	Septin-2	VALIUM10
25834	CG16720	Serotonin receptor 1A	VALIUM10
25833	CG15113	Serotonin receptor 1B	VALIUM10
27273	CG12073	Serotonin receptor 7	VALIUM10
28713	CG6127	Serrate	VALIUM10
32419	CG6939	SET domain binding factor	VALIUM20
33704	CG40351	Set1	VALIUM20
33706	CG1716	Set2	VALIUM20
28689	CG11502	seven up	VALIUM10
28676	CG1030	Sex combs reduced	VALIUM10
29522	CG6987	SF2	VALIUM10
32367	CG6987	SF2	VALIUM20
27653	CG6757	SH3PX1	VALIUM10
25805	CG1066	Shaker cognate b	VALIUM10
28346	CG2822	Shaker cognate w	VALIUM10
27291	CG34358	shaking B	VALIUM10
27292	CG34358	shaking B	VALIUM10
27269	CG11049	shaven	VALIUM10
28513	CG18102	shibire	VALIUM10
25867	CG13968	short neuropeptide F precursor	VALIUM10
28336	CG18076	short stop	VALIUM10
27689	CG3722	shotgun	VALIUM10
32428	CG3722	shotgun	VALIUM20
32904	CG3722	shotgun	VALIUM20
28678	CG13701	sickle	VALIUM10
33412	CG5227	sidekick	VALIUM20
25831	CG10823	SIFamide receptor	VALIUM10
27487	CG6521	Signal transducing adaptor molecule	VALIUM10
33637	CG4257	Signal-transducer and activator of transcription protein at 92E	VALIUM20
26207	CG7951	similar	VALIUM10
32368	CG8815	Sin3A	VALIUM20
33397	CG8991	Sine oculis-binding protein	VALIUM20
26739	CG7771	single-minded	VALIUM10
32488	CG7771	single-minded	VALIUM20
32481	CG5216	Sir2	VALIUM20

32482	CG5085	Sirt2	VALIUM20
32483	CG11305	Sirt7	VALIUM20
29330	CG1641	sisterless A	VALIUM10
27715	CG9985	skittles	VALIUM10
28974	CG16983	skpA	VALIUM10
32870	CG16983	skpA	VALIUM20
32991	CG16983	skpA	VALIUM20
32969	CG12819	slender lobes	VALIUM20
32948	CG2272	slipper	VALIUM20
29354	CG16738	sloppy paired 1	VALIUM10
29344	CG6534	slouch	VALIUM10
27043	CG4354	slow border cells	VALIUM10
26247	CG10693	slowpoke	VALIUM10
27492	CG6772	Slowpoke binding protein	VALIUM10
26756	CG2262	Smad on X	VALIUM10
27238	CG10706	small conductance calcium-activated potassium channel	VALIUM10
29463	CG1391	small optic lobes	VALIUM10
32370	CG8427	Small ribonucleoprotein particle protein SmD3	VALIUM20
33664	CG18591	Small ribonucleoprotein particle protein SmE	VALIUM20
32385	CG4200	small wing	VALIUM20
32906	CG4200	small wing	VALIUM20
28887	CG5352	SmB	VALIUM10
32369	CG10212	SMC2	VALIUM20
26288	CG16725	Smn	VALIUM10
27037	CG11561	smoothened	VALIUM10
27068	CG4013	Smr	VALIUM10
28034	CG4494	smt3	VALIUM10
33739	CG5451	SMU1 ortholog	VALIUM20
28679	CG3956	snail	VALIUM10
27541	CG9958	snapin	VALIUM10
25931	CG3051	SNF1A/AMP-activated protein kinase	VALIUM10
32371	CG3051	SNF1A/AMP-activated protein kinase	VALIUM20
26291	CG17299	SNF4/AMP-activated protein kinase gamma subunit	VALIUM10
32372	CG1064	Snf5-related 1	VALIUM20
33434	CG42257	Snipper	VALIUM20
28682	CG4357	sodium chloride cotransporter 69	VALIUM10
29587	CG6625	Soluble NSF attachment protein	VALIUM10
26221	CG3090	Sox box protein 14	VALIUM10
26220	CG11153	Sox102F	VALIUM10
25996	CG18024	SoxNeuro	VALIUM10
32420	CG14112	SoxNeuro Co-Factor	VALIUM20
29555	CG14041	SP555	VALIUM10
32439	CG3595	spaghetti squash	VALIUM20
33714	CG6464	spalt major	VALIUM20
29549	CG4881	spalt-related	VALIUM10
27570	CG5977	spastin	VALIUM10
26208	CG6993	spineless	VALIUM10

33415	CG6993	spineless	VALIUM20
27702	CG8428	spinster	VALIUM10
28387	CG10334	spitz	VALIUM10
28036	CG5836	Splicing factor 1	VALIUM10
33398	CG18497	split ends	VALIUM20
32852	CG10155	Sprouty-related protein with EVH-1 domain	VALIUM20
32896	CG12372	spt4	VALIUM20
32373	CG12225	Spt6	VALIUM20
25788	CG18247	Src homology 2, ankyrin repeat, tyrosine kinase	VALIUM10
28606	CG3992	srp	VALIUM10
29464	CG11115	Ssl1	VALIUM10
26022	CG11895	starry night	VALIUM10
27305	CG40293	Ste20-like kinase	VALIUM10
32374	CG11628	steppke	VALIUM20
25789	CG34418	still life	VALIUM10
28510	CG12473	stoned B	VALIUM10
25807	CG12295	straightjacket	VALIUM10
26736	CG18255	Stretchin-Mlck	VALIUM10
29556	CG1395	string	VALIUM10
27701	CG7847	stripe	VALIUM10
27263	CG9126	Stromal Interaction molecule	VALIUM10
33395	CG3423	Stromalin	VALIUM20
33431	CG9802	Structural maintenance of chromosomes 3	VALIUM20
26222	CG4817	Structure specific recognition protein	VALIUM10
28559	CG6054	Su(fu)	VALIUM10
28900	CG3497	Su(H)	VALIUM10
33399	CG32217	Su(Tpl)	VALIUM20
25972	CG12864	Su(var)2-HP2	VALIUM10
33402	CG8013	Su(z)12	VALIUM20
28570	CG12298	sub	VALIUM10
27026	CG3850	sugarbabe	VALIUM10
29389	CG11793	Superoxide dismutase	VALIUM10
32909	CG11793	Superoxide dismutase	VALIUM20
25969	CG8905	Superoxide dismutase 2 (Mn)	VALIUM10
32496	CG8905	Superoxide dismutase 2 (Mn)	VALIUM20
32983	CG8905	Superoxide dismutase 2 (Mn)	VALIUM20
32474	CG4086	Suppressor of ref(2)P sterility	VALIUM20
33400	CG8409	Suppressor of variegation 205	VALIUM20
29448	CG8068	Suppressor of variegation 2-10	VALIUM10
32915	CG8068	Suppressor of variegation 2-10	VALIUM20
32956	CG8068	Suppressor of variegation 2-10	VALIUM20
32853	CG17149	Suppressor of variegation 3-3	VALIUM20
33726	CG17149	Suppressor of variegation 3-3	VALIUM20
32914	CG43664,CG43665	Suppressor of variegation 3-9 | Eukaryotic initiation factor 2gamma	VALIUM20
33401	CG43664,CG43665 Eukaryotic initiation factor 2γ	Suppressor of variegation 3-9, Eukaryotic initiation factor 2γ	VALIUM20
33403	CG3905	Suppressor of zeste 2	VALIUM20

29557	CG5407	Sur-8	VALIUM10
28548	CG10084	swm	VALIUM10
28719	CG9474	Synapse protein 24	VALIUM10
27306	CG40452	Synapse protein 25	VALIUM10
27527	CG8884	Synapse-associated protein 47kD	VALIUM10
27304	CG3985	Synapsin	VALIUM10
27489	CG6562	synaptojanin	VALIUM10
28508	CG10617	Synaptotagmin 12	VALIUM10
28365	CG9778	Synaptotagmin 14	VALIUM10
26730	CG10047	Synaptotagmin IV	VALIUM10
29308	CG5559	Synaptotagmin α	VALIUM10
27297	CG33094	Syndapin	VALIUM10
25884	CG1467	Syntaxin 16	VALIUM10
25896	CG7452	Syntaxin 17	VALIUM10
26721	CG13626	Syntaxin 18	VALIUM10
25811	CG31136	Syntaxin 1A	VALIUM10
29397	CG4214	Syntaxin 5	VALIUM10
28505	CG7736	Syntaxin 6	VALIUM10
29546	CG5081	Syntaxin 7	VALIUM10
26013	CG4109	Syntaxin 8	VALIUM10
27504	CG7152	Syntrophin-like 1	VALIUM10
28363	CG4905	Syntrophin-like 2	VALIUM10
27279	CG2381	Syt7	VALIUM10
25800	CG14734	Tachykinin	VALIUM10
27513	CG7887	Tachykinin-like receptor at 99D	VALIUM10
28971	CG13109	tai	VALIUM10
27242	CG1378	tailless	VALIUM10
32885	CG13109	taiman	VALIUM20
29417	CG7417	TAK1-associated Binding Protein2	VALIUM10
26740	CG11987	tango	VALIUM10
32855	CG6586	tantalus	VALIUM20
26209	CG7659	target of Poxn	VALIUM10
28015	CG9637	Task6	VALIUM10
27264	CG9361	Task7	VALIUM10
32422	CG10370	Tat-binding protein-1	VALIUM20
32421	CG17603	TBP-associated factor 1	VALIUM20
32854	CG5374	Tcp1-like	VALIUM20
28022	CG1374	teashirt	VALIUM10
29533	CG7121	Tehao	VALIUM10
26249	CG1232	temperature-induced paralytic E	VALIUM10
29439	CG32659	Tenascin accessory	VALIUM10
29390	CG5723	Tenascin major	VALIUM10
32869	CG2331	TER94	VALIUM20
29440	CG33950, CG7981	terribly reduced optic lobes	VALIUM10
25829	CG8666	Tetraspanin 39D	VALIUM10
29392	CG12143	Tetraspanin 42Ej	VALIUM10
33404	CG18492	TGF-β activated kinase 1	VALIUM20
32856	CG18096	Thioester-containing protein 1	VALIUM20

32498	CG1633	thioredoxin peroxidase 1	VALIUM20
32984	CG2151	Thioredoxin reductase-1	VALIUM20
33721	CG31884	thioredoxin-2	VALIUM20
28537	CG31671	tho2	VALIUM10
29399	CG11326	Thrombospondin	VALIUM10
29583	CG3234	timeless	VALIUM10
28539	CG7895	tin	VALIUM10
28563	CG6121	Tip60	VALIUM10
28543	CG18241	Toll-4	VALIUM10
28519	CG6890	Tollo	VALIUM10
29573	CG8330	tomboy40	VALIUM10
29372	CG7958	tonalli	VALIUM10
28886	CG6064	TORC	VALIUM10
33627	CG1389	torso	VALIUM20
33627	CG1389	torso	VALIUM20
29391	CG14620	touch insensitive larva B	VALIUM10
27986	CG6883	trachealess	VALIUM10
33612	CG42865	trachealess	VALIUM20
25987	CG10034	traffic jam	VALIUM10
28542	CG10686	tral	VALIUM10
26315	CG1856	tramtrack	VALIUM10
28512	CG16724	transformer	VALIUM10
28018	CG10128	transformer 2	VALIUM10
32911	CG4800	Translationally controlled tumor protein	VALIUM20
26005	CG12157	Translocase of outer membrane 40	VALIUM10
32879	CG8309	Transport and Golgi organization 7	VALIUM20
27546	CG7398	Transportin	VALIUM10
25988	CG2848	Transportin-Serine/Arginine rich	VALIUM10
33718	CG3171	Trapped in endoderm 1	VALIUM20
27672	CG3171	trapped in endoderm-1	VALIUM10
28670	CG8085	tre oncogene-related protein	VALIUM10
28326	CG8637	tricornered	VALIUM10
27732	CG18214	trio	VALIUM10
26304	CG2171	Triose phosphate isomerase	VALIUM10
33703	CG8651	trithorax	VALIUM20
29563	CG3848	trithorax-related	VALIUM10
28525	CG11280	trn	VALIUM10
27053	CG2981	Troponin C at 41C	VALIUM10
26172	CG9073	Troponin C at 47D	VALIUM10
26722	CG18345	trp-like	VALIUM10
25842	CG9122	Tryptophan hydroxylase	VALIUM10
28515	CG4591	Tsp86D	VALIUM10
28955	CG8781	tsu	VALIUM10
28982	CG13345	tumbleweed	VALIUM10
32490	CG31137	twin	VALIUM20
32901	CG31137	twin	VALIUM20
29346	CG11186	twin of eyeless	VALIUM10
33679	CG11186	twin of eyeless	VALIUM20

29345	CG10704	twin of eyg	VALIUM10
33044	CG4965	twine	VALIUM20
28714	CG6235	twins	VALIUM10
33670	CG4254	twinstar	VALIUM20
25981	CG2956	twist	VALIUM10
28332	CG7485	Tyramine receptor	VALIUM10
27670	CG16766	Tyramine receptor II	VALIUM10
27667	CG1543	Tyramine β hydroxylase	VALIUM10
25801	CG30445	Tyrosine decarboxylase 1	VALIUM10
25871	CG30446	Tyrosine decarboxylase 2	VALIUM10
29304	CG3582	U2 small nuclear riboprotein auxiliary factor 38	VALIUM10
27542	CG9998	U2 small nuclear riboprotein auxiliary factor 50	VALIUM10
33671	CG1406	U2A	VALIUM20
28569	CG7528	Uba2	VALIUM10
25957	CG1782	Ubiquitin activating enzyme 1	VALIUM10
33405	CG11624	Ubiquitin-63E	VALIUM20
27558	CG5505	Ubiquitin-specific protease 36	VALIUM10
25862	CG11173	ubisnap	VALIUM10
28545	CR32957	Uhg3	VALIUM10
28951	CG8566	unc-104	VALIUM10
29548	CG2999	unc-13	VALIUM10
32859	CG33542	unpaired 3	VALIUM20
29393	CG1650	unplugged	VALIUM10
29558	CG3533	unzipped	VALIUM10
28575	CG33542	upd3	VALIUM10
32949	CG7107	upheld	VALIUM20
29334	CG7015	Upstream of N-ras	VALIUM10
32432	CG7015	Upstream of N-ras	VALIUM20
32491	CG7015	Upstream of N-ras	VALIUM20
25982	CG17592	Usf	VALIUM10
32950	CG2762	u-shaped	VALIUM20
27684	CG32848	VAChT	VALIUM10
29394	CG9209	vacuolar peduncle	VALIUM10
26286	CG14750	Vacuolar protein sorting 25	VALIUM10
27312	CG5014	Vap-33-1	VALIUM10
28599	CG9326	vari	VALIUM10
32434	CG43081	vasa	VALIUM20
29590	CG7662	veli	VALIUM10
32389	CG10107	veloren	VALIUM20
27733	CG6172	ventral nervous system defective	VALIUM10
26228	CG10037	ventral veins lacking	VALIUM10
27538	CG9887	Vesicular glutamate transporter	VALIUM10
26290	CG1709	Vha100-1	VALIUM10
26004	CG11589	VhaM9.7-1	VALIUM10
25965	CG3299	Vinculin	VALIUM10
32951	CG3572	visceral mesodermal armadillo-repeats	VALIUM20
29544	CG8821	vismay	VALIUM10
25989	CG14029	vrille	VALIUM10

26223	CG33980	Vsx2	VALIUM10
27525	CG8789	wallenda	VALIUM10
27662	CG12072	warts	VALIUM10
25955	CG1520	WASp	VALIUM10
33363	CG9226	WD repeat domain 79 homolog	VALIUM20
32926	CG17293	Wdr82	VALIUM20
25785	CG2759	white	VALIUM10
28980	CG2759	white	VALIUM10
33613	CG2759	white	VALIUM20
33623	CG2759	white	VALIUM20
33644	CG2759	white	VALIUM20
27319	CG5643	widerborst	VALIUM10
28939	CG5643	widerborst	VALIUM10
32469	CG4448	will decrease acetylation	VALIUM20
32952	CG17437	will die slowly	VALIUM20
33339	CG12340	windei	VALIUM20
29559	CG15288	wing blister	VALIUM10
32994	CG4889	wingless	VALIUM20
25949	CG10776	wishful thinking	VALIUM10
33635	CG12891	withered	VALIUM20
27057	CG5965	without children	VALIUM10
28947	CG8458	wnt inhibitor of Dorsal	VALIUM10
28892	CG1916	Wnt oncogene analog 2	VALIUM10
28534	CG6407	Wnt oncogene analog 5	VALIUM10
29441	CG1916	Wnt2	VALIUM10
29442	CG4698	Wnt4	VALIUM10
29443	CG6407	Wnt5	VALIUM10
29395	CG4969	Wnt6	VALIUM10
29560	CG8458	wntD	VALIUM10
29561	CG10382	wrapper	VALIUM10
32429	CG8804	wunen	VALIUM20
32430	CG8804	wunen	VALIUM20
32381	CG8805	wunen-2	VALIUM20
32423	CG8805	wunen-2	VALIUM20
25990	CG9415	X box binding protein-1	VALIUM10
29309	CG5675	X11L	VALIUM10
29444	CG4548	XNP	VALIUM10
32894	CG4548	XNP	VALIUM20
32986	CG1973	yata	VALIUM20
32953	CG17962	Z600	VALIUM20
26229	CG1046	zerknullt	VALIUM10
27674	CG10125	zero population growth	VALIUM10
29446	CG7803	zeste	VALIUM10
28960	CG3948	zetaCOP	VALIUM10
32954	CG6898	Zinc/iron regulated transporter-related protein 3	VALIUM20
29347	CG1322	Zn finger homeodomain 1	VALIUM10
29591	CG32018	Zyx102EF	VALIUM10
33430	CG17947	α Catenin	VALIUM20

32502	CG8308	α-Tubulin at 67C	VALIUM20

Supplemental Table 2. GO enrichment of identified genes.

Biological Process

Term	Background frequency	Sample frequency	Expected	+/-	P-value
regulation of cellular process (GO:0050794)	2829	27	6.85E+00	+	7.73E-12
regulation of biological process (GO:0050789)	3047	27	7.38E+00	+	5.10E-11
biological regulation (GO:0065007)	3307	27	8.01E+00	+	4.04E-10
regulation of gene expression (GO:0010468)	1115	17	2.70E+00	+	7.71E-09
regulation of metabolic process (GO:0019222)	1572	19	3.81E+00	+	1.77E-08
cell cycle (GO:0007049)	697	14	1.69E+00	+	1.96E-08
regulation of cellular metabolic process (GO:0031323)	1426	18	3.45E+00	+	3.56E-08
regulation of cellular macromolecule biosynthetic process (GO:2000112)	901	15	2.18E+00	+	4.79E-08
regulation of macromolecule biosynthetic process (GO:0010556)	903	15	2.19E+00	+	4.94E-08
cell cycle process (GO:0022402)	620	13	1.50E+00	+	6.26E-08
regulation of cellular biosynthetic process (GO:0031326)	953	15	2.31E+00	+	1.04E-07
regulation of biosynthetic process (GO:0009889)	954	15	2.31E+00	+	1.05E-07
regulation of macromolecule metabolic process (GO:0060255)	1322	17	3.20E+00	+	1.09E-07
positive regulation of biological process (GO:0048518)	1028	15	2.49E+00	+	2.93E-07
positive regulation of cellular process (GO:0048522)	917	14	2.22E+00	+	6.76E-07
regulation of primary metabolic process (GO:0080090)	1322	16	3.20E+00	+	1.02E-06
regulation of cell cycle process (GO:0010564)	192	8	4.65E-01	+	1.14E-06
negative regulation of cell cycle (GO:0045786)	126	7	3.05E-01	+	1.44E-06
regulation of mitotic cell cycle (GO:0007346)	213	8	5.16E-01	+	2.52E-06

compound eye photoreceptor cell differentiation (GO:0001751)	139	7	3.37E-01	+	2.81E-06

Molecular Function

Term	Background frequency	Sample frequency	Expected	+/-	P-value
protein binding (GO:0005515)	1725	19	4.18E+00	+	1.94E-08
binding (GO:0005488)	4722	25	1.14E+01	+	2.75E-05
ion binding (GO:0043167)	2265	14	5.49E+00	+	6.75E-03
translation initiation factor binding (GO:0031369)	15	2	3.63E-02	+	1.00E-02
nucleotide binding (GO:0000166)	1047	9	2.54E+00	+	1.05E-02
nucleoside phosphate binding (GO:1901265)	1047	9	2.54E+00	+	1.05E-02
small molecule binding (GO:0036094)	1130	9	2.74E+00	+	1.82E-02
heterocyclic compound binding (GO:1901363)	2504	14	6.07E+00	+	1.91E-02
organic cyclic compound binding (GO:0097159)	2520	14	6.10E+00	+	2.04E-02
transcription regulatory region DNA binding (GO:0044212)	105	3	2.54E-01	+	3.36E-02
purine ribonucleoside triphosphate binding (GO:0035639)	769	7	1.86E+00	+	3.39E-02
purine ribonucleotide binding (GO:0032555)	770	7	1.87E+00	+	3.42E-02
purine ribonucleoside binding (GO:0032550)	770	7	1.87E+00	+	3.42E-02
purine nucleoside binding (GO:0001883)	770	7	1.87E+00	+	3.42E-02
regulatory region nucleic acid binding (GO:0001067)	106	3	2.57E-01	+	3.45E-02
regulatory region DNA binding (GO:0000975)	106	3	2.57E-01	+	3.45E-02
ribonucleoside binding (GO:0032549)	774	7	1.88E+00	+	3.52E-02
purine nucleotide binding (GO:0017076)	775	7	1.88E+00	+	3.55E-02
nucleoside binding (GO:0001882)	776	7	1.88E+00	+	3.57E-02

Cellular Component

Term	Background frequency	Sample frequency	Expected	+/-	P-value
cell (GO:0005623)	5546	28	1.34E+01	+	4.63E-06
cell part (GO:0044464)	5546	28	1.34E+01	+	4.63E-06
intracellular part (GO:0044424)	4700	25	1.14E+01	+	3.11E-05
intracellular (GO:0005622)	4785	25	1.16E+01	+	4.53E-05
cytoskeleton (GO:0005856)	598	9	1.45E+00	+	1.78E-04
organelle (GO:0043226)	4001	22	9.69E+00	+	2.06E-04
microtubule cytoskeleton (GO:0015630)	482	8	1.17E+00	+	3.08E-04
nucleus (GO:0005634)	1955	15	4.74E+00	+	3.52E-04
intracellular non-membrane-bounded organelle (GO:0043232)	1466	13	3.55E+00	+	3.67E-04
non-membrane-bounded organelle (GO:0043228)	1466	13	3.55E+00	+	3.67E-04
intracellular organelle (GO:0043229)	3948	21	9.56E+00	+	7.53E-04
organelle part (GO:0044422)	2363	16	5.72E+00	+	7.72E-04
cytoskeletal part (GO:0044430)	555	8	1.34E+00	+	8.42E-04
FACT complex (GO:0035101)	4	2	9.69E-03	+	9.05E-04
nucleoplasm part (GO:0044451)	292	6	7.07E-01	+	1.31E-03
nucleoplasm (GO:0005654)	325	6	7.87E-01	+	2.35E-03
intracellular organelle part (GO:0044446)	2327	15	5.64E+00	+	2.82E-03
protein complex (GO:0043234)	2060	14	4.99E+00	+	3.04E-03
membrane-bounded organelle (GO:0043227)	3298	18	7.99E+00	+	3.50E-03

Supplemental Table 3. List of identified genes in cultured cells and tissues from previous publications, including S2 cells, wings, eyes, Kc 167 cells and external sensory organs.

S2 cells

CG#	Gene Name
CG10047	SytIV
CG10064	CG10064
CG10093	Cyp313a3
CG10103	CG10103

CG10107	CG10107
CG10122	RpI1
CG10130	Sec61beta
CG10161	eIF-3p66
CG10173	Best2
CG10221	CG10221
CG1024	CG1024
CG10261	aPKC
CG10286	CG10286
CG10289	CG10289
CG10377	Hrb27C
CG10415	TfIIEalpha
CG1044	dos
CG10444	CG10444
CG10447	CG10447
CG10484	Dox-A2
CG10498	cdc2c
CG10541	Tektin-C
CG1057	MED31
CG10583	Sse
CG1064	Snr1
CG10652	RpL30
CG10670	Gen
CG10674	CG10674
CG1070	Alh
CG10712	Chro
CG10713	CG10713
CG10719	brat
CG10754	CG10754
CG10772	Fur1
CG10789	ng4
CG10846	dyn-p25
CG10851	B52
CG10868	orb
CG10883	CG10883
CG10895	lok
CG10908	CG10908
CG10922	La
CG10948	CG10948
CG10954	Arc-p34
CG10955	Rtf1
CG1098	Madm

CG10988	l(1)dd4
CG1100	Rpn5
CG11006	CG11006
CG11027	Arf102F
CG11069	CG11069
CG1107	aux
CG11115	Ssl1
CG11125	CG11125
CG11132	DMAP1
CG1115	CG1115
CG11170	CG11170
CG11176	Tango2
CG11184	Upf3
CG11203	CG11203
CG11246	Rpb8
CG11266	CG11266
CG11299	CG11299
CG11310	Cpr78Ca
CG11390	PebIII
CG11491	br
CG11505	CG11505
CG11518	pygo
CG11522	RpL6
CG11555	CG11555
CG11579	arm
CG11582	CG11582
CG1161	CG1161
CG11621	Pi3K68D
CG1163	RpII18
CG11635	CG11635
CG11727	CG11727
CG11734	HERC2
CG11761	trsn
CG11781	CG11781
CG11844	CG11844
CG11847	Clbn
CG11858	CG11858
CG11861	gft
CG11873	CG11873
CG11876	CG11876
CG11888	Rpn2
CG11907	Ent1

CG11968	CG11968
CG11979	Rpb5
CG11982	CG11982
CG11984	CG11984
CG11985	CG11985
CG11989	Ard1
CG12019	Cdc37
CG12031	MED14
CG12050	CG12050
CG12052	lola
CG12069	CG12069
CG12113	l(1)G0095
CG12114	spn-F
CG12124	mxc
CG12131	Adam
CG12202	Nat1
CG12217	PpV
CG12218	mei-P26
CG12238	e(y)3
CG12254	MED25
CG12261	mRpS22
CG12267	CG12267
CG12297	BG4
CG12301	CG12301
CG12306	polo
CG12327	Best3
CG12359	Ulp1
CG12366	O-fut1
CG12370	CG12370
CG12402	CG12402
CG12404	CG12404
CG1242	Hsp83
CG12436	CG12436
CG12437	raw
CG1244	MEP-1
CG1245	MED27
CG1249	snRNP2
CG12499	CG12499
CG12530	Cdc42
CG1263	RpL8
CG12640	CG12640
CG12699	CG12699

CG12708	CG12708
CG12753	CG12753
CG12756	Eaf6
CG1276	TfIIEbeta
CG12768	CG12768
CG12770	Vps28
CG12775	RpL21
CG12921	mRpL42
CG12934	CG12934
CG12936	mms4
CG12944	Obp47a
CG13000	CG13000
CG13033	CG13033
CG13055	CG13055
CG13085	CG13085
CG13095	CG13095
CG13185	CG13185
CG13188	CG13188
CG13204	CG13204
CG1322	zfh1
CG13231	CG13231
CG13252	CG13252
CG13281	Cas
CG13296	CG13296
CG13298	CG13298
CG13312	CG13312
CG13329	cid
CG13348	Aats-phe
CG13387	emb
CG13425	bl
CG13438	CG13438
CG13531	CG13531
CG13550	CG13550
CG13560	CG13560
CG13566	CG13566
CG1358	CG1358
CG13594	CG13594
CG13628	Rpb10
CG13646	CG13646
CG13652	CG13652
CG13658	CG13658
CG13716	CG13716

CG13732	qjt
CG13737	CG13737
CG13739	CG13739
CG1378	tll
CG13803	DmsR-2
CG13867	MED8
CG13887	CG13887
CG13900	CG13900
CG13917	CG13917
CG13947	CG13947
CG1397	CG1397
CG14001	bchs
CG14007	CG14007
CG14030	Bub1
CG1406	U2A
CG14077	CG14077
CG14080	Mkp3
CG14168	CG14168
CG14180	CG14180
CG14189	CG14189
CG14210	CG14210
CG14213	CG14213
CG14216	CG14216
CG14230	CG14230
CG14299	CG14299
CG14305	CG14305
CG14309	CG14309
CG14310	CG14310
CG14312	CG14312
CG1433	Atu
CG14351	CG14351
CG14352	CG14352
CG14359	CG14359
CG14394	CG14394
CG14414	CG14414
CG14422	CG14422
CG14435	CG14435
CG14445	CG14445
CG14485	swi2
CG14507	CG14507
CG14536	Herp
CG14542	CG14542

CG14573	CG14573
CG14585	Ir75a
CG14635	CG14635
CG14637	abs
CG14641	CG14641
CG14704	PGRP-LB
CG14712	CG14712
CG14721	CG14721
CG1475	RpL13A
CG14750	Vps25
CG14780	CG14780
CG14782	CG14782
CG14869	CG14869
CG1490	Usp7
CG14957	CG14957
CG14998	CG14998
CG15015	Cip4
CG1503	CG1503
CG1504	CG1504
CG15042	CG15042
CG15046	CG15046
CG15088	CG15088
CG15097	CG15097
CG15098	CG15098
CG15117	CG15117
CG15127	CG15127
CG1515	Ykt6
CG15167	CG15167
CG15179	sunz
CG1519	Prosalpha7
CG15210	CG15210
CG15218	CycK
CG15224	CkIIbeta
CG15270	CG15270
CG1528	gammaCop
CG15282	CG15282
CG15319	nej
CG15321	CG15321
CG15323	CG15323
CG15415	Spindly
CG15439	CG15439
CG15442	RpL27A

CG1554	RpII215
CG1559	Upf1
CG15667	Sara
CG1584	Orc6
CG15888	CG15888
CG15923	CG15923
CG15929	lin-52
CG1632	CG1632
CG1646	CG1646
CG1657	CG1657
CG1664	sbr
CG1676	cactin
CG16766	TyrRII
CG16787	CG16787
CG16792	DebB
CG16817	CG16817
CG16890	CG16890
CG16903	CG16903
CG16916	Rpt3
CG1692	mal
CG16941	CG16941
CG16975	Sfmbt
CG16983	skpA
CG17052	obst-A
CG17077	pnt
CG17090	hipk
CG17119	CG17119
CG17209	CG17209
CG1721	Pglym78
CG17293	CG17293
CG17329	CG17329
CG17369	Vha55
CG17521	Qm
CG17577	Cyp9h1
CG17611	eIF6
CG1762	betaInt-nu
CG1765	EcR
CG17658	CG17658
CG17680	CG17680
CG17704	Nipped-B
CG1782	Uba1
CG17828	mod(r)

CG17836	CG17836
CG17855	CG17855
CG17870	14-3-3zeta
CG1789	CG1789
CG17958	Sry-delta
CG1800	pasha
CG18001	RpL38
CG18023	Eip78C
CG1821	RpL31
CG18287	ppk19
CG18292	CG18292
CG18332	CSN3
CG18398	Tango6
CG18426	ytr
CG18497	spen
CG18508	CG18508
CG1856	ttk
CG18591	CG18591
CG18622	CG18622
CG18778	Cpr65Au
CG18780	MED20
CG18801	Ku80
CG18803	Psn
CG1897	Dr
CG1906	alph
CG1908	CG1908
CG1910	CG1910
CG1937	sip3
CG1956	R
CG1965	CG1965
CG1970	CG1970
CG1972	CG1972
CG1981	Thd1
CG1982	Sodh-1
CG1983	CG1983
CG2013	UbcD6
CG2021	CG2021
CG2028	CkIalpha
CG2038	CSN7
CG2063	CG2063
CG2093	CG2093
CG2099	RpL35A

CG2107	CG2107
CG2128	Hdac3
CG2161	Rga
CG2163	Pabp2
CG2216	Fer1HCH
CG2246	CG2246
CG2249	CG2249
CG2252	fs(1)h
CG2253	Upf2
CG2261	CstF-50
CG2263	CG2263
CG2275	Jra
CG2446	CG2446
CG2448	FucT6
CG2662	CG2662
CG2701	CG2701
CG2746	RpL19
CG2807	CG2807
CG2819	Pph13
CG2845	phl
CG2848	Trn-SR
CG2899	ksr
CG2917	Orc4
CG2925	noi
CG2931	CG2931
CG2960	RpL40
CG3000	rap
CG30035	CG30035
CG30039	CG30039
CG30053	CG30053
CG30067	Obp50a
CG30072	Obp50c
CG30080	CG30080
CG3009	CG3009
CG30118	CG30118
CG3017	Alas
CG30181	CG30181
CG3019	su(w[a])
CG30325	CG30325
CG30354	CG30354
CG30389	CG30389
CG30391	CG30391

CG30410	CG30410
CG30420	Atf-2
CG30476	ave
CG3062	CG3062
CG31029	CG31029
CG3107	CG3107
CG31108	CG31108
CG31122	CG31122
CG31141	CG31141
CG31152	rumi
CG31155	Rpb7
CG31219	CG31219
CG31224	CG31224
CG31256	Brf
CG31258	Cenp-C
CG3127	PgK
CG31291	CG31291
CG31313	CG31313
CG31335	Gr93d
CG31371	CG31371
CG31390	MED7
CG31410	CG31410
CG31482	CG31482
CG31546	CG31546
CG31605	Bsg
CG31632	sens-2
CG31634	Oatp26F
CG31651	pgant5
CG3169	Spt3
CG31709	CG31709
CG31713	Apf
CG31755	CG31755
CG3180	RpII140
CG3181	Ts
CG31819	CG31819
CG3183	geminin
CG31848	CG31848
CG3186	eIF-5A
CG3189	Dpit47
CG31894	CG31894
CG31908	CG31908
CG31936	Gr22e

CG32000	CG32000
CG3203	RpL17
CG32067	simj
CG32219	CG32219
CG32235	CG32235
CG3224	CG3224
CG32249	CG32249
CG32372	CG32372
CG32382	sphinx2
CG32393	dikar
CG32401	Or65a
CG32409	CG32409
CG3248	Cog3
CG32498	dnc
CG32534	CG32534
CG3254	pgant2
CG32595	CG32595
CG32613	CG32613
CG32721	NELF-B
CG3278	Tif-IA
CG3280	CG3280
CG32816	CG32816
CG3284	RpII15
CG3303	CG3303
CG33051	CG33051
CG33090	CG33090
CG33106	mask
CG33109	CG33109
CG33133	grau
CG3314	RpL7A
CG33159	CG33159
CG33191	CG33191
CG3320	Rab1
CG33217	CG33217
CG33220	CG33220
CG33261	Trl
CG33288	CG33288
CG33303	CG33303
CG33321	CheB38b
CG33482	CG33482
CG33512	dpr4
CG33516	dpr3

CG33526	PNUTS
CG33554	Nipped-A
CG3363	CG3363
CG33697	CG33697
CG33757	CG33757
CG3379	His4r
CG3388	gsb
CG33919	CG33919
CG33956	kay
CG33980	Vsx2
CG33988	CG33988
CG3412	slmb
CG34123	CG34123
CG34124	CG34124
CG34131	CG34131
CG34157	Dys
CG3423	SA
CG3424	path
CG34341	Pde11
CG34345	CG34345
CG34407	Not1
CG34416	Ank2
CG34422	CG34422
CG3497	Su(H)
CG3539	Slh
CG3546	CG3546
CG3582	U2af38
CG3585	CG3585
CG3605	CG3605
CG3606	caz
CG3634	CG3634
CG3644	bic
CG3653	kirre
CG3661	RpL23
CG3664	Rab5
CG3689	CG3689
CG3696	kis
CG3725	Ca-P60A
CG3738	Cks30A
CG3773	CG3773
CG3780	Spx
CG3832	Phm

CG3857	CG3857
CG3911	Bet3
CG3924	Chi
CG3936	N
CG3949	hoip
CG3971	Baldspot
CG3992	srp
CG3997	RpL39
CG3998	zf30C
CG3999	CG3999
CG4003	pont
CG4005	yki
CG4033	RpI135
CG4070	Tis11
CG4079	Taf11
CG4108	Chmp1
CG4111	RpL35
CG4145	Cg25C
CG4214	Syx5
CG42277	rn
CG42403	Ca-beta
CG4244	Su(dx)
CG4247	mRpS10
CG42551	larp
CG4294	CG4294
CG4320	raptor
CG4360	CG4360
CG4364	CG4364
CG4371	GstD7
CG4374	CG4374
CG4394	Traf-like
CG4400	CG4400
CG4435	FucTB
CG4465	CG4465
CG4472	Idgf1
CG4528	snf
CG4570	CG4570
CG4585	CG4585
CG4602	Srp54
CG4604	GLaz
CG4611	CG4611
CG4636	SCAR

CG4651	RpL13
CG4710	Pino
CG4744	CG4744
CG4752	CG4752
CG4760	bol
CG4769	CG4769
CG4785	CG4785
CG4792	Dcr-1
CG4816	qkr54B
CG4817	Ssrp
CG4825	CG4825
CG4838	beat-Ic
CG4849	CG4849
CG4859	Mmp1
CG4863	RpL3
CG4875	CG4875
CG4897	RpL7
CG4912	eEF1delta
CG4936	CG4936
CG4954	eIF3-S8
CG4993	PRL-1
CG5003	CG5003
CG5010	CG5010
CG5014	Vap-33-1
CG5092	Tor
CG5099	msi
CG5147	CG5147
CG5163	TfIIA-S
CG5166	Atx2
CG5179	Cdk9
CG5183	KdelR
CG5208	Patr-1
CG5222	CG5222
CG5224	CG5224
CG5229	chm
CG5277	Ip259
CG5290	CG5290
CG5333	trus
CG5345	Eip55E
CG5352	SmB
CG5355	CG5355
CG5422	Rox8

CG5429	Atg6
CG5440	CG5440
CG5442	SC35
CG5444	Taf4
CG5451	CG5451
CG5460	H
CG5482	CG5482
CG5489	Atg7
CG5505	Usp36
CG5510	CG5510
CG5514	CG5514
CG5543	CG5543
CG5596	Mlc1
CG5597	CG5597
CG5625	CG5625
CG5649	kin17
CG5654	yps
CG5660	CG5660
CG5680	bsk
CG5684	Pop2
CG5691	CG5691
CG5694	CG5694
CG5748	Hsf
CG5785	thr
CG5805	CG5805
CG5824	l(3)07882
CG5827	RpL37A
CG5836	SF1
CG5837	Hem
CG5844	CG5844
CG5864	AP-1sigma
CG5874	NELF-A
CG5915	Rab7
CG5927	Her
CG5933	CG5933
CG5941	CG5941
CG5945	CG5945
CG5949	DNApol-delta
CG5954	l(3)mbt
CG5994	Nelf-E
CG6006	CG6006

CG6033	drk
CG6052	CG6052
CG6055	CG6055
CG6092	Dak1
CG6116	CG6116
CG6121	Tip60
CG6127	Ser
CG6134	spz
CG6141	RpL9
CG6143	Pep
CG6173	kal-1
CG6202	Surf4
CG6203	Fmr1
CG6214	MRP
CG6222	su(s)
CG6233	Ufd1-like
CG6234	CG6234
CG6259	CG6259
CG6272	CG6272
CG6290	CG6290
CG6292	CycT
CG6297	JIL-1
CG6300	CG6300
CG6341	Ef1beta
CG6369	Smg6
CG6370	CG6370
CG6386	ball
CG6409	CG6409
CG6479	CG6479
CG6481	CG6481
CG6493	Dcr-2
CG6502	E(z)
CG6510	RpL18A
CG6538	TfIIFbeta
CG6546	Bap55
CG6554	Art1
CG6556	cnk
CG6568	CG6568
CG6588	Fas1
CG6592	CG6592
CG6593	Pp1alpha-96A

CG6610	CG6610
CG6632	Ing3
CG6637	lsn
CG6671	AGO1
CG6686	CG6686
CG6703	Caki
CG6708	Osbp
CG6711	Taf2
CG6712	CG6712
CG6729	CG6729
CG6757	SH3PX1
CG6766	CG6766
CG6767	CG6767
CG6770	CG6770
CG6790	CG6790
CG6814	Mat89Bb
CG6817	foi
CG6840	Rpb11
CG6842	CG6842
CG6846	RpL26
CG6866	loqs
CG6873	CG6873
CG6884	MED11
CG6995	CG6995
CG7001	Pk17E
CG7007	VhaPPA1-1
CG7012	nct
CG7015	Unr
CG7028	CG7028
CG7031	CG7031
CG7055	dalao
CG7070	PyK
CG7074	mio
CG7085	l(2)s5379
CG7088	bnb
CG7099	CG7099
CG7100	CadN
CG7109	mts
CG7111	Rack1
CG7147	kuz
CG7168	CG7168

CG7177	CG7177
CG7185	CG7185
CG7187	Ssdp
CG7196	CG7196
CG7200	CG7200
CG7220	CG7220
CG7235	Hsp60C
CG7275	CG7275
CG7338	CG7338
CG7339	CG7339
CG7375	CG7375
CG7379	CG7379
CG7380	baf
CG7421	Nopp140
CG7424	RpL36A
CG7425	eff
CG7434	RpL22
CG7437	mub
CG7490	RpLP0
CG7494	mRpL1
CG7499	Rh50
CG7546	CG7546
CG7558	Arp66B
CG7578	sec71
CG7597	CG7597
CG7614	Mat1
CG7622	RpL36
CG7644	beat-Ib
CG7654	Tom20
CG7686	CG7686
CG7693	fray
CG7697	CstF-64
CG7704	Taf5
CG7726	RpL11
CG7738	CG7738
CG7740	prominin-like
CG7757	CG7757
CG7764	Tfb2
CG7773	fidipidine
CG7776	E(Pc)
CG7793	Sos

CG7802	CG7802
CG7843	CG7843
CG7850	puc
CG7879	CG7879
CG7885	RpII33
CG7891	Gie
CG7935	msk
CG7939	RpL32
CG7942	ldbr
CG7946	CG7946
CG7957	MED17
CG7962	CdsA
CG7974	CG7974
CG7977	RpL23A
CG7987	CG7987
CG7990	CG7990
CG7993	CG7993
CG8013	Su(z)12
CG8014	Rme-8
CG8019	hay
CG8048	Vha44
CG8053	eIF-1A
CG8055	shrb
CG8085	RN-tre
CG8103	Mi-2
CG8107	CalpB
CG8111	CG8111
CG8116	CG8116
CG8118	mam
CG8121	CG8121
CG8134	CG8134
CG8147	CG8147
CG8151	Tfb1
CG8152	CG8152
CG8170	CG8170
CG8186	Vha36
CG8200	Flo
CG8207	CG8207
CG8208	MBD-like
CG8210	Vha14
CG8233	CG8233
CG8237	CG8237

CG8248	CG8248
CG8264	Bx42
CG8274	Mtor
CG8276	bin3
CG8290	CG8290
CG8309	Tango7
CG8315	CG8315
CG8323	CG8323
CG8325	l(2)k14710
CG8344	RpIII128
CG8358	CG8358
CG8363	Papss
CG8369	CG8369
CG8370	CG8370
CG8376	ap
CG8392	l(2)05070
CG8426	l(2)NC136
CG8427	SmD3
CG8434	lbk
CG8444	CG8444
CG8454	Vps16A
CG8465	l(1)G0222
CG8491	kto
CG8500	CG8500
CG8532	lqf
CG8568	CG8568
CG8580	akirin
CG8599	Su(var)3-7
CG8612	mRpL50
CG8613	CG8613
CG8615	RpL18
CG8651	trx
CG8694	LvpD
CG8705	pnut
CG8712	CG8712
CG8717	slv
CG8722	Nup44A
CG8815	Sin3A
CG8818	CG8818
CG8858	CG8858
CG8863	Droj2
CG8882	Trip1

CG8885	CG8885
CG8886	l(2)05714
CG8887	ash1
CG8890	Gmd
CG8946	Sply
CG8949	CG8949
CG8952	CG8952
CG8954	Smg5
CG8963	CG8963
CG8979	CG8979
CG8980	NiPp1
CG9012	Chc
CG9022	Ost48
CG9033	Tsp47F
CG9077	Cpr47Ec
CG9091	RpL37a
CG9121	CG9121
CG9135	CG9135
CG9166	312
CG9193	mus209
CG9195	Scamp
CG9215	CG9215
CG9222	CG9222
CG9224	sog
CG9253	CG9253
CG9282	RpL24
CG9293	CG9293
CG9300	CG9300
CG9305	CG9305
CG9311	CG9311
CG9324	Pomp
CG9383	asf1
CG9386	CG9386
CG9423	Kap-alpha3
CG9441	Pu
CG9446	coro
CG9452	CG9452
CG9548	CG9548
CG9554	eya
CG9578	CG9578
CG9615	tex
CG9623	if

CG9648	Max
CG9676	CG9676
CG9696	dom
CG9712	TSG101
CG9748	bel
CG9749	Abi
CG9750	rept
CG9761	Nep2
CG9779	CG9779
CG9802	Cap
CG9888	Fib
CG9922	CG9922
CG9932	CG9932
CG9936	skd
CG9983	Hrb98DE
CG9984	TH1
CG9986	CG9986
CG16901	sqd

Wings

CG#	Gene Name
CG10289	CG10289
CG1064	Snr1
CG10712	Chro
CG10754	CG10754
CG10846	dyn-p25
CG10948	CG10948
CG10954	Arc-p34
CG11006	CG11006
CG11115	Ssl1
CG11876	CG11876
CG11984	CG11984
CG12019	Cdc37
CG12202	Nat1
CG1242	Hsp83
CG12437	raw
CG12499	CG12499
CG12770	Vps28
CG12921	mRpL42

CG13329	cid
CG14080	Mkp3
CG14394	CG14394
CG14750	Vps25
CG15218	CycK
CG1528	gammaCop
CG15282	CG15282
CG1657	CG1657
CG17293	CG17293
CG18497	spen
CG1972	CG1972
CG2013	UbcD6
CG2261	CstF-50
CG2845	phl
CG30476	ave
CG3127	Pgk
CG33261	Trl
CG33554	Nipped-A
CG3857	CG3857
CG3911	Bet3
CG4611	CG4611
CG4817	Ssrp
CG4875	CG4875
CG5099	msi
CG5440	CG5440
CG5460	H
CG5844	CG5844
CG6369	Smg6
CG6637	lsn
CG7070	PyK
CG7338	CG7338
CG7339	CG7339
CG7375	CG7375
CG7379	CG7379
CG7494	mRpL1
CG7654	Tom20
CG7776	E(Pc)
CG7843	CG7843
CG8085	RN-tre
CG8186	Vha36

CG8309	Tango7
CG8722	Nup44A
CG8885	CG8885
CG8946	Sply
CG9022	Ost48
CG9423	Kap-alpha3
CG9748	bel
CG9986	CG9986
CG10122	RpI1
CG1024	CG1024
CG10415	TfIIEalpha
CG10444	CG10444
CG10484	Dox-A2
CG10541	Tektin-C
CG1057	MED31
CG10583	Sse
CG1098	Madm
CG1115	CG1115
CG11246	Rpb8
CG11505	CG11505
CG11518	pygo
CG11761	trsn
CG11858	CG11858
CG12050	CG12050
CG12254	MED25
CG12267	CG12267
CG12306	polo
CG12404	CG12404
CG13185	CG13185
CG13281	Cas
CG1358	CG1358
CG13917	CG13917
CG14213	CG14213
CG14435	CG14435
CG16983	skpA
CG17209	CG17209
CG18780	MED20
CG18801	Ku80
CG18803	Psn
CG2107	CG2107

CG2128	Hdac3
CG2161	Rga
CG2163	Pabp2
CG2275	Jra
CG2925	noi
CG31155	Rpb7
CG31256	Brf
CG31390	MED7
CG31634	Oatp26F
CG3180	RpII140
CG3181	Ts
CG31908	CG31908
CG3248	Cog3
CG3278	Tif-IA
CG3284	RpII15
CG33051	CG33051
CG33106	mask
CG33217	CG33217
CG33956	kay
CG3423	SA
CG34407	Not1
CG3497	Su(H)
CG3539	Slh
CG3773	CG3773
CG3936	N
CG4033	RpI135
CG4294	CG4294
CG4320	raptor
CG4364	CG4364
CG4400	CG4400
CG4912	eEF1delta
CG5179	Cdk9
CG5277	Ip259
CG5422	Rox8
CG5684	Pop2
CG5785	thr
CG5941	CG5941
CG6127	Ser
CG6234	CG6234
CG6502	E(z)

CG6593	Pp1alpha-96A
CG6712	CG6712
CG6767	CG6767
CG6817	foi
CG6840	Rpb11
CG6995	CG6995
CG7012	Nct
CG7031	CG7031
CG7109	mts
CG7147	Kuz
CG7177	CG7177
CG7421	Nopp140
CG7597	CG7597
CG7614	Mat1
CG7693	fray
CG7740	prominin-like
CG7764	Tfb2
CG7885	RpII33
CG7946	CG7946
CG7962	CdsA
CG8013	Su(z)12
CG8014	Rme-8
CG8019	hay
CG8118	Mam
CG8200	Flo
CG8264	Bx42
CG8274	Mtor
CG8276	bin3
CG8344	RpIII128
CG8376	ap
CG8465	l(1)G0222
CG8887	ash1
CG8890	Gmd
CG8954	Smg5
CG9033	Tsp47F
CG9193	mus209
CG9441	Pu
CG9696	dom
CG9932	CG9932

CG9936	skd
CG10261	aPKC
CG1044	dos
CG10908	CG10908
CG10955	Rtf1
CG11184	Upf3
CG11555	CG11555
CG11734	HERC2
CG11968	CG11968
CG12756	Eaf6
CG13550	CG13550
CG13867	MED8
CG1406	U2A
CG14210	CG14210
CG14230	CG14230
CG14351	CG14351
CG14712	CG14712
CG15015	Cip4
CG16903	CG16903
CG16975	Sfmbt
CG1721	Pglym78
CG18398	Tango6
CG1956	R
CG1965	CG1965
CG2028	CkIalpha
CG2216	Fer1HCH
CG2662	CG2662
CG2848	Trn-SR
CG3000	rap
CG31651	pgant5
CG3224	CG3224
CG32721	NELF-B
CG33303	CG33303
CG33526	PNUTS
CG3634	CG3634
CG3644	bic
CG3696	kis
CG3725	Ca-P60A
CG3971	Baldspot
CG3992	srp

CG3998	zf30C
CG4005	yki
CG4244	Su(dx)
CG4374	CG4374
CG4838	beat-Ic
CG4936	CG4936
CG5014	Vap-33-1
CG5166	Atx2
CG5208	Patr-1
CG5222	CG5222
CG5543	CG5543
CG5864	AP-1sigma
CG6092	Dak1
CG6121	Tip60
CG6233	Ufd1-like
CG6259	CG6259
CG6292	CycT
CG6341	Ef1beta
CG6386	ball
CG6538	TfIIFbeta
CG7001	Pk17E
CG7007	VhaPPA1-1
CG7055	dalao
CG7085	l(2)s5379
CG7099	CG7099
CG7185	CG7185
CG7275	CG7275
CG7380	baf
CG7578	sec71
CG7935	msk
CG7957	MED17
CG7993	CG7993
CG8048	Vha44
CG8233	Rcd1
CG8454	Vps16A
CG8491	kto
CG8599	Su(var)3-7
CG8651	trx
CG8815	Sin3A
CG8863	Droj2

CG8886	l(2)05714
CG9135	CG9135
CG9300	CG9300
CG9305	CG9305
CG9311	mop
CG9548	CG9548
CG9712	TSG101
CG9749	Abi
CG9779	CG9779
CG9802	Cap
CG9922	CG9922

Eyes

CG#	Gene Name
CG1024	CG1024
CG10719	brat
CG1115	CG1115
CG11184	Upf3
CG12113	l(1)G0095
CG13917	CG13917
CG14641	CG14641
CG17119	CG17119
CG17209	CG17209
CG2253	Upf2
CG2263	CG2263
CG3181	Ts
CG3278	Tif-IA
CG33106	mask
CG33303	CG33303
CG33526	PNUTS
CG3423	SA
CG3539	Slh
CG3696	kis
CG3857	CG3857
CG4003	pont
CG4817	Ssrp
CG5514	CG5514
CG7578	sec71

CG7597	CG7597
CG8344	RpIII128
CG8532	lqf
CG8963	CG8963
CG9022	Ost48
CG9305	CG9305
CG9750	rept
CG9802	Cap
CG10103	CG10103
CG10221	CG10221
CG10289	CG10289
CG1044	dos
CG10754	CG10754
CG10868	orb
CG11505	CG11505
CG11621	Pi3K68D
CG11876	CG11876
CG11907	Ent1
CG11984	CG11984
CG12217	PpV
CG12261	mRpS22
CG1242	Hsp83
CG12770	Vps28
CG13296	CG13296
CG13298	CG13298
CG13550	CG13550
CG13803	DmsR-2
CG1406	U2A
CG14080	Mkp3
CG14213	CG14213
CG14351	CG14351
CG14507	CG14507
CG14542	CG14542
CG14750	Vps25
CG1490	Usp7
CG15210	CG15210
CG1657	CG1657
CG16903	CG16903
CG17369	Vha55
CG17836	CG17836

CG17855	CG17855
CG17870	14-3-3zeta
CG1965	CG1965
CG1981	Thd1
CG2013	UbcD6
CG2093	Vps13
CG2848	Trn-SR
CG2925	noi
CG30035	CG30035
CG30420	Atf-2
CG31605	Bsg
CG31651	pgant5
CG31709	CG31709
CG33956	kay
CG3644	bic
CG3780	Spx
CG3998	zf30C
CG4108	Chmp1
CG4244	Su(dx)
CG4611	CG4611
CG4636	SCAR
CG4825	CG4825
CG4838	beat-Ic
CG4849	CG4849
CG4912	eEF1delta
CG5099	msi
CG5166	Atx2
CG5179	Cdk9
CG5183	KdelR
CG5422	Rox8
CG5844	CG5844
CG5954	l(3)mbt
CG6006	CG6006
CG6055	CG6055
CG6092	Dak1
CG6121	Tip60
CG6214	MRP
CG6233	Ufd1-like
CG6259	CG6259
CG6632	Ing3

CG6637	lsn
CG6686	CG6686
CG6767	CG6767
CG6842	Vps4
CG6873	CG6873
CG7111	Rack1
CG7185	CG7185
CG7425	eff
CG7494	mRpL1
CG7654	Tom20
CG7879	CG7879
CG7962	CdsA
CG8014	Rme-8
CG8186	Vha36
CG8210	Vha14
CG8233	Rcd1
CG8264	Bx42
CG8376	ap
CG8454	Vps16A
CG8863	Droj2
CG8887	ash1
CG9135	CG9135
CG9311	mop
CG9441	Pu
CG9548	CG9548
CG9696	dom
CG9712	TSG101
CG9936	skd
CG9984	TH1
CG9986	CG9986
CG1004	rho
CG10691	l(2)37Cc
CG10711	Vps36
CG10986	g
CG11173	usnp
CG11556	Rph
CG1193	CG1193
CG12230	car
CG1241	Atg2
CG14224	Ubqn

CG14899	CG14899
CG16901	sqd
CG16944	sesB
CG17645	Pglym87
CG18102	shi
CG18412	ph-p
CG1903	sno
CG2331	TER94
CG3093	dor
CG32350	CG32350
CG32632	Tango13
CG32677	X11Lbeta
CG32717	sdt
CG3326	CG3326
CG34411	CG34411
CG3473	CG3473
CG3595	sqh
CG3766	scat
CG4071	Vps20
CG4311	Hmgs
CG4562	CG4562
CG4701	CG4701
CG4713	l(2)gd1
CG5081	Syx7
CG5661	Sema-5c
CG5771	Rab11
CG5798	CG5798
CG5988	upd2
CG6692	Cp1
CG8251	Pgi
CG8428	spin

Kc 167 cells

CG#	Gene Name
CG10039	CG10039
CG10083	CG10083
CG10109	L
CG10122	RpI1

CG10200	CG10200
CG10236	LanA
CG10289	CG10289
CG10333	CG10333
CG10344	CG10344
CG10354	Rat1
CG10370	Tbp-1
CG10417	CG10417
CG10418	CG10418
CG10436	Pbprp1
CG1044	dos
CG10467	CG10467
CG10540	CG10540
CG10572	Cdk8
CG10578	DnaJ-1
CG10616	CG10616
CG1065	Scsalpha
CG10663	CG10663
CG10682	vih
CG10704	toe
CG10713	CG10713
CG1074	CG1074
CG10880	CG10880
CG10895	lok
CG10899	CG10899
CG10923	Klp67A
CG10960	CG10960
CG10966	rdgA
CG10967	Atg1
CG11006	CG11006
CG11010	Ent3
CG11027	Arf102F
CG11063	CG11063
CG11066	scarface
CG11084	pk
CG11095	CG11095
CG11107	CG11107
CG11132	DMAP1
CG11149	CG11149
CG11156	mus101

CG11158	CG11158
CG11164	CG11164
CG11172	NFAT
CG11245	CG11245
CG11278	Syx13
CG11289	CG11289
CG11329	CG11329
CG11347	CG11347
CG11375	polybromo
CG11397	glu
CG11488	mRpL10
CG11522	RpL6
CG11560	CG11560
CG11575	CG11575
CG11639	TfIIA-S-2
CG11790	CG11790
CG11856	Nup358
CG11929	CG11929
CG12021	Patj
CG12086	cue
CG12099	CG12099
CG12142	Tsp42Eg
CG12165	Incenp
CG12287	pdm2
CG12330	CG12330
CG12357	Cbp20
CG12467	CG12467
CG12505	CG12505
CG12543	CG12543
CG12576	CG12576
CG12841	Tsp42Ek
CG13054	CG13054
CG13073	CG13073
CG13281	Cas
CG13310	CG13310
CG13320	Sans
CG13323	CG13323
CG13344	CG13344
CG13345	tum
CG13360	CG13360

CG13387	emb
CG13504	CG13504
CG13544	CG13544
CG13590	CG13590
CG13624	CG13624
CG13716	CG13716
CG13726	Or74a
CG13773	CG13773
CG13783	CG13783
CG13802	CG13802
CG13810	CG13810
CG13879	CG13879
CG14006	CG14006
CG14073	CG14073
CG14206	RpS10b
CG14211	CG14211
CG14219	CG14219
CG14220	CG14220
CG14310	CG14310
CG14464	CG14464
CG14542	CG14542
CG14591	CG14591
CG14593	CG14593
CG14619	CG14619
CG1483	Map205
CG14830	CG14830
CG14880	CG14880
CG14911	CG14911
CG14938	crol
CG14972	CG14972
CG15013	dyl
CG15105	CG15105
CG15134	CG15134
CG15153	CG15153
CG15281	CG15281
CG15459	CG15459
CG15468	SIP3
CG15471	CG15471
CG15535	CG15535
CG1559	Upf1

CG15730	CG15730
CG15784	CG15784
CG15863, CG12130	CG15863, CG12130
CG15927	CG15927
CG16727	CG16727
CG16778	Tkr
CG16786	CG16786
CG16801	CG16801
CG1683	Ant2
CG16903	CG16903
CG16941	CG16941
CG17002	CG17002
CG1709	Vha100-1
CG1710	Hcf
CG17177	CG17177
CG17189	CG17189
CG17213	Gr33a
CG17275	CG17275
CG17285	Fbp1
CG17329	CG17329
CG17332	VhaSFD
CG1746	CG1746
CG17562	CG17562
CG17665	CG17665
CG17746	CG17746
CG17894	cnc
CG17912	CG17912
CG1793	MED26
CG18009	Trf2
CG1814	CG1814
CG18176	defl
CG18208	CG18208
CG1822	bif
CG18262	CG18262
CG18304	CG18304
CG18319	ben
CG18343	CG18343
CG18350	Sxl
CG1856	ttk

CG1865	Spn43Ab
CG1874	CG1874
CG18740	mor
CG1972	CG1972
CG2009	bip2
CG2028	CkIalpha
CG2038	CSN7
CG2063	CG2063
CG2139	aralar1
CG2144	CG2144
CG2165	CG2165
CG2253	Upf2
CG2727	emp
CG2746	RpL19
CG2818	CG2818
CG2861	CG2861
CG2910	nito
CG30040	jeb
CG30170	bgcn
CG30181	CG30181
CG30182	CG30182
CG3019	su(wa)
CG30330	Gr59d
CG30365	CG30365
CG30404	Tango11
CG30418	nord
CG30456	CG30456
CG3083	Prx6005
CG31000	heph
CG31019	CG31019
CG31031	CG31031
CG31052	Tango12
CG31056	Acp98AB
CG31077	CG31077
CG31132	BRWD3
CG31150	CG31150
CG31196	14-3-3epsilon
CG3125	l(1)G0060
CG31257	CG31257

CG31275	CG31275
CG3136	CG3136
CG31404	CG31404
CG31419	CG31419
CG31488	CG31488
CG31499	CG31499
CG31519	Or82a
CG3159	Eaat2
CG31651	pgant5
CG31660	CG31660
CG31666	CG31666
CG31699	CG31699
CG3173	CG3173
CG31737	CG31737
CG31741	CG31741
CG3186	eIF-5A
CG31917	CG31917
CG31991	mdy
CG31992	CG31992
CG32030	CG32030
CG32119	CG32119
CG32258	Gr64e
CG32271	CG32271
CG32394	CG32394
CG32401	Or65a
CG32406	CG32406
CG32467	CG32467
CG32479	CG32479
CG32484	Sk2
CG32680	CG32680
CG3274	Bap170
CG32743	Smg1
CG32782	tlk
CG32824	CG32824
CG32987	CG32987
CG33104	eca
CG33116	CG33116
CG3332	CG3332
CG33458	CG33458
CG33466	Fs

CG33518	mun
CG3352	ft
CG33531	Ddr
CG33554	Nipped-A
CG3363	CG3363
CG33692	CG33692
CG33696	CG33696
CG3395	RpS9
CG33956	kay
CG33988	CG33988
CG34099	Mkp
CG3433	Coprox
CG3443	pcx
CG3450	ubl
CG3455	Rpt4
CG3481	Adh
CG3497	Su(H)
CG3595	sqh
CG3665	Fas2
CG3702	CG3702
CG3715	Shc
CG3830	vg
CG3920	l(2)k16918
CG3922	RpS17
CG3935	al
CG3954	csw
CG40006	CG40006
CG40175	CG40175
CG40235	CG40235
CG41056	CG41056
CG4168	CG4168
CG4192	kek3
CG4244	Su(dx)
CG4285	CG4285
CG4439	CG4439
CG4445	pgant3
CG4455	CG4455
CG4538	CG4538
CG4554	CG4554
CG4576	CG4576

CG4670	CG4670
CG4768	CG4768
CG4799	Pen
CG4812	Ser8
CG4817	Ssrp
CG4842	CG4842
CG4893	CG4893
CG5107	CG5107
CG5179	Cdk9
CG5183	KdelR
CG5206	bon
CG5224	CG5224
CG5320	Gdh
CG5412	CG5412
CG5441	dei
CG5460	H
CG5562	gbb
CG5581	Ote
CG5591	CG5591
CG5642	CG5642
CG5706	CG5706
CG5720	CG5720
CG5813	chif
CG5825	His3.3A
CG5885	CG5885
CG6038	CG6038
CG6115	CG6115
CG6121	Tip60
CG6147	Tsc1
CG6196	CG6196
CG6315	fl(2)d
CG6341	Ef1beta
CG6386	ball
CG6463	CG6463
CG6527	CG6527
CG6546	Bap55
CG6580	Jon65Aii
CG6604	H15
CG6620	ial
CG6641	Pbprp5

CG6729	CG6729
CG6794	Dif
CG6852	CG6852
CG6899	Ptp4E
CG6931	CG6931
CG6945	CG6945
CG7066	CG7066
CG7085	l(2)s5379
CG7099	CG7099
CG7212	cdm
CG7250	Toll-6
CG7263	CG7263
CG7274	CG7274
CG7427	CG7427
CG7471	Rpd3
CG7483	eIF4AIII
CG7525	Tie
CG7530	CG7530
CG7643	ald
CG7698	CG7698
CG7702	CG7702
CG7707	CG7707
CG7708	CG7708
CG7713	CG7713
CG7741	CG7741
CG7776	E(Pc)
CG7793	Sos
CG7825	Rad17
CG7831	ncd
CG7957	MED17
CG8008	CG8008
CG8029	CG8029
CG8092	CG8092
CG8189	ATPsyn-b
CG8222	Pvr
CG8276	bin3
CG8399	CG8399
CG8407	CG8407
CG8426	l(2)NC136
CG8539	CG8539

CG8552	CG8552
CG8562	CG8562
CG8567	Deaf1
CG8601	mus312
CG8740	CG8740
CG8815	Sin3A
CG8834	CG8834
CG8849	mRpL24
CG8882	Trip1
CG8884	Sap47
CG8975	RnrS
CG8983	ERp60
CG8989	His3.3B
CG9045	Myb
CG9083	CG9083
CG9088	lid
CG9094	CG9094
CG9120	LysX
CG9124	eIF-3p40
CG9177	eIF5
CG9287	CG9287
CG9293	CG9293
CG9317	CG9317
CG9481	Ugt37b1
CG9527	CG9527
CG9556	alien
CG9559	fog
CG9668	Rh4
CG9669	CG9669
CG9774	rok
CG9775	CG9775
CG9882	Art7
CG9954	maf-S
CR31400	Hsromega
CR32864	7SLRNA

External sensory organ

CG#	Gene Name

CG3796	ac
CG4260	alpha-adaptin
CG2075	Aly
CG32707	APC4
CG2855	aph-1
CG10954	Arc-p34
CG4531	argos
CG9901	Arp14D
CG8322	ATPCL
CG11154	ATPsyn-beta
CG4412	ATPsyn-Cf6
CG4692	ATPsynF
CG5055	baz
CG3401	betaTub60D
CG4722	bib
CG6897	bora
CG10542	Bre1
CG6703	caki
CG8472	Cam
CG18408	CAP
CG13281	Cas
CG5363	cdc2
CG10375	CG10375
CG11597	CG11597
CG12007	CG12007
CG12496	CG12496
CG13369	CG13369
CG13623	CG13623
CG14407	CG14407
CG14544	CG14544
CG15769	CG15769
CG2091	CG2091
CG34401	CG34401
CG3689	CG3689
CG41105	CG41105
CG4538	CG4538
CG4733	CG4733
CG5608	CG5608
CG5640	CG5640

CG5919	CG5919
CG6255	CG6255
CG6509	CG6509
CG6512	CG6512
CG6830	CG6830
CG7714	CG7714
CG8142	CG8142
CG8176	CG8176
CG8239	CG8239
CG9596	CG9596
CG42741	CG9895
CG11798	chn
CG31012	cindr
CG42312	cno
CG3889	CSN1b
CG18332	CSN3
CG8725	CSN4
CG6932	CSN6
CG2038	CSN7
CG42522	CSN8
CG7583	CtBP
CG6998	ctp
CG5940	CycA
CG9964	Cyp309a1
CG4952	dac
CG5441	dei
CG12265	Det
CG15367	Dip1
CG3619	Dl
CG1725	dlg1
CG32315	dlt
CG33196	dp
CG3929	dx
CG33104	eca
CG12676	ed
CG7883	elF2B-alpha
CG10315	elF2B-delta
CG1007	emc
CG7923	Fad2
CG6315	fl(2)d

CG3874	frc
CG17697	fz
CG6975	gig
CG1119	Gnf1
CG2204	G-oalpha47A
CG5820	Gp150
CG8384	gro
CG5460	H
CG8094	Hex-c
CG4311	Hmgs
CG10377	Hrb27c
CG5695	jar
	Jhl-1
CG9423	Kap-alpha3
CG4713	l(2)gd1
CG6944	Lam
CG4088	lat
CG6098	Lrr47
CG8118	mam
CG7614	Mat1
CG4143	mbf1
CG18582	mbt
CG7538	Mcm2
CG4039	Mcm6
CG5362	Mdh1
CG17183	MED30
CG9473	MED6
CG5841	mib1
CG1216	mri
CG5099	msi
CG7935	msk
CG3936	N
CG1970	ND-49
CG11988	neur
CG3891	Nf-YA
CG1634	Nrg
CG3779	numb
CG11856	Nup358
CG2158	Nup50

CG12366	O-fut1
CG7833	Orc5
CG3479	osp
CG33105	p24-2
CG5165	Pgm
CG10108	phyl
CG17291	Pp2A-29B
CG9187	Psf1
CG18013	Psf2
CG18803	Psn
CG10975	Ptp69D
CG14039	qtc
CG17060	Rab10
CG8556	Rac2
CG3000	rap
CG5692	raps
CG10800	Rca1
CG12537	rdx
CG5313	RfC3
CG14999	RfC40
CG2161	Rga
CG8865	Rgl
CG4125	rst
CG10061	Sas-4
CG33193	sav
CG1891	sax
CG17579	sca
CG32717	sdt
CG5341	sec6
CG32120	sens
CG2621	sgg
CG14549	Sld5
CG43758	sli
CG4013	Smr
CG44436	sno
CG8978	Sop2
CG31020	spdo
CG1395	stg
CG9126	Stim
CG32130	stv

CG3497	Su(H)
CG9032	sun
CG6202	surf4
CG7417	Tab2
CG32463	Tengl2
CG6146	Top1
CG42865	trh
CG18214	trio
CG10210	tst
CG1856	ttk
CG15427	tutl
CG3172	twf
CG6235	tws
CG2257	Ubc-E2H
CG7015	Unr
CG8075	Vang
CG8821	vis
CG1520	WASp
CG10776	wit
